# The dietary risk index system: a tool to track pesticide dietary risks

**DOI:** 10.1186/s12940-020-00657-z

**Published:** 2020-10-14

**Authors:** Charles M. Benbrook, Donald R. Davis

**Affiliations:** 1 Benbrook Consulting Services, 10526 SE Vashon Vista Drive, Port Orchard, WA 98367 USA; 2 Biochemical Institute, The University of Texas, Austin, TX 78712 USA

**Keywords:** Pesticide dietary risk, Residues in food, Chronic reference dose, Acceptable daily intake, Food quality protection act, Organophosphate insecticides, Neonicotinoids, Antifungal agents, Herbicides, Glyphosate

## Abstract

**Background:**

For years the United States Department of Agriculture’s Pesticide Data Program and the United Kingdom’s Food Standards Agency have published annual or quarterly data on pesticide residues in foods. Both programs report residues in conventionally grown, organic, and imported foods. The US program has tested about 288,000 food samples since 1992, primarily fruits and vegetables consumed by children. Since 1999 the UK has tested about 72,000 samples of a wider range of foods. These data are vital inputs in tracking trends in pesticide dietary risks.

**Methods:**

The Dietary Risk Index (DRI) system facilitates detailed analyses of US and UK pesticide residue data, trends, and chronic risk distributions. The DRI value for a pesticide is the dietary intake of that pesticide from a single serving of food divided by the pesticide’s acceptable daily intake as set by the US Environmental Protection Agency. It can be calculated based on average annual residue concentrations, and on residue levels in individual samples of food. DRI values can be aggregated over multiple pesticides in single foods, and over individual pesticides in multiple foods.

**Results:**

The DRI system provides insights into the levels, trends, and distribution of pesticide dietary risk across most widely consumed foods. By drawing on both US Pesticide Data Program and UK-Food Standards Agency residue data, the DRI is capable of assessing pesticide risks in a significant portion of the global food supply. Substantial reductions in pesticide dietary risks occurred in the early 2000s, primarily from replacement of organophosphate insecticides with seemingly lower-risk neonicotinoids. However, there remain several areas of concern and opportunities to reduce risks. Both herbicide and fungicide dietary risks are rising. Organically grown produce poses risks far lower than corresponding, conventionally grown produce. Risk differences are inconsistent between domestic and imported foods.

**Conclusions:**

The surest ways to markedly reduce pesticide dietary risks are to shift relatively high-risk fruits and vegetables to organic production. For other foods, reducing reliance on pesticides overall, and especially high-risk pesticides, will incrementally lower risks. The DRI system can help focus such efforts and track progress in reducing pesticide dietary risk.

## Background

Recent science has deepened scientific and public concerns over daily exposures to pesticides, particularly dietary exposures [1–6]. Both overall reliance on pesticides relative to other pest-management tools, and the number and diversity of residues in diets have risen steadily for decades. The world’s most widely used herbicide (glyphosate) and heavily applied family of insecticides (neonicotinoids) are routinely detected in human urine [7–11].

Over the last 20 years, the desire to reduce pesticide dietary exposure has been among the top three reasons why people switch to organic foods, according to consumer surveys, and often it is the primary reason [12, 13]. The widely followed “Dirty Dozen” and “Clean 15” lists of foods issued by the Environmental Working Group [14] have raised awareness of the presence of residues in the diet. Key insights emphasized over the last 15 years in EWG’s annual “Dirty Dozen” reports include,

- Some common fruits and vegetables rarely contain pesticide residues, while other foods contain four or more residues on average, and a few contain more than 10;
- Pesticide risk sometimes differs substantially between domestically grown and imported food; and
- The surest way to markedly reduce pesticide dietary exposure and risks is to seek out and buy organically grown fresh and processed fruit and vegetable products and juices.

Extensive government pesticide residue testing of foods has been underway for about three decades. The two most sophisticated and robust of these monitoring programs are the US Department of Agriculture’s (USDA’s) Pesticide Data Program (US-PDP), and testing overseen by the Pesticide Residues Committee convened by the United Kingdom’s Food Standards Agency (UK-FSA) [15, 16].

The US-PDP has focused, since the early 1990s, on foods that make up a sizable share of the diets of infants and children, while the UK-FSA program has tested a much broader diversity of foods, food forms, and beverages since 1999, focused on residue levels and trends in the approximately 80% of the UK food supply that is imported [17].

The Dietary Risk Index (DRI) uses US-PDP and UK-FSA residue data and government-set pesticide exposure thresholds to estimate and compare the chronic risks from pesticide residues in different foods. (For the history of the DRI, see [18].) Here we review the DRI system and describe the integration of the UK-FSA residue data into it. The ability now to draw on both US-PDP and UK-FSA residue data in the DRI system supports for the first time a reasonably broad assessment of pesticide dietary-risk levels and trends across foods and pesticides in a significant share of the global food supply. The opportunity to identify low-risk and high-risk food-pesticide combinations has important ramifications for targeting regulatory interventions and guiding efforts by farmers and the food industry to incrementally lower overall pesticide dietary risks.

Currently, the US Environmental Protection Agency (EPA) relies on “Dietary Exposure Evaluation Model” (DEEM) software in conducting pesticide dietary exposure assessments [19]. DEEM combines US government food consumption survey data with various estimated or reported residues in specific foods. This deterministic model allows pesticide dietary risks to be quantified in different population cohorts (e.g., infants up to one-year old, children 1–3 years old, adult females). EPA applies DEEM to a given pesticide across all foods for which it is registered to assure that existing or proposed, new tolerances do not collectively trigger EPA’s “level of concern.” DEEM is also the key model supporting EPA dietary risk assessments during the agency’s reregistration reviews of pesticides that have been in use for a decade or more.

The DEEM model is data intensive, and EPA applies it only to pesticide-food combinations for which the agency is considering a new food use, or evaluating whether to retain an existing use and tolerance. Each DEEM run on a food-pesticide combination requires substantial work to update food consumption patterns and residue levels.

The DRI draws on much of the same data, but is designed to allow simultaneous assessment of all food-pesticide combinations based on actual, measured residues, rather than tolerances. While it would be technically possible to produce dietary risk rankings with DEEM that are comparable to the rankings produced by the DRI, doing so would be a significant undertaking and to our knowledge has never been done. Also, DEEM analyses do not utilize UK-FSA residue data, and hence the EPA lacks access to residue data on many crops and foods that are tested by the UK-FSA but not by the US-PDP.

A similar, deterministic screening tool is utilized in the European Union to estimate dietary exposures stemming from a given food-pesticide combination, taking into account the applicable Maximum Residue Level (MRL) cap on residues. Version 3 of the European Food Standards Agency (EFSA) “Pesticide Residue Intake Model” (PRIMo) is a user-friendly and Microsoft Excel-based simulation model that draws upon several sources of food consumption data to estimate pesticide exposure levels. Much like DEEM, EFSA’s PRIMo expresses exposure from a given food-pesticide combination as a percent of allowable daily intakes, either acute or chronic Acceptable Daily Intakes (ADIs) [20, 21]. The EFSA model does not allow assessment of the distribution of residue and risk levels, nor of the percent of eating episodes that are likely to expose an individual above his or her personal ADI.

DEEM, PRIMo, and the DRI system serve different purposes. DEEM and PRIMo are designed to support assessments of specific tolerances and MRLs, while the DRI allows a comparison of risks from multiple pesticides in a single serving of food, based on the residues actually present in the food. DEEM and PRIMo are deployed to make decisions about specific pesticide registrations and tolerance levels in light of how a pesticide’s label allows a particular product to be used on a given crop. The DRI is focused on identifying overall levels and trends in pesticide dietary risk, based on measured residues reported in food by the US-PDP and UK-FSA. The DRI also supports trend analyses and comparisons of risk levels as a function of how a given crop was grown (conventional vs. organic), where it was grown (e.g., imported vs. domestic), and the pesticide analytes present in a given food, or in an individual sample of food.

## Methods

A DRI value is the ratio of a pesticide’s dietary intake from a single serving of food, divided by the maximum acceptable daily intake for that pesticide, as determined by the EPA or other authority. An acceptable daily intake set by the EPA is called a “chronic Reference Dose” (cRfD), except when the agency imposes an added safety factor in response to the 1996 Food Quality Protection Act (FQPA) [22]. Conceptually, the DRI is comparable to “risk quotient” for pesticides and “hazard quotient” for air pollutants, as defined and used by the EPA and other regulators. Both are defined as a ratio, (exposure)/(a reference exposure), with exposures expressed as concentrations instead of amounts by weight [23].

The FQPA required EPA to take into account the special susceptibility to pesticides of pregnant women, infants, and children by applying an additional 10-fold safety factor when establishing or adjusting a pesticide’s cRfD. When the 10-fold FQPA safety factor is added to a pesticide’s cRfD, it becomes a chronic “Population Adjusted Dose” (cPAD). In cases where the EPA Administrator determines that existing data adequately characterizes the heightened risks facing pregnant women, infants, and children, the Administrator may reduce or remove the 10-fold FQPA safety factor. Additional files 1 and 2 show the cRfDs, cPADs, and other toxicity thresholds used in the DRI system.

Establishing a pesticide active ingredient’s cRfD or cPAD is a first, critical step the EPA must complete before judging whether proposed or existing tolerances are acceptable. For food-use pesticides, the EPA’s cRfD or cPAD and “level of concern” are reached when the agency determines there is no longer a “reasonable certainty of no harm” from that level of pesticide intake [22]. In most of the world there are similar chronic exposure thresholds, generally called ADIs [24]. They are expressed on a body-weight basis, usually in units of mg of pesticide/kg body weight/day.

The dietary intake of a pesticide from a single serving of food is determined by the pesticide’s measured concentration in the food and the serving size. We use serving sizes known as Reference Amounts Customarily Consumed per eating occasion (RACCs). See details in Additional file 3.

We calculate three types of DRI values, each providing insights into different aspects of risk levels, distributions, and trends. DRI-Mean (DRI-M) is based on the annual mean residue level of only those samples with measured residues at or above the Limit of Quantification (LOQ). The smaller Food Supply DRI (FS-DRI) is based on the annual mean residue level of all samples tested, including those with no observed residue. Single-sample DRI values pertain to a single, specific sample of food tested by the US-PDP or UK-FSA. They are valuable for assessing the distribution of residue levels and identifying the pesticides and geographic locations associated with either high-risk or low-risk samples.

### Calculating DRI values

For each year of testing, the US-PDP and UK-FSA report the following data for each tested food and pesticide:

- N Number of samples tested
- NP Number of samples in which a quantifiable residue was reported (number of positives)
- %P Percent positive (NP/N × 100%)
- Mean Mean of the positives; the annual mean residue concentration in the positive samples, always expressed in the DRI system in parts per million (ppm) by weight (e.g., mg/kg).

The DRI system uses the above data, coupled with the weight of a single serving of food (Serv) and a person’s body weight (BW), to calculate three basic DRI values, each calculated as a ratio, (dietary intake from a single serving of food)/(acceptable daily intake):

- DRI-M Positive Sample Mean DRI = (Mean × Serv)/(cRfD × BW)
- FS-DRI Food Supply DRI = (Mean × Serv)/(cRfD × BW) × N/NP = DRI-M × %P

For a single sample tested by US-PDP or UK-FSA, and for each residue reported in that sample:

- DRI = (Pesticide concentration × Serv)/(cRfD × BW)

In these equations, cRfD may be replaced by a cPAD or other ADI, as appropriate. Because they are ratios of two pesticide weights, DRI values are dimensionless.

If the amount of a pesticide in 1 serving of a food equals the maximum acceptable daily intake for that pesticide (cRfD × BW), we get a useful reference concentration we call the cRfC:

$$ \mathsf{cRfC}\times \mathsf{Serv}=\mathsf{cRfD}\times \mathsf{BW},\mathsf{or}\ \mathsf{cRfC}=\left(\mathsf{cRfD}\times \mathsf{BW}\right)/\mathsf{Serv} $$

Thus we can rewrite the above equations for DRI values to express them in terms of cRfC instead of cRfD:

- DRI-M = Mean/cRfC
- FS-DRI = Mean/cRfC × %P = DRI-M × %P
- Individual-sample DRI = (pesticide concentration)/cRfC

Several tables herein show calculated values of cRfCs, which permit easy comparison with the measured pesticide concentrations reported by the US-PDP and UK-FSA.

Although DRI (and cRfC) values can be calculated for a person of any weight, the current analysis is based on a 16-kg (35 lb.) child. Children consume a much less diverse diet than adults, eat relatively larger servings of the foods they do eat, and ingest more food per body weight than adults, in order to support growth [25]. The selection of 16 kg corresponds to children near 4-years-old, based on the 50th percentile in CDC growth charts (average of boys and girls) [26]. The child serving sizes (Serv) in the DRI system are estimated as 2/3 of the RACCs for the general population.

While cRfDs and ADIs typically apply to the general population, they are sometimes estimated for subpopulations, e.g., infants, women of childbearing age, or individuals with certain genetic polymorphisms (e.g., PON-1) [27, 28]. When subpopulation cRfDs or ADIs are available, subpopulation-specific DRI values can be calculated.

The DRI-M for a particular food-pesticide combination is an annual *average* DRI for those samples that contain measurable residues of that pesticide. Hence, DRI-M values overstate chronic risks from all samples of that food, unless 100% of samples have a quantifiable residue.

FS-DRI values are based on all samples tested, including those that do not contain detectable residues. FS-DRI values reflect annual *average*, chronic dietary exposures for those who eat a given food periodically over many days, but underestimate exposure on days when residues are present.

### Residue data and pesticide nomenclature details

Annual US-PDP data files have been downloaded and incorporated into the DRI system [29]. Beginning in 1999 the UK-FSA reported sample-specific residue data quarterly in hard-copy reports [e.g. [30],]. We moved sample identifiers and results data from these UK-FSA reports into Microsoft Access software for use in the DRI system. In 2016 the UK-FSA began providing this data in digital spreadsheet form [31].

To make the quarterly UK-FSA data compatible with annual US-PDP data, we combined the quarterly reports by calendar year. Because some crops are stored and marketed in a subsequent year, there is not a perfect match between the US-PDP and UK-FSA data sets regarding the year a crop was grown and the year it was tested for pesticide residues.

Neither the US-PDP nor UK-FSA is able to test every food every year, creating gaps between the years when a given food was tested. Technical sophistication in both programs has steadily improved, as has the number of foods, food forms, and pesticide analytes tested (e.g., pesticide metabolites and moieties). Limits of detection (LODs) and LOQs have trended downward. Various methods have been used to quantify and distinguish between residues of parent-active ingredients, various metabolites, and related moieties.

For these reasons, multiple analytical challenges arise and must be resolved in order to track changes in risk levels by crop or food, by pesticide, and by other parameters (imports vs. domestic production or conventional vs. organic production). Additional file 3 explains how the DRI system resolves pesticide name, metabolite, and moiety differences, both within the US-PDP and UK-FSA data sets over time, as well as across the two data sets. Differences between US-PDP and UK-FSA food names and food forms are addressed in Additional files 4 and 5.

In general, the UK-FSA’s program casts a wide net across the UK food supply, testing 40 to 60 foods annually. It usually tests a few to 150 samples per food or food form. The US-PDP focuses predominantly on foods making up a significant share of the diets of pregnant women, infants, and children, and tests 15 to 25 foods and food forms annually. It usually tests an average of 300 to 500 samples per food, but as many as 700 samples in a few cases (Additional file 6).

### Additional DRI system parameters and selection criteria

The DRI System incorporates and allows for selection of samples according to additional parameters in both the US-PDP and UK-FSA residue data sets. These include:

- Country of origin: Where the crop was grown, or for processed foods, the country where the final stage of manufacturing occurred (but not necessarily the source of individual ingredients).
- State of origin for some samples (US-PDP only): The US state where the crop was grown or distributed (increasingly reported over time).
- Geographic categories of food origin: Three reported by the US PDP—domestic samples, combined imports, and imports from specific countries; and four reported by UK-FSA—all samples, domestic (UK-grown), imports from other EU countries, and all other imports.
- Market claim categories based on type of production: Four in the US-PDP—organic, integrated-pest-management, pesticide free, and no claim (usually conventional); and two in the UK-FSA—organic and no claim.
- All DRI system reports can be produced in three ways, encompassing:
  1. All samples irrespective of market claim.
  2. Organic samples only.
  3. “Conventional” samples (all samples excluding “organic”).
- Rule of 10: For many imported foods, relatively few samples are tested by the US-PDP or the UK-FSA in a given year, and even fewer imported samples are available by market claim. When there are too few samples tested to place confidence in the calculated mean residue level or residue frequency, DRI system reports can be generated incorporating a “Rule of 10.” When invoked, the DRI system ignores all food-pesticide-country-of-origin-market-claim combinations with < 10 samples.
- Banned Organochlorines (OCs): Residues persist in certain foods of several OC insecticides, including DDT and its metabolites, aldrin, dieldrin, heptachlor, chlordane, mirex, and toxaphene [32, 33]. Most of these insecticides were banned for use on food crops during the 1970s, and few registered uses remain worldwide. As a result, there is little that farmers, regulators, the food industry, and pesticide manufacturers can do to prevent the occasional detection of banned OC residues. For this reason, all DRI reports can be run with or without reported detections of banned OCs. Two OCs remain in use in some countries and in the DRI system (endosulfan and methoxychlor).

### Aggregating DRI values and standard output reports

DRI-M, FS-DRI, and individual-sample DRI values are associated with individual pesticide-food combinations. These DRI values can be added together in four ways:

1. Across all foods in which a particular pesticide was found, producing an aggregate, annual pesticide-specific DRI;
2. Across all pesticides found in the US or UK samples of a specific food, creating an aggregate, annual food-specific DRI;
3. Across all pesticides found in all foods tested (or all fruits or vegetables tested), resulting in an all-foods-tested-in-a-year accounting of residues and DRI levels; and
4. Across all pesticides found in an individual sample of a particular food, creating a sample-specific, aggregate DRI.

### DRI output reports

In all reports produced by the DRI system, result tables are presented chronologically, beginning with the most-recent year of data and ending with the earliest year for which data are available. Summary statistics in the bottom rows show, among other things, the total number of residues found, the average number of residues per sample tested, and aggregate totals for DRI-M and FS-DRI.

Many DRI system reports show the share of aggregate FS-DRI that is accounted for by each food-pesticide combination. Some reports show individual food or pesticide shares of aggregate DRI-M and aggregate FS-DRI. The foods and pesticides in a DRI output table are typically ranked from the largest to smallest aggregate FS-DRI.

Displaying DRI report results in this way facilitates identification of possible risk-driver food-pesticide combinations, as well as food-pesticide combinations that contribute modestly to aggregate risk. It also provides insight into risk distributions. Usually 10% or fewer of the residues reported in a given food account for 95% or more of aggregate FS-DRI.

Drawing on either US-PDP or UK-FSA residue data, the DRI system produces multiple versions of 6 standard output reports (numbered 3–8 in the list below). Each report can be generated in Microsoft Excel, Access, or online [18]. Separate annual reports are currently available for 1992 through 2018 based on US-PDP data, and for 1999 through the first quarter of 2019 based on UK-FSA data, and include:

- Report 1 – Chemical Names, Classifications and EPA Toxicity Benchmarks (cRfDs, ADIs).
- Report 2 – Number of Samples by Food, Country of Origin, Market Claim, and Year.
- Report 3 – Food-pesticide Combination DRI Values by Year, Ranked by FS-DRI.
- Report 4 – Pesticide-food Combination DRI Values by Year, Ranked by FS-DRI.
- Report 5 – Aggregate Food DRI Values by Year, Ranked by FS-DRI across All Pesticides.
- Report 6 – Aggregate Pesticide DRI Values by Year, Ranked by FS-DRI across All Foods.
- Report 7 – Number of Residues and Aggregate DRI Values by Type of Pesticide and All Pesticides by Year.
- Report 8 – Number of Residues and Aggregate DRI Values by Food, Food Form, and Food Group by Year (under development).

The DRI system generates multiple versions of standard reports 2–8 according to combinations of the country-of-origin of the samples tested, market claims, and inclusion criteria (Rule of 10, OCs in or out) (see Additional file 3). There are seven country-of-origin options for each report:

1. All positive samples, regardless of country of origin;
2. All imported samples;
3. Imported samples disaggregated by country of origin;
4. US Domestic samples;
5. UK domestic samples (grown or processed);
6. Imported samples into the UK from other EC countries; and
7. Imported samples into the UK from non-EC countries.

The multiple versions of standard reports 2–8 also can include 3 market-claim options:

1. All samples, regardless of market claim;
2. Samples lacking a market claim, and referred to as “Conventionally Grown”; and
3. Samples labeled as organically grown.

Accordingly, the DRI system currently generates 960 output reports: 384 US-PDP reports [8 standard reports × 4 country of origin options × 3 market claim options × 4 inclusion criteria options (Rule of 10 option in or out, banned OCs in or out)], plus 576 UK-FSA reports (8 × 6 × 3 × 4). In addition, the DRI system produces thousands of tables reporting individual-sample results. Plus, each report contains several dozen to over 1000 tables with results for a given year.

## Results

The distribution of individual-sample residue levels in 10 food-pesticide combinations is shown in Table 1. The residue concentration at the 90th percentile of the distribution is typically 2 to 3 times the mean of the positives; the 95th percentile residue is usually 2.5 to 4 times the mean; and the 99th percentile residue is generally between 4 and 11 times the mean of the positives.

**Table 1:** The distribution of residue levels relative to the mean of positives for selected food-pesticide combinations: US-PDP testing of domestically grown, conventional samples

Food/Pesticide	Number of Samples	Percent Positive	Positive Sample Residue Level (ppm)					Ratio Relative to the Mean		
			Minimum	Mean of Positives	90th %	95th %	99th %	90th %	95th %	99th %
**Peaches**										
Methyl parathion (1996)	82	25.3%	0.004	0.0562	0.11	0.19	0.5	1.96	3.38	8.90
Fludioxonil (2014)	403	65%	0.0054	0.788	1.5	1.9	3.4	1.90	2.41	4.31
**Potatoes (2016)**										
Imidacloprid	295	45.2%	0.002	0.0101	0.022	0.033	0.092	2.17	3.25	9.07
Azoxystrobin	192	29.4%	0.002	0.0811	0.27	0.42	0.65	3.33	5.18	8.01
Boscalid	137	21%	0.0025	0.0039	0.0074	0.013	0.025	1.91	3.35	6.45
Phorate sulfoxide	4	0.6%	0.002	0.0571	0.12	0.12	0.12	2.10	2.10	2.10
**Tomatoes**										
Methamidophos (1996)	51	38.3%	0.002	0.0350	0.079	0.11	0.25	2.26	3.14	7.14
Endosulfan I (1998)	54	12.6%	0.003	0.00841	0.01	0.017	0.095	1.19	2.02	11.3
Endosulfan II (1998)	62	14.5%	0.005	0.0125	0.013	0.032	0.13	1.04	2.57	10.4
Bifentrhin (2015)	133	40.1%	0.002	0.00928	0.02	0.027	0.048	2.15	2.91	5.17

Based on the data in Table 1, a DRI-M value of ≤ 0.1 for a given food-pesticide combination will assure that most, but probably not all, individual samples will have a DRI value below the “level of concern” threshold of 1. A DRI-M ≤ 0.01 will assure that few if any individual samples would exceed DRI = 1. Hence the possibly high-risk zone along the risk continuum could lie above DRI-M = 0.1. The low-risk zone could fall below DRI-M = 0.01, and the “modest to moderate risk” zone would lie in between. Where a specific food-pesticide combination falls in this continuum will depend on the food, the pesticides found in it, and the distribution of residue levels, which vary year to year.

These risk thresholds are partially subjective. If the goal is to identify relatively high-risk food-pesticide combinations for regulatory interventions, the thresholds can be aligned with regulatory goals, such as EPA’s “reasonable certainty of no harm.”

### Output reports by food

Table 2 is an example of the most detailed reports generated by the DRI system. It lists all pesticides found in peaches in 1996 US-PDP testing. It shows, for example, that the US-PDP detected methyl-parathion (parathion methyl in the table) in 82 of 198 samples of domestically grown peaches in 1996, with a mean-of-positives concentration of 0.056 ppm.

**Table 2:** Residues and DRI values in US-Grown, conventional peaches tested in 1996 by the US-PDP [No Banned OC’s; Rule of 10 Imposed]

Analyte	Total Samples	Number of Positives	Percent Positive	Mean of Positives (ppm)	cRfC (ppm)	Serving Size (g)	Positive Samples DRI-M	FS-DRI	Percent FS-DRI
Parathion methyl	198	82	41.41%	0.0562	0.0320	100	1.756	0.727	69.5%
Iprodione	198	155	78.28%	0.923	8.000	100	0.115	0.0903	8.62%
Phosmet	168	53	31.55%	0.217	0.960	100	0.226	0.0713	6.81%
Dicloran	198	102	51.52%	0.394	4.00	100	0.0986	0.0508	4.85%
Carbaryl	198	32	16.16%	0.412	1.600	100	0.257	0.0416	3.97%
Azinphos methyl	198	17	8.59%	0.0539	0.240	100	0.225	0.0193	1.84%
Fenbutatin oxide	155	35	22.58%	0.137	2.72	100	0.0504	0.0114	1.09%
Propargite	198	43	21.72%	0.324	6.40	100	0.0507	0.0110	1.05%
Chlorpyrifos	198	11	5.56%	0.00809	0.0480	100	0.169	0.00936	0.894%
Dicofol p,p’	198	2	1.01%	0.0470	0.0640	100	0.734	0.00742	0.709%
Methomyl	198	3	1.52%	0.183	1.28	100	0.143	0.00217	0.207%
Myclobutanil	198	20	10.10%	0.0548	4.00	100	0.0137	0.00138	0.132%
Diazinon	198	2	1.01%	0.004	0.0320	100	0.125	0.00126	0.121%
Endosulfan sulfate	198	4	2.02%	0.0445	0.960	100	0.0464	0.000936	0.0894%
Endosulfan II	198	5	2.53%	0.0196	0.960	100	0.0204	0.000516	0.0492%
Endosulfan I	198	4	2.02%	0.0105	0.960	100	0.0109	0.000221	0.0211%
Captan	198	11	5.56%	0.0640	20.8	100	0.00308	0.000171	0.0163%
Benomyl	199	2	1.01%	0.0830	8.00	100	0.0104	0.000104	0.0100%
Thiabendazole	198	2	1.01%	0.0420	5.28	100	0.00796	0.000080	0.00768%
Imazalil	198	3	1.52%	0.0170	4.00	100	0.00425	0.000064	0.00615%
Permethrin Total	198	1	0.51%	0.480	40.0	100	0.0120	0.000061	0.00579%
DCPA	198	1	0.51%	0.0130	1.60	100	0.00813	0.000041	0.00392%
Chlorothalonil	198	1	0.51%	0.0120	3.20	100	0.00375	0.000019	0.00181%
**Average Number of Samples:**	**195**								
**Total Positives and Aggregate DRI:**		**591**					**4.092**	**1.047**	
**Average Residues Detected per Sample**		**3.03**							
**Number of Analytes Detected:**	**23**								

Methyl-parathion is an extremely toxic OP with a cRfD of 0.0002 mg/kg body weight/day. For a 100-g (3.5-oz.) serving of peach ingested by a 16-kg (35.3-lb.) child, the cRfC is 0.032 ppm, meaning that a 100-g peach can contain up to 0.032 ppm of methyl-parathion without over-exposing the child from that single peach. With an annual mean-of-positives concentration of 0.056 ppm, the DRI-M is 0.056/0.032 = 1.76. Accordingly, these 1996 peach samples should have, and surely did, trigger EPA’s “level of concern.”

Among the 82 positive samples for methyl-parathion reported by the US-PDP in domestically grown peaches in 1996, the top-10 highest concentrations ranged from 0.11 ppm to 0.50 ppm (see details in Additional file 7). The highest individual-sample methyl parathion residue (0.50 ppm) was grown in California, and had a DRI of 15.6, far above EPA’s “level of concern.”

In addition to methyl-parathion, Table 2 shows corresponding results for 22 other pesticides found in US-grown, conventional peaches in 1996, plus the aggregate total DRI-M and FS-DRI for all 23 pesticides (4.092 and 1.047 respectively). The last column shows that methyl-parathion alone accounted for 69% of that year’s aggregate FS-DRI across all pesticides detected in peaches by the US-PDP.

This finding illustrates why EPA placed such high priority on ending methyl-parathion uses on peaches and other soft-skinned fruit and vegetable crops as it implemented the FQPA. This goal was largely accomplished by the end of 2001; the last peach sample found by the US-PDP to contain a methyl-parathion residue was from a domestic sample in 2008.

Another key characteristic of pesticide dietary risks is illustrated in Table 2 by the highly skewed distribution of DRI values across pesticides. Among the 23 different pesticides detected, 15 (65%) had FS-DRI values below 0.01 and collectively accounted for < 1% of aggregate FS-DRI.

An important distinction between DRI-M and FS-DRI is clear in Table 2 for dicofol p,p’ and other pesticides with low frequencies of detection (small %P). Only 2 out of 198 peach samples had quantified dicofol p,p’ residues. Their mean concentration of 0.047 ppm yields a DRI-M of 0.73, meaning that children consuming peaches with dicofol at this level would average 73% of their daily cRfD from a single peach (100 g). But averaged across the 198 samples tested, the FS-DRI is only 0.0074.

Iprodione, a post-harvest fungicide used in peach packing houses, was the second highest contributor to aggregate FS-DRI in Table 2, detected in 78% of US peach samples, with a mean-of-positives concentration of 0.923 ppm and a DRI-M of 0.115. Despite being 250-times less toxic than methyl-parathion (measured by its cRfD), iprodione’s higher concentration yielded a DRI value only 15-times lower than methyl parathion’s.

### Aggregate DRI levels across foods

In 1996, domestically grown peaches, apples, and grapes accounted for the largest shares of aggregate FS-DRI across the 15 foods tested that year by the US-PDP. Collectively these three foods accounted for 61% of aggregate FS-DRI, contributing respectively 28, 19, and 14% (see Additional file 8).

Most conventionally grown, thin- or soft-skinned fruits like peaches, nectarines, pears, cherries, plums, and berries have relatively high DRI-M and FS-DRI scores, regardless of residue data set (US or UK), year tested, and country of origin. But other crops like onions, carrots, pineapples, oranges, and nuts tend to have consistently lower DRI values. Table 3 shows a relatively low-risk example from the UK-FSA, spring onions. In 2016, 38 samples of UK-grown, conventional spring onions contained 13 pesticides with an aggregate FS-DRI of only 0.00087 and an aggregate DRI-M of 0.017.

**Table 3:** Residues and DRI values in UK-Grown, conventional spring onions, tested in 2016 by the UK-FSA [No OC’s Banned by US included]

Analyte	Total Samples	Number of Positives	Percent Positive	Mean of Positives (ppm)	cRfC (ppm)	Serving Size (g)	Positive Samples DRI-M	FS-DRI	Percent FS-DRI
Propamocarb	38	1	2.63%	0.900	9.60	16.7	0.00781	0.000206	23.7%
Cyprodinil	38	3	7.89%	0.0467	25.9	16.7	0.00180	0.000142	16.4%
Iprodione	38	5	13.2%	0.034	48.0	16.7	0.000708	0.0000932	10.8%
Fludioxonil	38	3	7.89%	0.030	28.8	16.7	0.00104	0.0000822	9.49%
Azoxystrobin	38	10	26.3%	0.053	173	16.7	0.000307	0.0000807	9.31%
Tefluthrin	38	1	2.63%	0.010	4.80	16.7	0.00208	0.0000548	6.32%
BAC	38	5	13.2%	0.032	96.0	16.7	0.000333	0.0000439	5.06%
Dimethomorph	38	8	21.1%	0.0188	96.0	16.7	0.000195	0.0000411	4.74%
Tebuconazole	38	2	5.26%	0.020	27.8	16.7	0.000718	0.0000378	4.36%
DDAC	38	5	13.2%	0.026	96.0	16.7	0.000271	0.0000356	4.11%
Chlorothalonil	38	1	2.63%	0.020	19.2	16.7	0.00104	0.0000274	3.16%
Fluopicolide	38	1	2.63%	0.090	192.0	16.7	0.000469	0.0000123	1.42%
Boscalid	38	2	5.26%	0.040	209.3	16.7	0.000191	0.0000101	1.16%
**Average Number of Samples:**	**38**								
**Total Positives and Aggregate DRI:**		**47**					**0.0170**	**0.000867**	
**Average Residues Detected per Sample**		**1.24**							
**Number of Analytes Detected:**	**13**								

The carbamate insecticide propamocarb is the dominant contributor to aggregate FS-DRI in Table 3. It was detected in just 1 sample at the relatively high concentration of 0.90 ppm. Because of its low chronic toxicity (cRfD = 0.12 mg/kg body weight/day), the DRI-M associated with this one positive sample was 0.0078, and the FS-DRI was very low (0.00021). The tested samples contained on average 1.2 residues. Of the 47 residues shown in Tables 3, 79% are fungicides [34].

### Output reports by pesticide

The DRI system produces pesticide-specific reports comparable to the food-specific reports discussed above. We illustrate two examples here—glyphosate residues in conventional foods tested by the UK-FSA in 2016 (Table 4), and chlorpyrifos in conventional, US-grown foods tested by the US-PDP in 1999 (Table 5).

**Table 4:** Residues of glyphosate and DRI values in UK-Grown, conventional foods tested in 2016 by the UK-FSA [No OC’s Banned by US included]

Food	Sub-Food	Total Samples	Number of Positives	Percent Positive	Mean of Positives (ppm)	cRfC (ppm)	Serving Size (g)	Positive Samples DRI-M	FS-DRI	Percent FS-DRI
Breakfast Cereal	Oats	13	13	100.0%	0.58	1050	26.7	0.000549	0.000549	38.24%
Rye Grain	Not Specified	24	5	20.8%	0.96	988	28.4	0.000972	0.000203	14.10%
Breakfast Cereal	Bran	32	26	81.3%	0.25	1050	26.7	0.000242	0.000196	13.67%
Breakfast Cereal	Not Specified	12	9	75.0%	0.19	1050	26.7	0.000180	0.000135	9.39%
Gluten Free Food	Free-from cereal	13	3	23.1%	0.50	955	29.3	0.000524	0.000121	8.41%
Ordinary Bread	Wholemeal	34	12	35.3%	0.28	933	30.0	0.000304	0.000107	7.46%
Ordinary Bread	Other	20	7	35.0%	0.17	933	30.0	0.000184	0.0000643	4.48%
Breakfast Cereal	Wheat	30	3	10.0%	0.40	1050	26.7	0.000381	0.0000381	2.65%
Ordinary Bread	White	87	8	9.2%	0.13	724	38.7	0.000134	0.0000123	1.11%
Specialty Bread	Pitta	19	1	5.3%	0.10	741	37.8	0.000135	0.00000711	0.50%
**Total Number of Samples:**		**284**								
**Total Detections and DRI Across All Foods:**			**87**					**0.0036**	**0.0014**	
**Average Residues Detected per Sample:**			**0.31**							
**Number of Foods with Glyphosate Detected:**		**10**

**Table 5:** Residues and DRI values for Chlorpyrifos Detected in US-Grown, conventional foods in 1999 PDP testing [Rule of 10 imposed]

Food	Total Samples	Number of Positives	Percent Positive	Mean of Positives (ppm)	cRfC (ppm)	Serving Size (g)	Positive Samples DRI-M	FS-DRI	Percent FS-DRI
Apples-Single Servings	1354	416	30.7%	0.0374	0.0483	99	0.774	0.2378	52.9%
Apples	360	79	21.9%	0.0207	0.0483	99	0.428	0.0938	20.9%
Sweet Bell Peppers	515	24	4.7%	0.0815	0.0783	61	1.041	0.0485	10.8%
Pears-Single Servings	275	2	0.7%	0.119	0.0404	119	2.93	0.0213	4.74%
Pears	279	2	0.7%	0.0785	0.0404	119	1.94	0.0139	3.10%
Tomatoes	219	8	3.7%	0.0133	0.0500	96	0.265	0.0097	2.16%
Spinach, Frozen	693	47	6.8%	0.0114	0.0923	52	0.123	0.0084	1.86%
Cucumbers	377	4	1.1%	0.0430	0.0785	55	0.496	0.0053	1.17%
Strawberries	602	6	1.0%	0.0230	0.0500	96	0.460	0.0046	1.02%
Cantaloupe	548	9	1.6%	0.0122	0.0450	107	0.272	0.0045	0.99%
Tomatoes, Canned	352	6	1.7%	0.0050	0.0595	81	0.0840	0.0014	0.32%
**Total Number of Samples:**	**5574**								
**Total Detections and DRI Across All Foods:**		**603**					**8.81**	**0.449**	
**Average Residues Detected per Sample:**		**0.108**							
**Number of Foods with Chlorpyrifos Detected:**	**11**								

In 2016 the UK-FSA tested 284 samples of 10 breads and cereals. A total of 87 samples contained glyphosate (31%). Residues were most common in breakfast cereals made with oats (100%), bran (81%), and “Not-Specified” ingredients (75%). The mean residue concentrations in these 3 types of cereal ranged from 0.19 to 0.58 ppm. Because of glyphosate’s very high cRfD at the time (1.75 mg/kg body weight/day), the cRfCs for these cereals were also very high—1050 ppm, resulting in DRI-M values < 0.001 and FS-DRI < 0.0001. Glyphosate in these grain-based products nearly always comes from applications shortly before harvest, used to accelerate the death and desiccation of the plants, so harvest operations can be completed before the arrival of inclement fall weather.

As shown in Table 5, the US-PDP in 1999 detected chlorpyrifos in 11 fruit and vegetable food forms, including two forms of apples and pears (composite samples and single servings). Out of 5574 samples, 603 tested positive (10.8%) for chlorpyrifos. DRI-M values exceeded 0.1 for 10 of the 11 foods. The DRI-M exceeded 1.0 for bell peppers and pears.

### Residues and risks by country of origin

Tables 6 and 7 present two types of DRI reports comparing residues and DRI levels in imported vs. domestic foods. Table 6 compares UK-grown and imported conventional potatoes in 2013. The UK-FSA tested 102 samples of UK-grown “main crop” potatoes and 13 samples of imported potatoes. The plant-growth regulator chlorpropham is used to inhibit sprouting. It was detected in 26% of the UK-grown potatoes and 69% of the imported potatoes, with mean-of-positive sample concentrations of 1.55 ppm and 0.93 ppm respectively, and DRI-M values of 0.15 and 0.088.

**Table 6:** Residues and DRI values for UK-Grown and imported, conventional potatoes tested by the UK-FSA in 2013 [No OC’s Banned by US included]

Analyte	Total Samples	Number of Positives	Percent Positive	Mean of Positives (ppm)	cRfC (ppm)	Serving Size (g)	Positive Samples DRI-M	FS-DRI	Percent FS-DRI
**UK-Grown**									
Chlorpropham (potato def.)	102	26	25.5%	1.55	10.7	75	0.146	0.0371	45.3%
Maleic Hydrazide	102	20	19.6%	9.58	53.3	75	0.180	0.0352	43.0%
Fosthiazate	102	1	1%	0.020	0.0360	75	0.561	0.00550	6.72%
Thiabendazole	102	1	1%	2.00	7.04	75	0.284	0.00279	3.40%
Pencycuron	102	6	5.9%	0.025	2.13	75	0.0117	0.000689	0.842%
Imazalil	102	1	1%	0.300	5.33	75	0.0563	0.000551	0.065%
Propamocarb	102	6	5.9%	0.0233	25.6	75	0.000911	0.0000536	0.673%
Azoxystrobin	102	1	1%	0.0200	38.4	75	0.000521	0.00000511	0.006%
**Average Number of Samples:**	**102**								
**Total Positives and Aggregate DRI:**		**62**					**1.240**	**0.0819**	
**Average Residues Detected per Sample:**		**0.608**							
**Number of Analytes Detected:**	**8**								
**Imported to UK**									
Chlorpropham (potato def.)	13	9	69.2%	0.933	10.7	75	0.0875	0.0606	66.0%
Maleic Hydrazide	13	2	15%	8.800	53.3	75	0.165	0.0254	27.30%
Thiabendazole	13	1	7.7%	0.600	7.04	75	0.0852	0.00656	7.04%
Imazalil	13	1	7.7%	0.040	5.33	75	0.00750	0.000577	0.62%
Propamocarb	13	1	7.7%	0.020	25.6	75	0.000781	0.0000601	0.065%
**Average Number of Samples:**	**13**								
**Total Positives and Aggregate DRI:**		**14**					**0.346**	**0.0932**	
**Average Residues Detected per Sample:**		**1.08**							
**Number of Analytes Detected:**	**5**

**Table 7:** Pesticide residue and risk indicators in conventional domestic (US-Grown) and conventional combined imports of potatoes tested by the US-PDP in 2015

	Average Number of Samples per Pesticide	Total Number of Residues Found	Percent of Samples with Zero Residues	Average Number of Residues per Sample	DRI-M	FS-DRI
Domestic Samples	658	1763	0.152%	2.68	3.90	0.204
Combined Imports	23	58	0.0%	2.53	0.558	0.099
Ratio of Domestic to Imported	28.6	30.4	–	1.06	6.99	2.06

Table 7 compares US-grown and imported conventional potatoes tested by the US-PDP in 2015. It shows the number of samples tested, number of residues found, average number of residues per sample, and aggregate values for DRI-M and FS-DRI. The 3rd row of data shows the ratios, domestic/imported, for these data. In that year, imported potatoes contained roughly the same average number of residues per sample tested, but posed markedly lower DRI values compared to US-grown samples. Three of the 660 US-grown potato samples tested that year contained the oxygen analog of parathion at a mean concentration of 0.020 ppm (see Additional file 9). These three samples had a DRI-M value of 3.13, and accounted for 80% of the aggregate DRI-M shown in Table 7 for domestic potatoes in 2015. Because of a low %P (0.45%), the three parathion metabolite residues had a much lower FS-DRI value, narrowing the difference between FS-DRI and DRI-M values for imported compared to domestically grown potatoes.

### Impacts of conventional and organic production systems

In the US, UK, and most countries worldwide, organic farmers are allowed to use “natural” pesticides that are typically derived from microorganisms, natural elements like copper and sulfur, and insect semiochemicals (pheromones). With few exceptions, synthetic chemical pesticides are not allowed for use in organic production systems, and hence should never, or rarely, appear as a residue in certified organic food. Yet residues of synthetic pesticides are sometimes detected in organic foods. Common examples include:

- Residues of persistent, legacy insecticides like DDT and chlordane not applied on food crops since the 1970s, which are still detected in some soils and root crops grown worldwide.
- Low concentrations of post-harvest fungicides used in packing plants processing both conventional and organic produce, especially fruits.
- Low concentrations of synthetic pesticides that drift or otherwise move from a conventionally managed field onto organic crops growing nearby.

The DRI system generates three issue-specific reports designed to highlight the impact of organic vs. conventional management on residues and DRI values of a given crop. Table 8 summarizes the residues detected in organic and conventional apples grown in the US in 2009. Among 571 conventional samples, the US-PDP found an average of > 6 residues, compared to 1.5 residues in 22 samples of organic apples.

**Table 8:** Residues and DRI risk levels in organic and conventional apples grown in the US in 2009

	Total Samples	Total Number of Residues Found	Percent of Samples with Zero Residues	Average Number of Residues per Sample	DRI-M	FS-DRI
Conventional	571	3522	1.05%	6.16	11.8	0.223
Organic	22	34	31.3%	1.52	0.00328	0.000874
**Ratio of Conventional to Organic**	**26.0**	**104**	**0.0340**	**4.06**	**3584**	**255**

1. Results from 571 conventional and 22 organic samples

Both the aggregate DRI-M and FS-DRI values for US-grown conventional apples were clearly worrisome in 2009, while the corresponding values for organic apples were much lower, reduced by factors of over 3500 and 250, respectively. Recall that DRI-M values obscure the much higher risks associated with individual samples of apples at the high end of the risk distribution. In 2009, the highest-risk US-grown apple sample had an aggregate DRI value of 8.75 (see Additional file 10).

The top FS-DRI risk-driver in conventional apples in 2009 was the post-harvest fungicide thiabendazole. It was found in 78% of 672 samples tested, with a mean-of-positives concentration of 0.39 ppm, yielding an FS-DRI of 0.058. Two OPs—diazinon and azinphos-methyl—accounted for another 33% of aggregate FS-DRI. One sample was positive for dicofol p,p’ at a relatively high concentration of 0.56 ppm, leading to a DRI-M value of 8.7, 73% of aggregate DRI-M across the 52 residues detected in conventional apples.

Among the 34 residues detected in the 22 organic samples, 3 were the bioinsecticide spinosad, which is approved for organic use. The 31 others were very low levels of post-harvest fungicides used in packing sheds and storage areas where conventional fruit is processed or stored (diphenylamine, pyrimethanil, and thiabendazole). See Additional file 11 for detailed tables on organic and conventional US-grown apples tested by the PDP in 2009.

### Assessing food-supply residue frequency and risk levels

The DRI system produces two standard series of reports, with aggregate DRI values calculated either by food or for all foods tested in a given year, or by pesticide across all pesticides tested for in a given year. As with all DRI tables, these are available for all combinations of market claim and country of origin, with or without the Rule of 10 or banned OCs.

In any given year, the number of foods tested, and the selection of foods, will strongly affect the sum of aggregate DRI values across all foods. The impact of food selection is especially significant in the US-PDP, given its focus on foods with significant shares of dietary exposures and risks among pregnant women, infants and children (mostly fruit and vegetable-based foods).

Table 9 summarizes US-PDP findings and aggregate DRI values by food and for all foods tested in 2016, a year in which residues were detected in 21 foods, 19 of which were fruit and vegetable based. The two animal products tested—milk and eggs—yielded only 20 positive detections out of 24,658 tests, and contributed negligibly to aggregate annual DRI-M and FS-DRI.

**Table 9:** Residue frequency and aggregate DRI values by food for US-Grown, conventional samples tested by the US-PDP in 2016

Commodity	Average Number of Samples	Number of Positives	Average Number of Residues per Sample	Positive Samples DRI-M	Percent of Total DRI-M	Total FS-DRI	Percent of Total FS-DRI
Spinach	549	4672	8.52	4.50	18.2%	0.763	31.8%
Green Beans	363	693	1.91	4.78	19.4%	0.372	15.5%
Cherries, Frozen	62	490	7.9	1.08	4.38%	0.303	12.6%
Potatoes	651	1769	2.72	1.62	6.55%	0.224	9.31%
Pears	586	2711	4.63	1.89	7.67%	0.193	8.03%
Apples	481	2189	4.55	1.85	7.48%	0.160	6.66%
Strawberries	451	3830	8.49	2.20	8.92%	0.129	5.35%
Lettuce	707	1676	2.37	2.91	11.8%	0.0637	2.65%
Sweet Potatoes	508	464	0.91	0.624	2.53%	0.0519	2.16%
Grapes	336	1698	5.05	0.544	2.20%	0.0396	1.65%
Tomatoes	206	769	3.73	0.482	1.95%	0.0221	0.921%
Cucumbers	248	405	1.63	1.55	6.27%	0.0213	0.889%
Oranges	611	1065	1.74	0.0625	0.253%	0.0193	0.803%
Apple Sauce	166	663	3.99	0.107	0.435%	0.0168	0.700%
Cranberries	119	82	0.69	0.321	1.30%	0.0123	0.513%
Grapefruit	632	1210	1.92	0.0424	0.172%	0.0078	0.323%
Tomatoes, Canned	164	190	1.16	0.117	0.476%	0.0026	0.109%
Olives, Canned	145	55	0.38	0.00483	0.0196%	0.00079	0.033%
Cranberries, Frozen	20	7	0.35	0.00550	0.0223%	0.00030	0.012%
Milk	632	18	0.03	0.00169	0.00684%	0.000048	0.0020%
Eggs	271	2	0.01	0.000209	0.000845%	0.0000015	0.000064%
**Sum of Average Number of Samples:**	**7908**						
**Total Positives and Aggregate DRI:**		**24,658**		**24.7**		**2.40**	
**Average Residues Detected per Sample:**		**3.12**					
**Number of Foods with Residues:**	**21**						
**Average DRI per Food with Residues:**				**1.18**		**0.114**	

For all 21 foods with one or more detected residues, the 2016 totals for DRI-M and FS-DRI were 24.7 and 2.40 respectively. Spinach accounted for 31.7% of the aggregate FS-DRI (0.76), green beans (15.5%, 0.37), and frozen cherries (12.6%, 0.30). These top-three foods accounted for 59.8% of total FS-DRI for 2016. Eleven other foods each accounted for < 1% of annual FS-DRI.

In 2016 the 21 foods averaged 3.1 residues per sample. Spinach and strawberries had the most residues, each averaging 8.5 residues per sample.

Table 10 summarizes UK-FSA residue detections and aggregate DRI values for the top 20 pesticides ranked by aggregate FS-DRI across all foods tested, as well as summary data for all 102 pesticides detected. It reflects domestically (UK) grown, conventional crops in 2016. A total of 1624 positive residues were detected across 102 pesticides. The desiccant chlormequat was by far the most common residue detected. It was found on 82% of the samples of crops that contained this residue, and accounted for 18% of the residues of all pesticides detected in 2016.

**Table 10:** Residue frequency and aggregate DRI values for the Top 20 pesticides found in UK-grown, conventional samples in 2016, ranked by FS-DRI

Pesticide	Family of Chemistry	Total Samples Across All Foods	Number of Positives	Percent Positive	Positive Samples DRI-M	Percent of Total DRI-M	Total FS-DRI	Percent of Total FS-DRI
Pirimiphos-Methyl	organophosphate	296	36	12.2%	14.3	68.4%	1.76	49.6%
Lambda-Cyhalothrin	pyrethroid	18	6	33%	0.611	2.91%	0.319	8.97%
Profenofos	organophosphate	22	12	55%	0.416	1.98%	0.227	6.38%
Pirimicarb	carbamate	218	11	5.1%	1.28	6.11%	0.199	5.59%
Thiacloprid	neonicotinoid	81	19	24%	0.329	1.57%	0.122	3.43%
Cyprodinil	anilinopyrimidine	163	40	25%	0.253	1.21%	0.118	3.32%
Chlormequat	quarternary ammonium	357	293	82%	0.120	0.57%	0.102	2.87%
Chlorpropham	carbamate	137	55	40%	0.256	1.22%	0.0881	2.48%
Fludioxonil	phenylpyrrole	184	45	24%	0.186	0.89%	0.0864	2.43%
Propamocarb	carbamate	259	30	12%	0.0830	0.40%	0.0581	1.64%
Maleic Hydrazide	pyridazine	123	33	27%	0.207	0.99%	0.0554	1.56%
Deltamethrin	pyrethroid	267	15	5.6%	0.181	0.86%	0.0328	0.92%
Bupirimate	pyrimidinol	60	24	40%	0.0657	0.31%	0.0322	0.91%
Dithiocarbamates	disulfur compounds	71	17	24%	0.152	0.72%	0.0321	0.90%
Azoxystrobin	strobilurin	357	63	18%	0.0792	0.38%	0.0312	0.88%
Tebuconazole	triazole	326	40	12.3%	0.0750	0.52%	0.0255	0.72%
Chlorate	inorganic	131	27	21%	0.0785	0.36%	0.0223	0.63%
Propyzamide	benzamide	23	4	17%	0.079	0.37%	0.0196	0.55%
Pyraclostrobin	strobilurin	117	40	34%	0.0452	0.22%	0.0180	0.51%
Iprodione	dicarboximide	174	32	18%	0.0359	0.17%	0.0133	0.37%
**Totals Top 20 Pesticides:**			**842**		**18.9**		**3.36**	
**Totals 82 Other Pesticides:**			**782**		**2.13**		**0.191**	
**Totals All Pesticides:**			**1624**		**21.0**		**3.56**	

The OP and crop storage insecticide pirimiphos-methyl appeared on 12% of the crop samples containing this residue, and accounted for 50% of aggregate FS-DRI in 2016. Its aggregate DRI-M was 14.3, accounting for 76% of total DRI-M that year. Of the 102 pesticides detected, only 11 contributed 1% or more to aggregate FS-DRI, while 78 pesticides had FS-DRI values < 0.01.

## Discussion

The DRI system is designed to estimate pesticide dietary risks across foods and pesticides, and over time. The accuracy of DRI values depends on: (a) the extent and accuracy of residue data, and (b) the completeness, quality, and interpretation of the toxicology data used to set cRfDs, cPADs, and cADIs. As more refined dietary exposure thresholds emerge, they can be moved into the DRI system and used to recalculate DRI-M, FS-DRI, individual-sample DRI values, and all DRI system reports.

Systems designed to track pesticide dietary risks ideally should take into account several other factors: multiple residues in a given sample of food; variability in a food-pesticide residue profile; and differences in susceptibility across the population, associated with health status, other chemicals to which a person is exposed, and whether a person has a genetic polymorphism that impacts pesticide metabolism or toxicity.

### General features of the DRI

DRI-M and FS-DRI values for a single food-pesticide combination are based on *annual average* residue concentrations. Some consumers will ingest food with residue levels below the average, while others will ingest higher levels. In addition, consumers often ingest residues of specific pesticides in multiple foods in the same day.

The US-PDP and UK-FSA typically measure pesticide concentrations in composite samples drawn from several different bags or bins of the same food in a distribution terminal or warehouse. Each composite sample weighs about 5 lb. (2–3 kg) and might contain fruit or vegetables from several different fields, farms, and even regions of the country. Some composite samples are found to contain a dozen or more residues. It is unlikely that any individual field was treated with so many pesticides. To address this issue the US-PDP tested both composite and single-serving samples of apples (1999), pears (1998–1999), and peaches (2000). Single-serving samples of pears in both years contained notably fewer residues on average than the composite samples: 1.0 residue per sample vs. 4.8 in 1999, and 0.83 vs. 2.6 in 1998. There were more modest differences for apples and peaches.

### Taking stock of residues on multiple foods

Many pesticide active ingredients are sprayed and detected on multiple crops in a given year. Few pesticides are likely to be present in 10 different foods consumed by an individual in a given day, so adherence to a DRI-M limit of ~ 0.1 in each food should, on average, largely prevent excessive exposures to any given pesticide on most days. However, a few samples of a single food with a DRI-M = 0.1 may still pose much higher risks, because individual-sample DRI values are sometimes well over 10-fold higher than corresponding DRI-M values. Hence, some portion of the supply of a food with DRI-M = 0.1 in a given year will likely exceed DRI-M = 1, the EPA’s regulatory “level of concern.”

Pesticide laws and policy directives in the US and EU direct regulators to take into account cumulative exposures across multiple pesticide active ingredients in cases where structurally related active ingredients may pose human risks through a common mechanism of biological action [e.g., the FQPA in the US [22]]. Thus in the US the EPA pioneered methods to conduct a cumulative organophosphate (OP) exposure- and risk-assessment encompassing over 100 food uses of 26 OPs [35]. In 2000, the US-PDP reported 2432 residues of 20 different OP insecticides in 23 US-grown foods, with an aggregate DRI-M of 30 and an aggregate FS-DRI of 2.03. Because of the relatively high risks from OPs in many common children’s foods [32, 33], the EPA focused almost exclusively on the OPs in the early years of FQPA implementation.

### Interpreting DRI-M and FS-DRI values

It is a complex task for EPA to set exposure thresholds (its “level of concern”) and to determine whether to approve or deny a new food use of a pesticide. The EPA has little or no capacity to track changes in overall pesticide dietary risks over time, or as a function of country of origin or production system. The DRI system provides a mechanism to track risk levels across large residue data sets encompassing many crops, pesticides, regions, production systems, and years. But in a given DRI-based risk ranking of foods or individual samples, it remains a challenge to delineate high and possibly worrisome risks from seemingly low risks not worthy of more in-depth focus by regulators.

Along any pesticide dietary risk continuum, there is:

- A “deminimus risk” zone for very-low and zero-risk samples,
- A “modest to moderate risk” zone with positive samples posing risks above the “deminimus risk” threshold and below the possibly significant risk, “level-of-concern” threshold, and
- A “significant risk” zone where a combination of residue levels, the frequency of residues across the food supply, and pesticide toxicity suggest that some consumers will be exposed on some days above their personal cRfD or ADI, thereby exceeding the “reasonable-certainty-of-no-harm” standard governing pesticide residues in food.

There is no single and correct way to set the “low risk” vs. “modest to moderate risk” vs. “significant” DRI risk thresholds for a food-pesticide combination. Such choices can be informed through analyses of the distribution of residue and risk levels, coupled with decisions about the percent of exposure episodes that can, as a matter of policy, exceed a particular risk threshold.

The EPA has interpreted the FQPA’s “reasonable certainty of no harm” to mean that the daily exposure level for a given pesticide at the 99.9th percentile of its exposure distribution should be below the pesticide’s cRfD or cPAD. This “Threshold of Exposure” was set forth in a March 22, 2000 Federal Register Notice, “Policy Issues Related to the Food Quality Protection Act” [36]. Hence, pesticide intake at the 99.9th percentile of the exposure-risk curve would have a DRI ≥ 1.

It is more difficult and inherently subjective to define risk thresholds based on DRI-M and FS-DRI levels, because both are based on a point estimate of mean-residue levels, and provide no insights into the upper end of residue distributions. For example, a food-pesticide combination with DRI-M or FS-DRI = 0.1 could contain individual samples with a DRI well over 1.

### Accuracy and limits of the DRI system

DRI values are only as accurate and meaningful as the data used to compute them. In general, greater confidence can be placed in the DRI’s numerator—the mean residue concentration used to calculate DRI-M and FS-DRI values, and the residue concentrations in individual-sample DRIs—than in the human pesticide safety threshold in the DRI denominator (the pesticide active ingredient’s cRfD/cPAD/cADI, or derived cRfC).

High quality, extensive pesticide residue concentrations are now available from the US-PDP and UK-FSA and have been incorporated in the DRI system. Both testing programs emphasize frequently consumed foods that typically contain residues, and test relatively few foods known to rarely contain residues. Both data sets permit risk analyses at several levels of aggregation, as well as risk levels in samples from specific geographic regions and in food harvested from organic vs. conventional production systems. Sample-specific results show the distribution of residue levels.

The human safety thresholds are typically based on chronic feeding studies in mice and rats that are designed to detect adverse impacts on many toxicological endpoints, but not all. As new data identify improved safety thresholds, they can be incorporated into the DRI system.

The risk-assessment methods used by EPA and European regulators produce a single estimate of a pesticide’s chronic toxicity, based on the adverse response that occurs at the lowest dose among all toxic effects observed. Some pesticides pose one, or just a few, types of risk, the worst of which may quickly dissipate soon after exposures end. Other pesticides may cause several possible adverse effects, some of which may be essentially irreversible. Some types of adverse effects are well explored, while other, emerging toxicity endpoints are not (e.g., impacts on the microbiome and epigenetic effects).

Thus many, if not most, EPA-set cRfDs and EFSA’s chronic or acute ADIs do not take account of the diversity of adverse biological outcomes that a given pesticide might cause. Nonetheless, regulators have the tools needed to assure that existing risk thresholds are rarely, if ever, exceeded. The fact that *other* risks may exist from exposures to the same pesticide does not diminish the need to mitigate known risks.

Significant progress has been made in the last two decades in reducing reliance on some high-risk pesticides. Dietary risks from insecticide residues in the US, in particular, have declined since the late 1990s, largely as a result of passage of the FQPA in 1996 and the phase-out of dozens of permitted uses of OPs on fresh fruits and vegetables. However, fungicide residues and risks are rising. In recent years, about one-third of all pesticide residues detected in US-PDP testing come from post-harvest uses of fungicides in packing plants. Moreover, the concentrations of post-harvest fungicides on many fresh fruits and vegetables are high compared to fungicide residues stemming from field applications. This is unsurprising, because post-harvest fungicides are often applied to fresh produce right before it is shipped to retail outlets or food service businesses.

Although modest compared to risks from insecticides and fungicides, herbicide residues and risk levels are rising in certain foods, drinking water, and some beverages. Reliance on four herbicides associated with genetically engineered, herbicide-tolerant crops (glyphosate, glufosinate, 2,4-D, and dicamba) has risen significantly since the early 1990s [34, 37]. Rising use has been driven by the spread of weeds that have become resistant to once-effective herbicides, especially glyphosate. Each of these four herbicides is associated with combinations of reproductive risks [7, 38–41], birth defects [42–48], and cancer [49–53].

Moreover, some broad-spectrum herbicides are increasingly applied late in crop-production cycles of grains and edible beans as a pre-harvest desiccant to accelerate the drying of crops and hasten harvest operations. Such applications routinely result in residues in grain or harvested crops that are 10- to 100-fold or more higher than the residues present from typical early-season herbicide applications.

There is a general trend toward more biologically-based modes of action in controlling insects, as opposed to simply poisoning as many insect species as possible with one, broad-spectrum insecticide. This trend is behind the steady increase over two decades in the number of insecticides applied, and the number of their residues detected in food by the US-PDP and UK-FSA.

DRI system results show clearly that relatively few foods—mostly fresh fruits and vegetables—account for the lion’s share of pesticide dietary risks, while grains and animal products account for generally modest risks. Within fruits and vegetables, soft-skinned produce tends to be most vulnerable to pest attack, and hence conventional farmers producing these crops tend to rely heavily on pesticides.

It is also clear now that the pesticide risks in US-grown or UK-grown foods are not necessarily lower than in imported foods. By tracking residues in foods imported into the US and UK, the DRI system gives consumers, food companies, the pesticide industry, and regulators useful information for avoiding imported foods with a history of sometimes high and erratic residue profiles, while also identifying food sources with reliably favorable residue profiles, like conventionally grown tree fruits from Canada and New Zealand.

The DRI system also provides powerful tools to compare the residues in organic vs. conventional foods, and the risks stemming from them. There is ample data now from the US-PDP and UK-FSA to quantify the generally sizable pesticide risk reduction that consumers can expect when choosing certified organic fruit and vegetable products. Organic farming systems allow use of some biologically based pesticides, a few of which leave residues on food, but most residues in organic food are from either post-harvest fungicides inadvertently picked up in packing plants that also process conventionally-grown produce, or are other “inadvertent residues” not applied on the field where the organic crop was grown. Such residues are typically present in organic foods at concentrations 10- to 100-fold or more lower than in conventional samples of the same crops.

The contribution of organic foods to overall pesticide risks in the US and much of the global food supply is trivial compared to the risks routinely associated with three common application scenarios:

1. Conventionally grown leafy greens and several soft-skinned fruits.
2. Any crop or food sprayed with a pesticide to speed up harvest, extend shelf life, or prevent insect or mold damage during storage.
3. Crops grown in a field previously treated with persistent soil insecticides, nematicides, and fumigants.

Unfortunately, some of the highest DRI values associated with specific food-pesticide combinations are not currently on the radar screen of the food industry, consumers, or the public health community, because regulators are no longer, or never have, focused on them (e.g., soil fumigant residues in follow-on crops, post-harvest fungicides). This is an example of a system failure and emerging challenge that can be addressed via applications of the DRI system, and then tracked over time as efforts are made to reduce exposures and risk levels.

DRI system outputs can be improved by adding additional sources of high-quality residue data and more accurate, robust toxicity thresholds. Potential sources of residue data include the extensive, EU-wide pesticide residue data set maintained by the EFSA [54], the “Total Diet Study” residue data generated by the US Food and Drug Administration [55], and other sources such as food companies carrying out residue monitoring programs.

All pesticide cRfDs and cADIs are based on a single adverse impact in animal studies. For several major pesticides, the underlying studies are decades old, and might not have been state-of-the-art even when they were conducted. Current cRfDs and cADIs do not encompass potential cancer risks, inadequately guard against some endocrine-mediated reproductive and developmental problems, and do not take into account a number of possible genetic, metabolic, and developmental abnormalities. When scientists and regulatory authorities reach consensus on improved pesticide exposure thresholds, they can be readily integrated into the DRI system.

## Conclusions

The DRI system provides a new method to identify both very-low-risk foods and higher-risk pesticides and food-pesticide combinations. DRI system outputs, especially those focused on individual samples by production system and country of origin, can pinpoint sources of high-risk food-pesticide combinations, as well as farming systems and regions routinely associated with reduced pesticide risks. Efforts then can be targeted to better understand the pest management systems that certain farmers have used to reduce reliance on high-risk pesticides. Public and private partnerships can promote wider adoption of those Integrated Pest Management systems commonly found to reduce risks.

The surest way to reduce overall pesticide dietary risk is to identify high-risk food-pest-pesticide combinations, so that research and pest management innovation can accelerate progress toward reducing reliance on pesticides overall, and high-risk pesticides in particular. The DRI system can both highlight where to focus efforts in mitigating pesticide dietary risks, and track progress toward that goal.

## Supplementary information

**Additional file 1.** US-PDP: Chemical Names, Classifications and EPA Toxicity Thresholds (cRfDs, ADIs).

**Additional file 2.** UK-FSA: Chemical Names, Classifications and EPA Toxicity Thresholds (cRfDs, ADIs).

**Additional file 3.** Pesticide Nomenclature Issues; Integrating US-PDP and UK-FSA Data Sets; Serving Sizes; Naming System for Standard DRI Output Reports.

**Additional file 4.** Reconciling Food Names, Food Forms, and Categories in the UK-FSA and US-PDP Pesticide Residue Data Sets.

**Additional file 5.** Food Categories in the US-PDP and UK-FSA Pesticide Residue Data Sets.

**Additional file 6.** US-PDP Foods and Number of Samples Tested, by Year, 1982–2018 (Conventional Markets).

**Additional file 7.** Levels of Parathion Methyl in Domestic Conventional Peaches Tested by the US-PDP in 1996.

**Additional file 8.** Aggregate DRI Values for All Pesticides in Domestic Samples, US-PDP, 1996 [No Banned OCs; Rule of 10 Imposed].

**Additional file 9.** Pesticide Residues and DRI values in Potatoes, US-PDP, 2015 (Conventional, Domestic Samples).

**Additional file 10.** Individual-Sample DRI Values in Apples, US-PDP, 2009 (Conventional, Domestic Samples).

**Additional file 11.** Pesticide Residues and DRI values in Conventionally and Organically Grown Apples, US-PDP, 2009.

## Data Availability

Sources are cited for pesticide residue data, pesticide cRfDs, cPADs, and cADIs, and other food- and pesticide-specific information. Most other data generated or analyzed during this study are included in this published article and its additional information files. Other data are available from the corresponding author on reasonable request.

## Abbreviations

2,4-D: 2,4-dichlorophenoxyacetic acid
ADI: Acceptable daily intake
BW: Body weight
cADI: chronic acceptable daily intake
cRfD: chronic reference dose (EPA)
cRfC: chronic reference concentration
cPAD: chronic population adjusted dose (EPA)
DEEM: Dietary Exposure Evaluation Model
DRI: Dietary risk Index
DRI-M: Positive-sample mean DRI
EC: European Commission
EFSA: European Food Standards Agency
EPA: US Environmental Protection Agency
EU: European Union
EWG: Environmental Working Group
FS-DRI: Food-supply DRI
FQPA: Food Quality Protection Act of 1996
lb.: pound, 454 g
LOD: Limit of detection
LOQ: Limit of quantitation
MRL: Maximum Residue Level
OC: Organochlorine
OP: Organophosphate
oz.: ounce, 28.4 g
ppm: part per million by weight, e.g., mg/kg
PRIMo: Pesticide Residue Intake Model
RfD: Reference dose
RACC: Reference amount customarily consumed per eating occasion
Serv: Serving size
UK-FSA: United Kingdom’s Food Standards Agency
USDA: US Department of Agriculture
US-PDP: USDA’s Pesticide Data Program
