# Supplementary material for: The dietary risk index system: a tool to track pesticide dietary risks
Source: Environ Health. 2020 Oct 14;19:103. doi: 10.1186/s12940-020-00657-z (PMC7557078; doi:10.1186/s12940-020-00657-z)
Supplement: Supplementary file 1 — Additional file 1. US-PDP: Chemical Names, Classifications and EPA Toxicity Thresholds (cRfDs, ADIs). [file 12940_2020_657_MOESM1_ESM.pdf]

Additional File 1 for "US-PDP: Chemical Names, Classifications and EPA Toxicity Thresholds (cRfDs, ADIs)," by Benbrook and Davis

PDP Pesticide Name	Pesticide Type*	Family of Chemistry	cRfD†	EPA FQPA Safety Factor	cPAD	EPA Cancer Class	IARC Cancer Class	QStar	EPA Banned OC	Post-Harvest Fung-icide
1,2,4-Triazole	F	triazole	0.005	1	0.005				☐	☐
1-Naphthol	I	carbamate	0.01	1	0.01	B		0.0227	☐	☐
2,4,5-T	H	unclassified	0.01	1	0.01	D		0	☐	☐
2,4-D	H	alkylchlorophenoxy	0.05	1	0.05	D	2B	0	☐	☐
2,4-DB	H	aryloxyalkanoic acid	0.03	1	0.03	E		0	☐	☐
2,4-DMPF	I	cresol	0.0025	1	0.0025	D		0.0497	☐	☐
2,6-dichlorobenzamide	H	substituted benzene	0.01 †						☐	☐
2,6-DIPN	PGR	biopesticide	0.01 †						☐	☐
3-Hydroxycarbofuran	I	N-methyl Carbamate	0.0002	1	0.0002	E		0	☐	☐
4-Hydroxydiphenylamine	PGR	amine	0.1	1	0.1	E		0	☐	☐
5-Hydroxythiabendazole	F	benzimidazole	0.033	1	0.033	E		0	☐	☐
Abamectin	I	biopesticide	0.0004	1	0.0004	E		0	☐	☐
Acephate	I	organophosphate	0.0012	1	0.0012	C		0	☐	☐
Acequinocyl	I	unclassified	0.027	1	0.027				☐	☐
Acetamiprid	I	neonicotinoid	0.071	1	0.071	E		0	☐	☐
Acetochlor	H	chloroacetamide	0.02	1	0.02	C		0.0327	☐	☐
Acetochlor ethanesulfonic acid	H	chloroacetamide	0.02	1	0.02	C		0.0327	☐	☐
Acetochlor oxanilic acid (OA)	H	chloroacetamide	0.02	1	0.02	C		0.0327	☐	☐
Acibenzolar S methyl	PGR	benzothiadiazole	0.25	1	0.25			0	☐	☐
Alachlor	H	chloroacetamide	0.01	1	0.01	B		0.08	☐	☐
Alachlor ethanesulfonic acid (E	H	chloroacetamide	0.01	1	0.01	B		0.08	☐	☐
Alachlor oxanilic acid (OA)	H	chloroacetamide	0.01	1	0.01	B		0.08	☐	☐
Aldicarb	I	carbamate	0.0013	2	0.00065	E	3	0	☐	☐
Aldicarb sulfone	I	carbamate	0.0013	2	0.00065	E	3	0	☐	☐
Aldicarb sulfoxide	I	carbamate	0.0013	2	0.00065	E	3	0	☐	☐
Aldrin	I	organochlorine	0.000025	1	0.000025	B		17	☑	☐
Allethrin	I	pyrethroid	0.008	1	0.008	C		0	☐	☐

Additional File 1 for "US-PDP: Chemical Names, Classifications and EPA Toxicity Thresholds (cRfDs, ADIs)," by Benbrook and Davis

PDP Pesticide Name	Pesticide Type*	Family of Chemistry	cRfD†	EPA FQPA Safety Factor	cPAD	EPA Cancer Class	IARC Cancer Class	QStar	EPA Banned OC	Post-Harvest Fung-icide
Ametoctradin	F	pyrimidylamine	0.01 †	1					☐	☐
Ametryn	H	triazine	0.072	1	0.072	D		0	☐	☐
Aminomethylphosphonic acid (	H	phosphonoglycine	1.75	1	1.75	E	2A	0	☐	☐
Anilazine	F	triazine	0.0125	1	0.0125	D		0	☐	☐
Atrazine	H	triazine	0.018	10	0.0018	E	3	0	☐	☐
Azadirachtin A	I	biopesticide	0.088			D		0	☐	☐
Azadirachtin B	I	biopesticide	0.088			D		0	☐	☐
Azinphos methyl	I	organophosphate	0.0015	1	0.0015	E		0	☐	☐
Azinphos methyl oxygen analo	I	organophosphate	0.0015	1	0.0015	E		0	☐	☐
Azoxystrobin	F	strobilurin	0.18	1	0.18	E		0	☐	☑
Bendiocarb	I	carbamate	0.0041	1	0.0041	E		0	☐	☐
Benfluralin	H	dinitroaniline	0.005	1	0.005			0	☐	☐
Benomyl	F	benzimidazole	0.05	1	0.05	C		0.0042	☐	☐
Bensulfuron methyl	H	sulfonylurea	0.199	1	0.199	E		0	☐	☐
Bensulide	H	organophosphate	0.005	1	0.005	E		0	☐	☐
Bensulide oxygen analog	H	organophosphate	0.005	1	0.005	E		0	☐	☐
Bentazon	H	benzothiazinone	0.032	10	0.0032	E		0	☐	☐
BHC alpha	I	organochlorine	0.0047	3	0.0016	C	1	0	☑	☐
BHC beta	I	organochlorine	0.0047	3	0.0016	C	1	0	☑	☐
BHC delta	I	organochlorine	0.0047	3	0.0016	C	1	0	☑	☐
Bifenazate	I	hydrazine carboxylate	0.01	1	0.01	E			☐	☐
Bifenthrin	I	pyrethroid	0.013	1	0.013	C		0	☐	☐
Bitertanol	F	triazole	0.0021	1	0.0021	E		0	☐	☐
Boscalid	F	carboxamide	0.218	1	0.218	C			☐	☐
Bromacil	H	uracil	0.0982	1	0.0982	C		0	☐	☐
Bromoxynil	H	hydroxybenzonitrile	0.015	1	0.015	C		0.103	☐	☐
Bupirimate	F	pyrimidinol	0.01 †					0	☐	☐

Additional File 1 for "US-PDP: Chemical Names, Classifications and EPA Toxicity Thresholds (cRfDs, ADIs)," by Benbrook and Davis

PDP Pesticide Name	Pesticide Type*	Family of Chemistry	cRfD†	EPA FQPA Safety Factor	cPAD	EPA Cancer Class	IARC Cancer Class	QStar	EPA Banned OC	Post-Harvest Fung-icide
Buprofezin	I	unclassified	0.0033	1	0.0033	C		0	☐	☐
Captan	F	phthalimide	0.13	1	0.13	B	3	0.0024	☐	☑
Carbaryl	I	carbamate	0.01	1	0.01	B	3	0.00088	☐	☐
Carbendazim (MBC)	F	benzimidazole	0.025	10	0.0025	C		0.00239	☐	☐
Carbofuran	I	N-methyl Carbamate	0.0002	1	0.0002	E		0	☐	☐
Carboxin	F	oxathiin	0.008	1	0.008	E		0	☐	☐
Carfentrazone ethyl	H	triaolinone	0.03	1	0.03	E		0	☐	☐
Chlorantraniliprole	I	anthranilic diamide	1.58	1	1.58	E			☐	☐
Chlordane cis	I	organochlorine	0.0005	1	0.0005	B	2B	1.3	☑	☐
Chlordane trans	I	organochlorine	0.0005	1	0.0005	B	2B	1.3	☑	☐
Chlordanes Total	I	organochlorine	0.0005	1	0.0005	B	2B	1.3	☑	☐
Chlorfenapyr	I	arylpyrrole	0.05	1	0.05	C		0	☐	☐
Chlorimuron ethyl	H	sulfonylurea	0.09	1	0.09	E			☐	☐
Chlorothalonil	F	chloronitrile	0.02	1	0.02	B	2B	0.00766	☐	☐
Chlorpropham	H	carbamate	0.05	1	0.05	E	3	0	☐	☑
Chlorpyrifos	I	organophosphate	0.0003	1	0.0003	E		0	☐	☐
Chlorpyrifos methyl	I	organophosphate	0.001	10	0.0001	E		0	☐	☐
Chlorpyrifos oxygen analog	I	organophosphate	0.0003	1	0.0003	E		0	☐	☐
Chlorsulfuron	H	sulfonylurea	0.05	3	0.0167	E		0	☐	☐
Clethodim sulfone	H	unclassified	0.3	1	0.3			0	☐	☐
Clethodim sulfoxide	H	unclassified	0.3	1	0.3			0	☐	☐
Clodinafop propargyl	H	aryloxyphenoxypropionate	0.0003	10	0.00003	C			☐	☐
Clofentezine	I	tetrazine	0.013	1	0.013	C		0.00376	☐	☐
Clomazone	H	isoxazolidinone	0.84	1	0.84	E		0	☐	☐
Clopyralid	H	pyridine compound	0.15	1	0.15	E		0	☐	☐
Clothianidin	I	neonicotinoid	0.098	1	0.098	E			☐	☐
Coumaphos	I	organophosphate	0.0003	1	0.0003	E		0	☐	☐

Additional File 1 for "US-PDP: Chemical Names, Classifications and EPA Toxicity Thresholds (cRfDs, ADIs)," by Benbrook and Davis

PDP Pesticide Name	Pesticide Type*	Family of Chemistry	cRfD†	EPA FQPA Safety Factor	cPAD	EPA Cancer Class	IARC Cancer Class	QStar	EPA Banned OC	Post-Harvest Fung-icide
Cyanazine	H	triazine	0.0075	1	0.0075	C		1	☐	☐
Cyantraniliprole	I	anthranilic diamide	0.01	1	0.01				☐	☐
Cyazofamid	F	cyanoimidazole	0.948	1	0.948	E			☐	☐
Cycloate	H	thiocarbamate	0.005	1	0.005	E		0	☐	☐
Cyflufenamid	F	amide	0.044	1	0.044				☐	☐
Cyflumetofen	Acaricide	bridged diphenyl	0.17	1	0.17				☐	☐
Cyfluthrin	I	pyrethroid	0.024	1	0.024	E		0	☐	☐
Cyhalothrin, Lambda	I	pyrethroid	0.001	1	0.001	D		0	☐	☐
Cyhalothrin, Lambda epimer R	I	pyrethroid	0.001	1	0.001	D		0	☐	☐
Cyhalothrin, Total	I	pyrethroid	0.001	1	0.001	D		0	☐	☐
Cymoxanil	F	cyanoacetamide oxime	0.0008	10	0.0008	E		0	☐	☐
Cypermethrin	I	pyrethroid	0.06	1	0.06	C		0	☐	☐
Cyphenothrin	I	pyrethroid	0.03						☐	☐
Cyproconazole	F	triazole	0.01	1	0.01	B		0.16	☐	☐
Cyprodinil	F	anilinopyrimidine	0.027	1	0.027	E		0	☐	☐
Cyromazine	I	triazine	0.5	1	0.5	E		0	☐	☐
DCPA	H	benzenedicarboxylic acid	0.01	1	0.01	C		0.00149	☐	☐
DDD (TDE)	I	organochlorine	0.0005	1	0.0005	B		0.24	☑	☐
DDD o,p'	I	organochlorine	0.0005	1	0.0005	B		0.24	☑	☐
DDD p,p'	I	organochlorine	0.0005	1	0.0005	B		0.24	☑	☐
DDE	I	organochlorine	0.0005	1	0.0005	B		0.34	☑	☐
DDE o,p'	I	organochlorine	0.0005	1	0.0005	B		0.34	☑	☐
DDE p,p'	I	organochlorine	0.0005	1	0.0005	B		0.34	☑	☐
DDT	I	organochlorine	0.0005	1	0.0005	B	2A	0.34	☑	☐
DDT o,p'	I	organochlorine	0.0005	1	0.0005	B	2A	0.34	☑	☐
DDT p,p'	I	organochlorine	0.0005	1	0.0005	B	2A	0.34	☑	☐
DEF (Tribufos)	PGR	organophosphate	0.001	10	0.0001	B		0	☐	☐

Additional File 1 for "US-PDP: Chemical Names, Classifications and EPA Toxicity Thresholds (cRfDs, ADIs)," by Benbrook and Davis

PDP Pesticide Name	Pesticide Type*	Family of Chemistry	cRfD†	EPA FQPA Safety Factor	cPAD	EPA Cancer Class	IARC Cancer Class	QStar	EPA Banned OC	Post-Harvest Fung-icide
Deltamethrin (includes parent	I	pyrethroid	0.01	1	0.01	E	3	0	☐	☐
Demeton	I	organophosphate	0.0004	10	0.00004	E		0	☐	☐
Demeton S	I	organophosphate	0.0004	10	0.00004	E		0	☐	☐
Demeton S sulfone	I	organophosphate	0.0004	10	0.00004	E		0	☐	☐
Desethyl atrazine	H	triazine	0.018	10	0.0018	E	3	0	☐	☐
Desethyl desisopropyl atrazine	H	triazine	0.018	10	0.0018	E	3	0	☐	☐
Desisopropyl atrazine	H	triazine	0.018	10	0.0018	E	3	0	☐	☐
Desmedipham	H	carbamate	0.04	1	0.04	E		0	☐	☐
Diazinon	I	organophosphate	0.0002	1	0.0002	E	2A	0	☐	☐
Diazinon oxygen analog	I	organophosphate	0.0002	1	0.0002	E	2A	0	☐	☐
Dicamba	H	benzoic acid	0.45	1	0.45	E		0	☐	☐
Dichlobenil	H	benzonitrile	0.01	1	0.01	C		0	☐	☐
Dichlorprop	H	aryloxyalkanoic acid	0.036	1	0.036	E		0	☐	☐
Dichlorvos (DDVP)	I	organophosphate	0.0005	1	0.0005	C	2B	0.122	☐	☐
Diclofop methyl	H	aryloxyphenoxypropionate	0.0023	1	0.0023	B		0.23	☐	☐
Dicloran	F	chlorophenyl	0.025	10	0.0025	C		0	☐	☐
Dicofol	I	organochlorine	0.0004	1	0.0004	C	3		☐	☐
Dicofol o,p'	I	organochlorine	0.0004	1	0.0004	C	3		☐	☐
Dicofol p,p'	I	organochlorine	0.0004	1	0.0004	C	3		☐	☐
Dicrotophos	I	organophosphate	0.000066	3	0.00002			0	☐	☐
Dieldrin	I	organochlorine	0.00005	1	0.00005	B		0.16	☑	☐
Difenoconazole	F	triazole	0.01	1	0.01	C		0.157	☐	☑
Diflubenzuron	I	benzoylurea	0.02	1	0.02	E		0	☐	☐
Diflufenzopyr	H	semicarbazone	0.26	1	0.26	E			☐	☐
Dimethenamid	H	chloroacetamide	0.05	1	0.05	C		0	☐	☐
Dimethenamid ethanesulfonic	H	chloroacetamide	0.05	1	0.05	C		0	☐	☐
Dimethenamid oxanilic acid (O	H	chloroacetamide	0.05	1	0.05	C		0	☐	☐

Additional File 1 for "US-PDP: Chemical Names, Classifications and EPA Toxicity Thresholds (cRfDs, ADIs)," by Benbrook and Davis

PDP Pesticide Name	Pesticide Type*	Family of Chemistry	cRfD†	EPA FQPA Safety Factor	cPAD	EPA Cancer Class	IARC Cancer Class	QStar	EPA Banned OC	Post-Harvest Fung-icide
Dimethenamid/Dimethenamid	H	chloroacetamide	0.05	1	0.05	C		0	☐	☐
Dimethoate	I	organophosphate	0.0022	1	0.0022	C			☐	☐
Dimethomorph	F	morpholine	0.1	1	0.1	E		0	☐	☐
Dinoseb	H	dinitrophenol	0.01	10	0.001	C			☐	☐
Dinotefuran	I	neonicotinoid	1	1	1	E			☐	☐
Diphenamid	H	alkanamide	0.03	1	0.03	D		0	☐	☐
Diphenylamine (DPA)	PGR	amine	0.1	1	0.1	E		0	☐	☑
Disulfoton	I	organophosphate	0.00013	1	0.00013	E		0	☐	☐
Disulfoton sulfone	I	organophosphate	0.00013	1	0.00013	E		0	☐	☐
Disulfoton sulfone oxygen anal	I	organophosphate	0.00013	1	0.00013	E		0	☐	☐
Disulfoton sulfoxide	I	organophosphate	0.00013	1	0.00013	E		0	☐	☐
Disulfoton sulfoxide oxygen an	I	organophosphate	0.00013	1	0.00013	E		0	☐	☐
Diuron	H	phenylurea	0.001	1	0.001	B		0.0191	☐	☐
Dodine	F	guanidine	0.02	1	0.02			0	☐	☐
Doramectin	I	biopesticide	0.0012	10	0.00012	E			☐	☐
Emamectin benzoate	I	biopesticide	0.000075	1	0.000075	E		0	☐	☐
Endosulfan I	I	organochlorine	0.006	1	0.006	E		0	☐	☐
Endosulfan II	I	organochlorine	0.006	1	0.006	E		0	☐	☐
Endosulfan sulfate	I	organochlorine	0.006	1	0.006	E		0	☐	☐
Endosulfans Total	I	organochlorine	0.006	1	0.006	E		0	☐	☐
Endrin	I	organochlorine	0.00025	1	0.00025	D	3	0	☑	☐
Epoxiconazole	F	triazole	0.02	1	0.02	B		0.0304	☐	☐
EPTC	H	thiocarbamate	0.05	1	0.05	E		0	☐	☐
Esfenvalerate	I	pyrethroid	0.0018	1	0.0018	E		0	☐	☐
Esfenvalerate+Fenvalerate Tot	I	pyrethroid	0.0018	1	0.0018	E		0	☐	☐
Ethephon	PGR	organophosphate	0.06	1	0.06	D		0	☐	☐
Ethion	I	organophosphate	0.0005	1	0.0005	E		0	☐	☐

Additional File 1 for "US-PDP: Chemical Names, Classifications and EPA Toxicity Thresholds (cRfDs, ADIs)," by Benbrook and Davis

PDP Pesticide Name	Pesticide Type*	Family of Chemistry	cRfD†	EPA FQPA Safety Factor	cPAD	EPA Cancer Class	IARC Cancer Class	QStar	EPA Banned OC	Post-Harvest Fung-icide
Ethion mono oxon	I	organophosphate	0.0005	1	0.0005	E		0	☐	☐
Ethofumesate	H	benzofuran	0.3	1	0.3	D		0	☐	☐
Ethoprop	I	organophosphate	0.0014	1	0.0014	B		0.0281	☐	☐
Ethoxyquin	F	quinoline	0.02	1	0.02	D		0.04	☐	☐
Etofenprox	I	pyrethroid	0.05	1	0.05	C		0.0051	☐	☐
Etoxazole	Acaricide	diphenyl oxazoline	0.046	1	0.046	E			☐	☐
Etridiazole	F	aromatic hydrocarbon	0.016	3	0.005	B		0.072	☐	☐
Famoxadone	F	oxazole	0.0014	1	0.0014	E			☐	☐
Fenamidone	F	imidazole	0.0283	1	0.0283	E			☐	☐
Fenamiphos	I	organophosphate	0.0003	10	0.00003	E		0	☐	☐
Fenamiphos sulfone	I	organophosphate	0.0003	10	0.00003	E		0	☐	☐
Fenamiphos sulfoxide	I	organophosphate	0.0003	10	0.00003	E		0	☐	☐
Fenarimol	F	pyrimidine	0.006	1	0.006	E		0	☐	☐
Fenazaquin	I	unknown	0.05	1	0.05			0	☐	☐
Fenbuconazole	F	triazole	0.03	1	0.03	C		0.00359	☐	☐
Fenbutatin oxide	I	organotin	0.017	1	0.017	E		0	☐	☐
Fenhexamid	F	hydroxyanilide	0.17	1	0.17	E		0	☐	☑
Fenobucarb (BPMC)	I	carbamate	0.01 †						☐	☐
Fenpropathrin	I	pyrethroid	0.05	1	0.05	E		0	☐	☐
Fenpyrazamine	F	pyrazole	0.3	1	0.3				☐	☐
Fenpyroximate	I	pyrazole	0.05	1	0.05	E			☐	☐
Fenthion	I	organophosphate	0.00007	1	0.00007	E		0	☐	☐
Fenvalerate	I	pyrethroid	0.0018	1	0.0018	E	3	0	☐	☐
Fipronil	I	pyrazole	0.0002	1	0.0002	C		0	☐	☐
Flonicamid	I	pyridine compound	0.04	1	0.04	C			☐	☐
Fluazifop butyl	H	aryloxyphenoxypropionate	0.0074	1	0.0074	E		0	☐	☐
Fluazinam	F	phenylpyridinamine	0.011	1	0.011	C		0	☐	☐

Additional File 1 for "US-PDP: Chemical Names, Classifications and EPA Toxicity Thresholds (cRfDs, ADIs)," by Benbrook and Davis

PDP Pesticide Name	Pesticide Type*	Family of Chemistry	cRfD†	EPA FQPA Safety Factor	cPAD	EPA Cancer Class	IARC Cancer Class	QStar	EPA Banned OC	Post-Harvest Fung-icide
Flubendiamide	I	benzene-dicarboxamide	0.024	1	0.024				☐	☐
Fludioxonil	F	phenylpyrrole	0.03	1	0.03	D		0	☐	☑
Flufenacet	H	oxyacetamide	0.0017	1	0.0017	E			☐	☐
Flufenacet oxanilic acid (OA)	H	oxyacetamide	0.0017	1	0.0017	E			☐	☐
Flumetsulam	H	triazolepyrimidine	1	1	1	E		0	☐	☐
Fluometuron	H	phenylurea	0.0055	1	0.0055	C	3	0.018	☐	☐
Fluopicolide	F	benzamide	0.2	1	0.2				☐	☑
Fluopyram	F	benzamide, pyramide	0.012	1	0.012				☐	☐
Fluoxastrobin	F	strobilurin	0.015	1	0.015	E			☐	☐
Flupyradifurone	I	butenolide	0.078	1	0.078				☐	☐
Fluridone	H	unclassified	0.15	1	0.15	E		0	☐	☐
Flusilazole	F	triazole	0.002	1	0.002	D		0	☐	☐
Flutolanil	F	oxathiin	0.5	1	0.5	E		0	☐	☐
Flutriafol	F	triazole	0.05	1	0.05				☐	☑
Fluvalinate	I	pyrethroid	0.005	1	0.005	E		0	☐	☐
Fluxapyroxad	F	Pyrazolecarboxamide	0.021	1	0.021	E			☐	☐
Folpet	F	phthalimide	0.09	1	0.09	B		0.00186	☐	☐
Fonofos	I	organophosphate	0.002	1	0.002	E		0	☐	☐
Forchlorfenuron	PGR	phenylurea	0.07	1	0.07	D			☐	☐
Formetanate hydrochloride	I	formamidine	0.00032	1	0.00032	E		0	☐	☐
Glyphosate	H	phosphonoglycine	1.75	1	1.75	E	2A	0	☐	☐
Halosulfuron methyl	F	pyrazole	0.1	1	0.1	E			☐	☐
Heptachlor epoxide	I	organochlorine	0.0005	1	0.0005	B		9.1	☑	☐
Heptachlor epoxide cis	I	organochlorine	0.0005	1	0.0005	B		9.1	☑	☐
Hexachlorobenzene (HCB)	F	organochlorine	0.016	1	0.016	B	2B	1.8	☑	☐
Hexaconazole	F	triazole	0.02	1	0.02	C		0.016	☐	☐
Hexazinone	H	triazinone	0.05	1	0.05	D		0	☐	☐

Additional File 1 for "US-PDP: Chemical Names, Classifications and EPA Toxicity Thresholds (cRfDs, ADIs)," by Benbrook and Davis

PDP Pesticide Name	Pesticide Type*	Family of Chemistry	cRfD†	EPA FQPA Safety Factor	cPAD	EPA Cancer Class	IARC Cancer Class	QStar	EPA Banned OC	Post-Harvest Fung-icide
Hexythiazox	I	carboxamide	0.025	1	0.025	B		0.0222	☐	☐
Hydroprene	I	juvenile hormone mimic	0.1207	1	0.1207	D		0	☐	☐
Hydroxy atrazine	H	triazine	0.018	10	0.0018	E	3	0	☐	☐
Imazalil	F	imidazole	0.025	10	0.0025	B		0.061	☐	☑
Imazamethabenz acid	H	imidazolinone	0.25	1	0.25	E			☐	☐
Imazamethabenz methyl	H	imidazolinone	0.25	1	0.25	D			☐	☐
Imazamox	H	imidazolinone	3	1	3	E		0	☐	☐
Imazapic	H	imidazolinone	1.37	1	1.37	E			☐	☐
Imazapyr	H	imidazolinone	2.5	1	2.5	E		0	☐	☐
Imazaquin	H	imidazolinone racemic mixture	0.25	1	0.25	E		0	☐	☐
Imazethapyr	H	imidazolinone	2.5	1	2.5	E		0	☐	☐
Imidacloprid	I	neonicotinoid	0.057	1	0.057	E		0	☐	☐
Imidacloprid urea	I	neonicotinoid	0.057	1	0.057	E		0	☐	☐
Imiprothrin	I	pyrethroid	0.03						☐	☐
Indaziflam	H	fluoroalkyltriazine	0.02	1	0.02				☐	☐
Indoxacarb	I	oxadiazine	0.02	1	0.02	E		0	☐	☐
Ipconazole	F	triazole	0.015	1	0.015				☐	☐
Iprobenfos (IBP)	F	organophosphate	0.01 †						☐	☐
Iprodione	F	dicarboximide	0.05	10	0.05	B		0.0439	☐	☑
Iprodione metabolite isomer	F	dicarboximide	0.05	10	0.05	B		0.0439	☐	☑
Isoprothiolane	F	unclassified	0.01 †						☐	☐
Isoxaflutole degradate	H	isoxazole	0.2	10	0.02	B		0	☐	☐
Ivermectin	I	biopesticide	0.0012	10	0.00012	E			☐	☐
Kresoxim-methyl	F	strobilurin	0.36	1	0.36	B		0.0029	☐	☐
Lambda cyhalothrin R ester	I	pyrethroid	0.001	1	0.001	D		0	☐	☐
Lambda cyhalothrin S ester	I	pyrethroid	0.001	1	0.001	D		0	☐	☐
Lambda cyhalothrin total	I	pyrethroid	0.001	1	0.001	D		0	☐	☐

Additional File 1 for "US-PDP: Chemical Names, Classifications and EPA Toxicity Thresholds (cRfDs, ADIs)," by Benbrook and Davis

PDP Pesticide Name	Pesticide Type*	Family of Chemistry	cRfD†	EPA FQPA Safety Factor	cPAD	EPA Cancer Class	IARC Cancer Class	QStar	EPA Banned OC	Post-Harvest Fung-icide
Lindane (BHC gamma)	I	organochlorine	0.0047	3	0.0016	C	1	0	☑	☐
Linuron	H	urea	0.0077	1	0.0077	C			☐	☐
Lufenuron	I	benzoylurea	0.01 †					0	☐	☐
Malathion	I	organophosphate	0.071	1	0.071	C	2A	0	☐	☐
Malathion oxygen analog	I	organophosphate	0.071	1	0.071	C	2A	0	☐	☐
Mandipropamid	F	mandelamide	0.05	1	0.05				☐	☐
MCPA	H	aryloxyalkanoic acid	0.0044	1	0.0044	E		0	☐	☐
MCPB	H	aryloxyalkanoic acid	0.0044	10	0.00044	E		0	☐	☐
Mecoprop (MCPP)	H	aryloxyalkanoic acid	0.04	1	0.04			0	☐	☐
Metalaxyl	F	phenylamide	0.0741	1	0.0741	E		0	☐	☐
Metaldehyde	O	cyclo-octane	0.01 †	1	0.01			0	☐	☐
Metconazole	F	triazole	0.04	1	0.04				☐	☐
Methamidophos	I	organophosphate	0.0003	3	0.0001	E		0	☐	☐
Methidathion	I	organophosphate	0.0015	1	0.0015	C		0	☐	☐
Methiocarb	I	carbamate	0.005	1	0.005	D		0	☐	☐
Methomyl	I	carbamate	0.008	1	0.008	E		0	☐	☐
Methoprene	I	juvenile hormone mimic (terpen	0.375	1	0.375	E		0	☐	☐
Methoxychlor olefin	I	organochlorine	0.00501	1	0.00501	D	3	0	☑	☐
Methoxychlor p,p'	I	organochlorine	0.00501	1	0.00501	D	3	0	☑	☐
Methoxychlor Total	I	organochlorine	0.00501	1	0.00501	D	3	0	☑	☐
Methoxyfenozide	I	diacylhydrazine	0.1	1	0.1	E		0	☐	☐
Metolachlor	H	chloroacetamide	0.1	1	0.1	C		0.0092	☐	☐
Metolachlor ethanesulfonic aci	H	chloroacetamide	0.1	1	0.1	C		0.0092	☐	☐
Metolachlor oxanilic acid (OA)	H	chloroacetamide	0.1	1	0.1	C		0.0092	☐	☐
Metribuzin	H	triazinone	0.013	1	0.013	D		0	☐	☐
Metribuzin DA	H	triazinone	0.013	1	0.013	D		0	☐	☐
Metsulfuron methyl	H	sulfonylurea	0.25	1	0.25	E		0	☐	☐

Additional File 1 for "US-PDP: Chemical Names, Classifications and EPA Toxicity Thresholds (cRfDs, ADIs)," by Benbrook and Davis

PDP Pesticide Name	Pesticide Type*	Family of Chemistry	cRfD†	EPA FQPA Safety Factor	cPAD	EPA Cancer Class	IARC Cancer Class	QStar	EPA Banned OC	Post-Harvest Fung-icide
Mevinphos E	I	organophosphate	0.00025	10	0.000025	E		0	☐	☐
Mevinphos Total	I	organophosphate	0.00025	10	0.000025	E		0	☐	☐
Mevinphos Z	I	organophosphate	0.00025	10	0.000025	E		0	☐	☐
MGK-264	I	inorganic compound	0.061	1	0.061	C		0	☐	☐
Mirex	I	organochlorine	0.0007	1	0.0007	B	2B	0	☑	☐
Monocrotophos	I	organophosphate	0.000045	1	0.000045	D		0	☐	☐
Monuron	H	benzoylurea	0.02	1		D	3	0	☐	☐
Myclobutanil	F	triazole	0.025	1	0.025	E		0	☐	☐
Naled	I	organophosphate	0.002	1	0.002	E		0	☐	☐
Napropamide	H	alkanamide	0.12	1	0.12	E		0	☐	☐
Neburon	H	urea	0.15						☐	☐
Nicosulfuron	H	sulfonylurea	1.25	1	1.25	E		0	☐	☐
Nonachlor cis	I	organochlorine	0.0005	1	0.0005	B	2B	1.3	☑	☐
Nonachlor trans	I	organochlorine	0.0005	1	0.0005	B	2B	1.3	☑	☐
Norflurazon	H	pyridazinone	0.015	1	0.015	C		0	☐	☐
Norflurazon desmethyl	H	pyridazinone	0.015	1	0.015	C		0	☐	☐
Novaluron	I	benzoylurea	0.011	1	0.011				☐	☐
Omethoate	I	organophosphate	0.0022	1	0.0022	D			☐	☐
O-Phenylphenol	F	phenol	0.39	1	0.39	B	3	0	☐	☑
Oryzalin	H	dinitroaniline	0.1382	1	0.1382	C		0.13	☐	☐
Ovex	I	bridged diphenyl	0.01 †						☐	☐
Oxadiazon	H	oxidiazole	0.0036	1	0.0036	B		0.0711	☐	☐
Oxadixyl	F	phenylamide	0.109	1	0.109	C		0.053	☐	☐
Oxamyl	I	carbamate	0.001	1	0.001	E		0	☐	☐
Oxamyl oxime	I	carbamate	0.001	1	0.001	E		0	☐	☐
Oxathiapiprolin	F	piperidinyl thiazole isoxazoline	0.01 †	1					☐	☐
Oxychlordane	I	organochlorine	0.0005	1	0.0005	B	2B	1.3	☑	☐

Additional File 1 for "US-PDP: Chemical Names, Classifications and EPA Toxicity Thresholds (cRfDs, ADIs)," by Benbrook and Davis

PDP Pesticide Name	Pesticide Type*	Family of Chemistry	cRfD†	EPA FQPA Safety Factor	cPAD	EPA Cancer Class	IARC Cancer Class	QStar	EPA Banned OC	Post-Harvest Fung-icide
Oxydemeton methyl	I	organophosphate	0.0001	1	0.0001	E		0	☐	☐
Oxydemeton methyl sulfone	I	organophosphate	0.0001	1	0.0001	E		0	☐	☐
Oxyfluorfen	H	diphenyl ether	0.03	1	0.03	C		0.0732	☐	☐
Parathion ethyl	I	organophosphate	0.00003	1	0.00003	C	2B	0	☐	☐
Parathion methyl	I	organophosphate	0.0002	1	0.0002	E	3	0	☐	☐
Parathion methyl oxygen analo	I	organophosphate	0.0002	1	0.0002	E	3	0	☐	☐
Parathion oxygen analog	I	organophosphate	0.00003	1	0.00003	C	2B	0	☐	☐
Penconazole	F	triazole	0.5	1				0	☐	☐
Pendimethalin	H	dinitroaniline	0.3	1	0.3	C		0	☐	☐
Pentachloroaniline (PCA)	F	organochlorine	0.01	10	0.001	C	3	0	☐	☐
Pentachlorobenzene (PCB)	F	organochlorine	0.01	10	0.001	C	3	0	☐	☐
Pentachlorophenyl methyl eth	F	organochlorine	0.005	3	0.0017	B	1	0.129	☐	☐
Pentachlorophenyl methyl sulfi	F	organochlorine	0.01	10	0.001	C	3	0	☐	☐
Penthiopyrad	F	carboxamide	0.27	1	0.27				☐	☐
Permethrin cis	I	pyrethroid	0.25	1	0.25	B	3	0.0096	☐	☐
Permethrin Total	I	pyrethroid	0.25	1	0.25	B	3	0.0096	☐	☐
Permethrin trans	I	pyrethroid	0.25	1	0.25	B	3	0.0096	☐	☐
Phenmedipham	H	bis-carbamate	0.24	1	0.24	D		0	☐	☐
Phenothrin	I	pyrethroid	0.007	1	0.007	E			☐	☐
Phenthoate	I	organophosphate	0.003			D		0	☐	☐
Phorate	I	organophosphate	0.0005	3	0.00017	E		0	☐	☐
Phorate oxygen analog sulfone	I	organophosphate	0.0005	3	0.00017	E		0	☐	☐
Phorate sulfone	I	organophosphate	0.0005	3	0.00017	E		0	☐	☐
Phorate sulfoxide	I	organophosphate	0.0005	3	0.00017	E		0	☐	☐
Phosalone	I	organophosphate	0.002	1	0.002	E		0	☐	☐
Phosmet	I	organophosphate	0.006	1	0.006	C		0	☐	☐
Phosmet oxygen analog	I	organophosphate	0.006	1	0.006	C		0	☐	☐

Additional File 1 for "US-PDP: Chemical Names, Classifications and EPA Toxicity Thresholds (cRfDs, ADIs)," by Benbrook and Davis

PDP Pesticide Name	Pesticide Type*	Family of Chemistry	cRfD†	EPA FQPA Safety Factor	cPAD	EPA Cancer Class	IARC Cancer Class	QStar	EPA Banned OC	Post-Harvest Fung-icide
Phosphamidon	I	organophosphate	0.000167	1	0.000167	C		0	☐	☐
Picloram	H	pyridine compound	0.2	1	0.2	E	3	0	☐	☐
Piperonyl butoxide	I	unclassified	0.155	1	0.155	C	3	0	☐	☐
Pirimicarb	I	carbamate	0.0018	1	0.0018	B		0	☐	☐
Pirimiphos methyl	I	organophosphate	0.0002	3	0.00007	D		0	☐	☑
Prallethrin	I	pyrethroid	0.05	1	0.05	E		0	☐	☐
Prochloraz	F	imidazole	0.009	1	0.009	C		0.15	☐	☐
Procymidone	F	dicarboximide	0.035	1	0.035	B		0.0235	☐	☐
Profenofos	I	organophosphate	0.00005	1	0.00005	E		0	☐	☐
Prometon	H	methoxytriazine	0.05	1	0.05	D		0	☐	☐
Prometryn	H	triazine	0.04	1	0.04	E		0	☐	☐
Pronamide	H	benzamide	0.08	3	0.027	B		0.0259	☐	☐
Propachlor	H	chloroacetamide	0.054	1	0.054	B		0.032	☐	☐
Propachlor oxanilic acid (OA)	H	chloroacetamide	0.054	1	0.054	B		0.032	☐	☐
Propamocarb	I	carbamate	0.12	1	0.12				☐	☐
Propamocarb hydrochloride	I	carbamate	0.12	1	0.12	E		0	☐	☐
Propanil	H	anilide	0.009	1	0.009	C		0	☐	☐
Propargite	I	sulfite ester	0.04	1	0.04	B		0.0033	☐	☐
Propazine	H	triazine	0.018	3	0.006	E		0	☐	☐
Propetamphos	I	organophosphate	0.0005	1	0.0005	E		0	☐	☐
Propiconazole	F	triazole	0.005	1	0.005	C		0	☐	☑
Propiconazole I	F	triazole	0.005	1	0.005	C		0	☐	☑
Propiconazole II	F	triazole	0.005	1	0.005	C		0	☐	☑
Propoxur	I	carbamate	0.006	1	0.006	B		0.0037	☐	☐
Prosulfuron	H	sulfonylurea	0.053	1	0.053	D		0	☐	☐
Pymetrozine	I	pyridine	0.008	1	0.008	B		0.0119	☐	☐
Pyraclostrobin	F	strobilurin	0.034	1	0.034	E			☐	☐

Additional File 1 for "US-PDP: Chemical Names, Classifications and EPA Toxicity Thresholds (cRfDs, ADIs)," by Benbrook and Davis

PDP Pesticide Name	Pesticide Type*	Family of Chemistry	cRfD†	EPA FQPA Safety Factor	cPAD	EPA Cancer Class	IARC Cancer Class	QStar	EPA Banned OC	Post-Harvest Fung-icide
Pyrasulfotole	H	pyrazolone	0.01 †	1	0.01				☐	☐
Pyrethrins	I	biopesticide	0.044	1	0.044	C		0	☐	☐
Pyridaben	I	pyridazinone	0.005	1	0.005	E		0	☐	☐
Pyrimethanil	F	anilinopyrimidine	0.17	1	0.17	C		0	☐	☑
Pyriproxyfen	I	juvenile hormone mimic	0.35	1	0.35	E		0	☐	☐
Quinoxyfen	F	quinoline	0.2	1	0.2	E			☐	☐
Quintozene (PCNB)	F	organochlorine	0.01	10	0.001	C	3	0	☐	☐
Quizalofop ethyl	H	aryloxyphenoxypropionate	0.009	1	0.009	D		0	☐	☐
Resmethrin	I	pyrethroid	0.035	1	0.035	B		0.05621	☐	☐
RH 9129 (fenbuconazole meta	F	triazole	0.03	1	0.03	C		0.00359	☐	☐
RH 9130 (fenbuconazole meta	F	triazole	0.03	1	0.03	C		0.00359	☐	☐
Saflufenacil	H	uracil / amide	0.046	1	0.046				☐	☐
SD-31723	I	organotin	0.017	1	0.017	E		0	☐	☐
SD-33608	I	organotin	0.017	1	0.017	E		0	☐	☐
Sethoxydim sulfoxide	H	unclassified	0.14	1	0.14			0	☐	☐
Siduron	H	urea	0.15	1	0.15	D		0	☐	☐
Simazine	H	triazine	0.018	10	0.0018	E	3	0	☐	☐
Spinetoram	I	spinosyn	0.0249	1	0.0249	E			☐	☐
Spinosad A	I	biopesticide	0.0249	1	0.0259	E		0	☐	☐
Spinosad D	I	biopesticide	0.0249	1	0.0259	E		0	☐	☐
Spinosad Total	I	biopesticide	0.0249	1	0.0259	E		0	☐	☐
Spirodiclofen	I	tetronic acid	0.014	1	0.014	B		0.0149	☐	☐
Spiromesifen	I	tetronic acid	0.022	1	0.022	E			☐	☐
Spiromesifen alcohol	I	tetronic acid	0.022	1	0.022	E			☐	☐
Spiromesifen Total	I	tetronic acid	0.022	1	0.022	E			☐	☐
Spirotetramat	I	tetramic acid	0.05	1	0.05				☐	☐
Spiroxamine	F	morpholine	0.0083	1	0.0083			0	☐	☐

Additional File 1 for "US-PDP: Chemical Names, Classifications and EPA Toxicity Thresholds (cRfDs, ADIs)," by Benbrook and Davis

PDP Pesticide Name	Pesticide Type*	Family of Chemistry	cRfD†	EPA FQPA Safety Factor	cPAD	EPA Cancer Class	IARC Cancer Class	QStar	EPA Banned OC	Post-Harvest Fung-icide
Sulfentrazone	H	aryl triazolinone	0.14	1	0.14	E		0	☐	☐
Sulfometuron methyl	H	sulfonylurea	0.275	1	0.275	D			☐	☐
Sulfoxaflor	I	sulfilimine	0.05	1	0.05				☐	☐
Tebuconazole	F	triazole	0.029	1	0.029	C		0	☐	☐
Tebufenozide	I	diacylhydrazine	0.018	1	0.018	E		0	☐	☐
Tebufenpyrad	I	pyrazole	0.02	1				0	☐	☐
Tebuthiuron	H	urea	0.14	1	0.14	D		0	☐	☐
Tecnazene	F	chlorophenyl	0.01 †			D		0	☐	☐
Tembotrione	H	triketone	0.0004	1	0.0004				☐	☐
Terbacil	H	uracil	0.014	1	0.014	E		0	☐	☐
Terbufos	I	organophosphate	0.00005	1	0.00005	E		0	☐	☐
Terbufos sulfone	I	organophosphate	0.00005	1	0.00005	E		0	☐	☐
Terbufos sulfoxide	I	organophosphate	0.00005	1	0.00005	E		0	☐	☐
Terbuthylazine	H	triazine	0.00035	1	0.00035	D		0	☐	☐
Terbutryn	H	triazine	0.001	1	0.001	C		0	☐	☐
Tetrachlorvinphos	I	organophosphate	0.0423	1	0.0423	B	2B	0.00183	☐	☐
Tetraconazole	F	triazole	0.0073	1	0.0073	B		0.023	☐	☐
Tetradifon	I	bridged diphenyl	0.0006			D		0	☐	☐
Tetrahydrophthalimide (THPI)	F	phthalimide	0.125	1	0.125	E		0	☐	☑
Tetramethrin	I	pyrethroid	0.0071	1	0.0071	C			☐	☐
Thiabendazole	F	benzimidazole	0.033	1	0.033	E		0	☐	☑
Thiacloprid	I	neonicotinoid	0.004	1	0.004	B		0.0406	☐	☐
Thiamethoxam	I	neonicotinoid	0.012	1	0.012	E		0.0377	☐	☐
Thifensulfuron	H	sulfonylurea	0.043	1	0.043	E		0	☐	☐
Thifensulfuron methyl	H	sulfonylurea	0.043	1	0.043	E		0	☐	☐
Thiobencarb	H	thiocarbamate	0.01	1	0.01	D		0	☐	☐
Thiodicarb	I	carbamate	0.03	3	0.011	B		0.0188	☐	☐

Additional File 1 for "US-PDP: Chemical Names, Classifications and EPA Toxicity Thresholds (cRfDs, ADIs)," by Benbrook and Davis

PDP Pesticide Name	Pesticide Type*	Family of Chemistry	cRfD†	EPA FQPA Safety Factor	cPAD	EPA Cancer Class	IARC Cancer Class	QStar	EPA Banned OC	Post-Harvest Fung-icide
Thiophanate methyl	F	benzimidazole	0.0267	1	0.0267	B		0.0116	☐	☐
Thymol	Miticide	phenol	1	1					☐	☐
Toxaphene	I	organochlorine	0.0025	1	0.0025	B	2B	1.1	☑	☐
Tralomethrin	I	pyrethroid	0.0075	1	0.0075			0	☐	☐
Tri Allate	H	thiocarbamate	0.025	1	0.025	C		0.0717	☐	☐
Triadimefon	F	triazole	0.034	1	0.034	C		0	☐	☐
Triadimenol	F	triazole	0.0034	1	0.0034	C		0	☐	☐
Triasulfuron	H	sulfonylurea	0.012	1	0.012	E		0	☐	☐
Triazole acetic acid (TAA)	F	triazole	0.005	1	0.005	C		0	☐	☐
Triazole alanine (TA)	F	triazole	0.005	1	0.005	C		0	☐	☐
Triazophos	I	organophosphate	0.01 †					0	☐	☐
Trichlorfon	I	organophosphate	0.002	10	0.0002	E	3	0	☐	☐
Triclopyr	H	pyridine compound	0.05	1	0.05	D		0	☐	☐
Tricyclazole	F	azole	0.01 †						☐	☐
Trifloxystrobin	F	strobilurin	0.038	1	0.038	E		0	☐	☐
Triflumizole	F	imidazole	0.0117	1	0.0117	E		0	☐	☐
Trifluralin	H	dinitroaniline	0.024	1	0.024	C	3	0.0058	☐	☐
Triforine	F	piperazine	0.025	1	0.025			0	☐	☐
Triticonazole	F	triazole	0.174	1	0.174			0.00856	☐	☐
Uniconazole	PGR	triazole	0.02	1	0.02	C		0	☐	☐
Vinclozolin	F	dicarboximide	0.012	10	0.0012	C		0.0638	☐	☐
Vinclozolin metabolite E	F	dicarboximide	0.012	10	0.0012	C		0.0638	☐	☐
Zoxamide	F	benzamide	0.48	1	0.48				☐	☐

Footnotes:

* Abreviations for Pesticide Types:
  Dis - Dissinfectant F - Fungicide H - Herbicide HS - Herbicide Safener I - Insecticide PGR - Plant Growth Regulator O - Other Pesticide

† Indicates an analyte with no EPA cRfD, unknown cRfD or cADI, or a standard default value of 0.01.
