# Supplementary material for: The dietary risk index system: a tool to track pesticide dietary risks
Source: Environ Health. 2020 Oct 14;19:103. doi: 10.1186/s12940-020-00657-z (PMC7557078; doi:10.1186/s12940-020-00657-z)
Supplement: Supplementary file 2 — Additional file 2. UK-FSA: Chemical Names, Classifications and EPA Toxicity Thresholds (cRfDs, ADIs). [file 12940_2020_657_MOESM2_ESM.pdf]

Additional File 2 for "UK-FSA: Chemical Names, Classifications and EPA Toxicity Thresholds (cRfDs, ADIs)," by Benbrook and Davis

UK-FSA Pesticide Name	PDP Pesticide Name	Pesticide Type*	Family of Chemistry	cRfD†	EPA FQPA Safety	cPAD	EPA Cancer Class	IARC Cancer Class	QStar	EPA Banned OC	Post-Harvest Fung-icide
Acephate	Acephate	I	organophosphate	0.0012	1	0.0012	C		0	☐	☐
Acetamiprid	Acetamiprid	I	neonicotinoid	0.071	1	0.071	E		0	☐	☐
Aldicarb	Aldicarb	I	carbamate	0.0013	2	0.00065	E	3	0	☐	☐
Ametoctradin	Ametoctradin	F	pyrimidylamine	0.01 †	1					☐	☐
Azoxystrobin	Azoxystrobin	F	strobilurin	0.18	1	0.18	E		0	☐	☑
BAC		Dis	biocide	0.1						☐	☐
Bifenthrin	Bifenthrin	I	pyrethroid	0.013	1	0.013	C		0	☐	☐
Boscalid	Boscalid	F	carboxamide	0.218	1	0.218	C			☐	☐
Carbendazim	Carbendazim (MBC)	F	benzimidazole	0.025	10	0.0025	C		0.00239	☐	☐
Chlorantraniliprole	Chlorantraniliprole	I	anthranilic diamide	1.58	1	1.58	E			☐	☐
Chlormequat		PGR	quarternary ammonium	0.05	1	0.05				☐	☐
Chlorothalonil	Chlorothalonil	F	chloronitrile	0.02	1	0.02	B	2B	0.00766	☐	☐
Chlorpropham	Chlorpropham	H	carbamate	0.05	1	0.05	E	3	0	☐	☑
Chlorpropham (potato def.	Chlorpropham	H	carbamate	0.05	1	0.05	E	3	0	☐	☑
Chlorthal-dimethyl	DCPA	H	benzenedicarboxylic acid	0.01	1	0.01	C		0.00149	☐	☐
Cypermethrin	Cypermethrin	I	pyrethroid	0.06	1	0.06	C		0	☐	☐
Cyproconazole	Cyproconazole	F	triazole	0.01	1	0.01	B		0.16	☐	☐
Cyprodinil	Cyprodinil	F	anilinopyrimidine	0.027	1	0.027	E		0	☐	☐
Cyromazine	Cyromazine	I	triazine	0.5	1	0.5	E		0	☐	☐
DDAC		Dis	Antimicrobial	0.1	1	0.1				☐	☐
DDT	DDT	I	organochlorine	0.0005	1	0.0005	B	2A	0.34	☑	☐
Deltamethrin	Deltamethrin (include	I	pyrethroid	0.01	1	0.01	E	3	0	☐	☐
Dichlofluanid	Dichlofluanid	F	sulphamide	0.1					0	☐	☐
Difenoconazole	Difenoconazole	F	triazole	0.01	1	0.01	C		0.157	☐	☑
Dimethoate	Dimethoate	I	organophosphate	0.0022	1	0.0022	C			☐	☐
Dimethomorph	Dimethomorph	F	morpholine	0.1	1	0.1	E		0	☐	☐
Dithiocarbamates		F	disulfur compounds	0.18						☐	☐
Endosulfan	Endosulfans Total	I	organochlorine	0.006	1	0.006	E		0	☐	☐

Additional File 2 for "UK-FSA: Chemical Names, Classifications and EPA Toxicity Thresholds (cRfDs, ADIs)," by Benbrook and Davis

UK-FSA Pesticide Name	PDP Pesticide Name	Pesticide Type*	Family of Chemistry	cRfD†	EPA FQPA Safety	cPAD	EPA Cancer Class	IARC Cancer Class	QStar	EPA Banned OC	Post-Harvest Fung-icide
Epoxiconazole	Epoxiconazole	F	triazole	0.02	1	0.02	B		0.0304	☐	☐
Fenamidone	Fenamidone	F	imidazole	0.0283	1	0.0283	E			☐	☐
Fenhexamid	Fenhexamid	F	hydroxyanilide	0.17	1	0.17	E		0	☐	☑
Fenitrothion	Fenitrothion	I	organophosphate	0.00125	1	0.00125	E		0	☐	☐
Fenpropimorph	Fenpropimorph	F	morpholine	0.032	1	0.032	E			☐	☐
Flonicamid	Flonicamid	I	pyridine compound	0.04	1	0.04	C			☐	☐
Fluazinam	Fluazinam	F	phenylpyridinamine	0.011	1	0.011	C		0	☐	☐
Fludioxonil	Fludioxonil	F	phenylpyrrole	0.03	1	0.03	D		0	☐	☑
Flufenacet	Flufenacet	H	oxyacetamide	0.0017	1	0.0017	E			☐	☐
Flufenoxuron	Flufenoxuron	I	benzoylurea	0.01 †						☐	☐
Fluopicolide	Fluopicolide	F	benzamide	0.2	1	0.2				☐	☑
Fluopyram	Fluopyram	F	benzamide, pyramide	0.012	1	0.012				☐	☐
Fluoxastrobin	Fluoxastrobin	F	strobilurin	0.015	1	0.015	E			☐	☐
Flutolanil	Flutolanil	F	oxathiin	0.5	1	0.5	E		0	☐	☐
Flutriafol	Flutriafol	F	triazole	0.05	1	0.05				☐	☑
Fluxapyroxad	Fluxapyroxad	F	Pyrazolecarboxamide	0.021	1	0.021	E			☐	☐
Folpet	Folpet	F	phthalimide	0.09	1	0.09	B		0.00186	☐	☐
Fosthiazate	Fosthiazate	I	organophosphate	0.000167	1	0.000167			0	☐	☐
Glyphosate	Glyphosate	H	phosphonoglycine	1.75	1	1.75	E	2A	0	☐	☐
Hydrogen Phosphide		O	inorganic	0.01 †						☐	☐
Imazalil	Imazalil	F	imidazole	0.025	10	0.0025	B		0.061	☐	☑
Imidacloprid	Imidacloprid	I	neonicotinoid	0.057	1	0.057	E		0	☐	☐
Indoxacarb	Indoxacarb	I	oxadiazine	0.02	1	0.02	E		0	☐	☐
Inorganic Bromide		Fumigant	inorganic	1					0	☐	☐
Iprodione	Iprodione	F	dicarboximide	0.05	10	0.05	B		0.0439	☐	☑
Isopyrazam	Isopyrazam	F	Carboxamide	0.055	1	0.055				☐	☐
Kresoxim-methyl	Kresoxim-methyl	F	strobilurin	0.36	1	0.36	B		0.0029	☐	☐
Lambda-Cyhalothrin	Lambda cyhalothrin to	I	pyrethroid	0.001	1	0.001	D		0	☐	☐

Additional File 2 for "UK-FSA: Chemical Names, Classifications and EPA Toxicity Thresholds (cRfDs, ADIs)," by Benbrook and Davis

UK-FSA Pesticide Name	PDP Pesticide Name	Pesticide Type*	Family of Chemistry	cRfD†	EPA FQPA Safety	cPAD	EPA Cancer Class	IARC Cancer Class	QStar	EPA Banned OC	Post-Harvest Fung-icide
Lufenuron	Lufenuron	I	benzoylurea	0.01 †					0	☐	☐
Malathion	Malathion	I	organophosphate	0.071	1	0.071	C	2A	0	☐	☐
Maleic Hydrazide		PGR	pyridazine	0.25	1	0.25	E		0	☐	☐
Mandipropamid	Mandipropamid	F	mandelamide	0.05	1	0.05				☐	☐
MCPA	MCPA	H	aryloxyalkanoic acid	0.0044	1	0.0044	E		0	☐	☐
Mepiquat		PGR	quarternary ammonium	0.584	1	0.584	E		0	☐	☐
Metalaxyl	Metalaxyl	F	phenylamide	0.0741	1	0.0741	E		0	☐	☐
Methamidophos	Methamidophos	I	organophosphate	0.0003	3	0.0001	E		0	☐	☐
Methomyl	Methomyl	I	carbamate	0.008	1	0.008	E		0	☐	☐
Omethoate	Omethoate	I	organophosphate	0.0022	1	0.0022	D			☐	☐
Oxadixyl	Oxadixyl	F	phenylamide	0.109	1	0.109	C		0.053	☐	☐
Oxamyl	Oxamyl	I	carbamate	0.001	1	0.001	E		0	☐	☐
Pencycuron	Pencycuron	F	phenylurea	0.01 †						☐	☐
Pendimethalin	Pendimethalin	H	dinitroaniline	0.3	1	0.3	C		0	☐	☐
Pirimicarb	Pirimicarb	I	carbamate	0.0018	1	0.0018	B		0	☐	☐
Pirimiphos-Methyl	Pirimiphos methyl	I	organophosphate	0.0002	3	0.00007	D		0	☐	☑
Prochloraz	Prochloraz	F	imidazole	0.009	1	0.009	C		0.15	☐	☐
Procymidone	Procymidone	F	dicarboximide	0.035	1	0.035	B		0.0235	☐	☐
Propamocarb	Propamocarb hydrochl	I	carbamate	0.12	1	0.12				☐	☐
Propargite	Propargite	I	sulfite ester	0.04	1	0.04	B		0.0033	☐	☐
Propyzamide		H	benzamide	0.084	3	0.028	B		0.0154	☐	☐
Pymetrozine	Pymetrozine	I	pyridine	0.008	1	0.008	B		0.0119	☐	☐
Pyraclostrobin	Pyraclostrobin	F	strobilurin	0.034	1	0.034	E			☐	☐
Pyrethrins	Pyrethrins	I	biopesticide	0.044	1	0.044	C		0	☐	☐
Pyrimethanil	Pyrimethanil	F	anilinopyrimidine	0.17	1	0.17	C		0	☐	☑
Quintozene	Quintozene (PCNB)	F	organochlorine	0.01	10	0.001	C	3	0	☐	☐
Spinosad	Spinosad Total	I	biopesticide	0.0249	1	0.0259	E		0	☐	☐
Spirotetramat	Spirotetramat	I	tetramic acid	0.05	1	0.05				☐	☐

Additional File 2 for "UK-FSA: Chemical Names, Classifications and EPA Toxicity Thresholds (cRfDs, ADIs)," by Benbrook and Davis

UK-FSA Pesticide Name	PDP Pesticide Name	Pesticide Type*	Family of Chemistry	cRfD†	EPA FQPA Safety	cPAD	EPA Cancer Class	IARC Cancer Class	QStar	EPA Banned OC	Post-Harvest Fung-icide
Tebuconazole	Tebuconazole	F	triazole	0.029	1	0.029	C		0	☐	☐
Tebufenozide	Tebufenozide	I	diacylhydrazine	0.018	1	0.018	E		0	☐	☐
Tecnazene	Tecnazene	F	chlorophenyl	0.01 †			D		0	☐	☐
Tefluthrin	Tefluthrin	I	pyrethroid	0.005	1	0.005			0	☐	☐
Thiabendazole	Thiabendazole	F	benzimidazole	0.033	1	0.033	E		0	☐	☑
Thiacloprid	Thiacloprid	I	neonicotinoid	0.004	1	0.004	B		0.0406	☐	☐
Thiamethoxam	Thiamethoxam	I	neonicotinoid	0.012	1	0.012	E		0.0377	☐	☐
Thiamethoxam (sum)	Thiamethoxam	I	neonicotinoid	0.012	1	0.012	E		0.0377	☐	☐
Tolclofos-methyl	Tolclofos methyl	F	organophosphate	0.1667	1	0.1667			0	☐	☑
Tolylfluanid	Tolyfluanid	F	sulphamide	0.026	1	0.026			0.00159	☐	☐
Triadimenol	Triadimenol	F	triazole	0.0034	1	0.0034	C		0	☐	☐
Tricyclazole	Tricyclazole	F	azole	0.01 †						☐	☐
Trifloxystrobin	Trifloxystrobin	F	strobilurin	0.038	1	0.038	E		0	☐	☐
Vinclozolin	Vinclozolin	F	dicarboximide	0.012	10	0.0012	C		0.0638	☐	☐

Footnotes:

* Abreviations for Pesticide Types:

Dis - Dissinfectant F - Fungicide H - Herbicide HS - Herbicide Safener I - Insecticide PGR - Plant Growth Regulator O - Other Pesticide

† Indicates an analyte with no EPA cRfD, unknown cRfD or cADI, or a standard default value of 0.01.
