# Supplementary material for: The dietary risk index system: a tool to track pesticide dietary risks
Source: Environ Health. 2020 Oct 14;19:103. doi: 10.1186/s12940-020-00657-z (PMC7557078; doi:10.1186/s12940-020-00657-z)
Supplement: Supplementary file 3 — Additional file 3. Pesticide Nomenclature Issues; Integrating US-PDP and UK-FSA Data Sets; Serving Sizes; Naming System for Standard DRI Output Reports. [file 12940_2020_657_MOESM3_ESM.pdf]

Pesticide Nomenclature Issues; Integrating US-PDP and UK-FSA Data Sets; Serving Sizes; Naming System for Standard DRI Output Reports

1. Pesticide Nomenclature, Metabolites, and Moieties

Substantial pesticide and food-related nomenclature issues must be resolved between the US-PDP and UK-FSA data sets in order to compare residue and risk levels between them. These include:

- Changes in the names of pesticide active ingredients (e.g., "1 Naphthol" versus "1-Naphthol," or "endosulfan" versus "total endosulfan");
- Differences in the number and names of metabolites or related moieties over time and across the two data sets (e.g., the US-PDP reports residues of fenvalerate and esfenvalerate, while UK-FSA reports fenvalerate only); and
- Marked differences in some food names, food forms, and food categories (e.g., summer squash in the US-PDP versus courgettes in the UK-FSA). (See Additional Files 2 and 3.)

Sometimes the US-PDP has reported residues of individual pesticide parent compounds, isomers, and metabolites, as well as amounts of the "total" active ingredient. Fortunately, the PDP has never reported both for the same samples, which could lead to double counting. It has in some years, however, used for some samples of a given food-pesticide combination a "total" measure of residues (encompassing the parent, metabolites, and isomers), while for the other samples reporting analyte-specific results (and no "total" amount). This difference arises from variations in the analytical methods used in different labs conducting residue testing for the US-PDP.

For example, the 2008 US-PDP reports residues of endosulfan I, endosulfan II, and endosulfan sulfate. The DRI system calculates DRI values for each of these three forms of endosulfan, and adds them together to form the total endosulfan DRI for a given food tested that year.

There are also issues in linking pesticide names over time and across the US-PDP and UK-FSA data sets. For example, the US-PDP has listed the insecticide cyhalothrin as "Cyhalothrin, Lambda" or "Lambda Cyhalothrin," while the UK-FSA lists this active ingredient as "lambda-cyhalothrin." The US-PDP lists a variety of isomers including Lambda Cyhalothrin S ester, and in some years, "Cyhalothrin, Total." The UK-FSA does not report amounts for lambda-cyhalothrin metabolites.

2. Resolving Differences in US-PDP and UK-FSA Residue Testing Programs

2.1 The USDA's Pesticide Data Program

In 2018, the USDA republished its entire PDP database, wherein nomenclature issues and inconsistent use of pesticide codes and names were standardized from 1992 to the present. This alleviated nearly all pesticide-nomenclature-related concerns arising from prior reports.

A remaining complication is that some parent compounds can also be a metabolite of another parent compound, e.g., omethoate is a metabolite of dimethoate, as well as a stand-alone active ingredient. In such cases, it is not possible to determine whether a residue of omethoate reported in a given food resulted from an application of an omethoate or dimethoate insecticide.

To address this problem as well as is possible, the DRI system generates its most detailed, food-pesticide-combination reports in two ways: (a) based on each pesticide's parent compound and any isomers or metabolites tested for and reported by the US-PDP, or (b) based on a pesticide-specific "risk group." Such a "risk group" aggregates all the analytes reported that can arise from an application of a specific active ingredient. The risk-group method assures that all relevant residues, and their associated DRI contributions, are included in a food-pesticide's DRI value.

For each analyte, the DRI value is calculated based on its reported residue levels and, where possible, its specific toxicity threshold. However, regulatory agencies usually assume that isomers and breakdown products are comparably toxic to the parent compound. Hence they use the same cRfD to quantify the contribution of isomers and metabolites to dietary exposures and risk. We do essentially the same in the DRI system.

2.2 The UK-FSA Pesticide Residue Testing System

The UK-FSA primarily tests foods for residues of parent chemicals. In 2012 it implemented a new nomenclature standard, in which "(sum)" or "(partial sum)" after the parent pesticide name signifies whether all, or only some, isomers or metabolites were Included.

In integrating UK-FSA residue data into the DRI system, we usually assume that all pesticide names followed by "(sum)" include the parent chemical and any of its known metabolites or isomers. Such residue levels are equivalent to US-PDP "total" residue levels.

There are a few exceptions to the assumption stated above. For some active ingredients for which the UK-FSA or US-PDP report "sum" or "total" residue amounts, it is not always clear what analytes are included. These uncertainties and our assumptions are noted in the following table:

UK-FSA Pesticide Names of Concern	Assumption/Resolution
**Fenvalerate (sum)** – includes esfenvalerate?	Yes. Fenvalerate includes 4 isomers (SS, RR, RS, SR), where SS is esfenvalerate. Esfenvalerate is registered as a separate active ingredient in the US.
**Phosmet (partial sum)** – what analytes are included in the partial sum?	Phosmet and Phosmet (partial sum) are reported separately until further information is obtained.
**Spirotetramat (partial sum)** – what analytes are included in the partial sum?	Spirotetramat and Spirotetramat (partial sum) are reported separately until notified otherwise.
**DDT (sum)** – Does this include DDE?	Yes, DDT (sum) is same as DDT, and includes DDE.
**Emamectin vs. Emamectin benzoate** – They have the same FSA pesticide code, however, they are different chemicals. They have different CAS numbers and are reported separately by PDP. FSA reports emamectin benzoate in 2015, then switches to emamectin from 2016 to present. There is no cRfD for emamectin on the EPA website. There is no aRfD established for emamectin B1, however, there is one for emamectin benzoate.	Emamectin is reported separately from emamectin benzoate. Supporting EFSA documentation at: https://efsa.onlinelibrary.wiley.com/doi/pdf/10.2903/j.efsa.2019.5748
**Folpet vs. Captan** – Does Captan (sum) include Folpet; and, Folpet (sum) include Captan?<br>• Captan,<br>• Captan (sum) – 2017-2018 only,<br>• Captan and Folpet,<br>• Folpet (sum) – 2017-2018 only,<br>• Folpet.	Currently assumed to be the following:<br>Captan<br>Captan<br>Captan and Folpet<br>Folpet<br>Folpet
**Fluazifop vs. Fluazifop-p-butyl**. Fluazifop-p-butyl is a different chemical compound than Fluazifop. Fluazifop and Fluazifop-p are the same. What does Fluazifop-p (partial sum) include?	Reported Fluazifop-p (partial sum) separately from Fluazifop.
**Phorate (partial sum)** – what analytes are included in the partial sum?	Phorate and Phorate (partial sum) are reported separately until notified otherwise.
**Thiamethoxam vs. Thiamethoxam (sum)** – what is the difference?	Reported separately, until notified otherwise.
**2,4-D (partial sum)** – what analytes are included in the partial sum?	2,4,D and 2,4,D (partial sum) are reported separately until notified otherwise.

2.3 Linking US-PDP and UK-FSA Residue Data

Linking UK-FSA pesticide active ingredient names with US-PDP names is straight forward, since the UK-FSA tests primarily for parent chemicals. Both systems have an assigned code from either the EPA or FSA. These codes are matched by pesticide name or by CAS numbers. Parent pesticides listed in the UK-FSA data set are linked to the parent pesticide or risk group used in the PDP data set. Pesticides in the UK-FSA system that are reported as partial sums will not link directly to the PDP system, since it is unclear which analytes are included in a partial sum.

The DRI system establishes a standardized name for each pesticide in both the US-PDP and UK-FSA systems that links it to other databases developed and maintained by Benbrook Consulting Services. These databases encompass a variety of active-ingredient classifications and information:

- EPA CAS and registration numbers;
- Type of pesticide, chemistry family, and mode of action;
- Post-harvest fungicide [Yes/No];
- Banned organochlorine [Yes/No];
- Registrant and regulatory history information;
- Physical and chemical properties;
- Toxicity data, safety factors, and regulatory thresholds (cRfDs, aRfDs, cPADs);
- Pesticide use data in the US from the USDA; and
- Oncogenic risk classifications and Q*s (if set).

For US-PDP pesticides no longer registered by the EPA, we use the most recent value of the active ingredient's cRfD or cPAD.

For pesticides tested by the UK-FSA but not by EPA, we use the cADI set by EFSA or other regulatory authority.

Some pesticides do not require dietary risk analysis by the EPA. Typically these are biopesticides that pose no, or very low, dietary risks because of low toxicity or absence of residues. Examples include insect pheromones, some microbial pesticides, horticultural oils, and mineral-based pesticides such as copper-based fungicides. For such products lacking an EPA-set cRfD or cPAD, we assign a default cRfD of 0.01 mg/kg body weight/day. This precautionary default value minimizes the chances of underestimating potential dietary risks if residues of these pesticides are reported in the future.

Current cRfDs, cPADs, and other ADIs are shown in Additional Files 1 and 2. These values

are routinely updated and accessible on Hygeia Analytics at https://hygeia-analytics.com/pesticides/dietary-risks/dietary-risk-index/

3. Serving Sizes Used in the DRI System

All DRI system outputs assume (1) a BW of 16 kg—the median for children near age 4 years, and (2) serving sizes nearly always equal to about 2/3 of FDA's RACCs for the general US population. RACCs were developed from national food consumption surveys and serve as guides for serving sizes listed on food labels (21 CFR 101.12 for most foods and 9 CFR 317.312 for some animal products) (1, 2). For a few US foods, we used available RACCs called NLEA serving sizes in USDA's National Nutrient Database for Standard Reference Legacy Release (SR Legacy), now at USDA's FoodData Central (3). NLEA refers to the Nutrition Labeling and Education Act of 1990 (4).

NLEA serving sizes were especially useful for lettuce types, because the best matching RACC for lettuce is for "vegetable salad" (100 g), whereas USDA lists NLEA serving sizes for 3 types of lettuce—89 g for crisphead lettuce and 85 g for Romaine and red leaf types. We used 87 g as a typical, average NLEA serving size for lettuce.

RACC and NLEA serving sizes are reported usually in metric weight (g) and volume measures (mL). To convert them to approximate US household units (e.g., tablespoons, cups and oz.), we used the weight-volume and weight-portion relationships in USDA's SR Legacy database. Table 1 shows some common and a few unusual examples of these conversions.

For most commodities tested by the US-PDP or UK-FSA, we used the following steps to determine serving sizes: look up the RACC or NLEA RACC serving size for that commodity, select a matching USDA food, select a USDA serving size with weight or volume near the RACC or NLEA serving size, record USDA's serving-weight relationship in both household units and g, and multiply by 2/3 to estimate the serving size for a 16-kg child. For lettuce which has multiple common forms, we used a composite of crisphead, Romaine, and red leaf types, and a composite of USDA's weight-volume relationships for these types. For rare foods with no RACC, we used a common commercial serving size (e.g., dry tea).

Table 1: Serving Sizes of Selected Foods Tested by US PDP or UK FSA

US-PDP or UK-FSA commodity, edible portion, raw or minimally processed	RACC or NLEA serving size	Matching USDA SR Legacy database serving		USDA serving size with weight near the RACC		Child serving size (2/3 of USDA serving with weight near RACC)	
		NDB Number	Description	(household units)	(g)	(household units)	Serv (g)
Almonds	30 g	12061	Unroasted	1 oz.	28	2/3 oz.	19
Apple Juice	240 mL	09016	Bottled or canned, unsweetened	1 cup	248	2/3 cup	165
Apples	140 g	09003	With skin, small, 2.75-inch diameter	1 apple	149	2/3 apple	99
Asparagus	85 g	11011	Raw, large spear (~8 inch)	4 spears	80	2.7 spears	53
Bagel	110 g	18001	Bagel, plain, 3.5- to 4-inch diameter	1 bagel	105	2/3 bagel	70
Honey	1 tbsp	19296	Honey	1 tbsp	21	2 tsp	14
Lettuce	87 g[a]	Comp[b]	Composite of chopped and shredded	1.7 cup	88	1.1 cup	59
Poultry muscle, raw	114 g[c]	--	--	4 oz.	114	2.7 oz.	76
Tea, dry	3 g[d]	--	--	1 teabag	3	2/3 teabag	2

a. NLEA RACC (composite of 89, 85, and 85 g for crisphead, Romaine, and red leaf, respectively)

b. Composite of NDB no. 11252, 11251, and 11257

c. 114 g (4 oz.) raw corresponds to about 3 oz. cooked

d. No RACC. Common teabag content in UK. Needed for UK-FSA data only

4. Naming System for Standard DRI Output Reports

The DRI system generates multiple versions of standard reports 2–8 according to combinations of the country-of-origin of the samples tested, market claims, and inclusion criteria (Rule of 10, OCs in or out). There are seven country-of-origin options for each report, denoted by a matrix lettering system, where the first group of letter(s) describes the following:

A – All positive samples, regardless of country of origin;

I – All imported samples;

C – Imported samples disaggregated by country of origin;

D – US Domestic samples;

UK – UK domestic samples (grown or processed);

EC – Imported samples into the UK from other EC countries; and

NEC – Imported samples into the UK from non-EC countries.

The multiple versions of standard reports 2–8 can also include 3 market-claim options, denoted by the last letter of the report-naming matrix, as follows (see Table 3):

A – All samples, regardless of market claim;

C – Samples lacking a market claim, and referred to as "Conventionally Grown"; and

O – Samples labeled as organically grown.

Table 3: Naming Matrix for DRI System Reports

		**All Market Claims**	**Conventional**	**Organic**
		A	C	O
All Samples	A	AA	AC	AO
Imports Combined	I	IA	IC	IO
Imports by Country	C	CA	CC	CO
Domestic (US standpoint)	D	DA	DC	DO
Domestic (UK standpoint)	UK	UKA	UKC	UKO
Imports to UK from EC countries	EC	ECA	ECC	ECO
Imports to UK from Non-EC countries	NEC	NECA	NECC	NECO

The DRI system standard-output reports use the following file names:

[Report number]_[DRI System]_[Dataset]_[Report description], where:

Report number = 2–8 standard report number

DRI System = PDP or FSA

Data set = [origin] + [market claim]

Report description = brief report description

For example, Report 3 food-pesticide DRI Values for samples of US domestically grown, conventional food tested by the US-PDP would be labeled: "Report 3_PDP_DC_DRI_by_Food."

As detailed above, there are 12 residue data sets from which US-PDP DRI-system reports can be generated and 18 data sets for the UK-FSA DRI-system. Each residue data set includes a specific set of samples that match the selection criteria.

DRI output reports run from a few pages, to > 900 pages for the two most detailed reports (Reports 3 and 4). US-PDP Report 3_PDP_AA_DRI_by_Food, for example, reports DRI values by food, based on all samples from 1992 to 2018, and is 529 pages long, while Report 4__PDP_AA_DRI_by_Chemical reports DRI values for all foods and all samples in which a given pesticide or metabolite was found. It runs 1090 pages. For this reason,

access to DRI system analytical outputs is provided via interactive look-up tables accessible online (5).

References

1. Code of Federal Regulations. 9 CFR Section 317.312: Reference amounts customarily consumed per eating occasion. In: United States Department of Agriculture, editor. 2011.
2. Code of Federal Regulations. 21 CFR Section 101.12: Reference amounts customarily consumed per eating occasion. In: Department of Health and Human Services, editor. 2019.
3. Department of Agriculture (US), Agricultural Research Service. FoodData Central. Available from: https://fdc.nal.usda.gov/.
4. Nutrition Labeling and Education Act of 1990, Pub. L. No. Public Law 101-535(November 8, 1990, 1990).
5. Benbrook Consulting Services. Dietary Risk Index. Available from: https://hygeia-analytics.com/pesticides/dietary-risks/dietary-risk-index/.
