# Supplementary material for: The dietary risk index system: a tool to track pesticide dietary risks
Source: Environ Health. 2020 Oct 14;19:103. doi: 10.1186/s12940-020-00657-z (PMC7557078; doi:10.1186/s12940-020-00657-z)
Supplement: Supplementary file 4 — Additional file 4. Reconciling Food Names, Food Forms, and Categories in the UK-FSA and US-PDP Pesticide Residue Data Sets. [file 12940_2020_657_MOESM4_ESM.pdf]

Reconciling Discordant Food Names, Food Forms, and Categories in the UK-FSA and US-PDP Pesticide Residue Datasets

A variety of food-nomenclature issues arise in the course of integrating the pesticide residue data reported by the UK-FSA into the DRI system. Two major clusters of issues had to be worked through: (1) changes in food names, categories, and food forms tested by the UK-FSA over time, and (2) differences in a particular year in the food names and food forms tested by the US-PDP in contrast to the generally comparable food names and food forms tested by the UK-FSA. Herein, we describe how the UK-FSA food nomenclature scheme was modified to harmonize food names and categories over time and across datasets for use within the DRI system.

Standardizing UK-Food Standards Agency (FSA) Food and Sub-Food Names

The UK-FSA reports food-pesticide residue testing results by sample in Brand Name Annex reports (BNA). From 1999 through 2015, the UK-FSA released its detailed food-pesticide residue testing results in tabular formats in hard-copy, quarterly reports. For these years, the data were extracted from pdf files by Benbrook Consulting Services, and placed within a relational database for analyses within the DRI system. Beginning with the 2016 quarterly reports, the UK-FSA has made the raw data available in Excel spreadsheets, greatly facilitating movement of the data into a relational database like the one supporting applications of the DRI system.

Over the years the foods tested by the UK-FSA changed in many ways, creating multiple food-related nomenclature issues within the UK-FSA dataset. Herein we explain how we reconciled these differences in order to consistently classify foods within the DRI system over time.

Nomenclature Guidelines

- Many, but not all raw or fresh foods are expressed in the plural form. For example, apples, mangos and beans. Food names that encompass many varieties of a specific food are used in their singular form, such as, kale, spinach, and broccoli. This approach is adhered to in order to match, to the full extent possible, food names in the UK-FSA and US-PDP datasets.
- ‘None’ was replaced as ‘Not Specified’, in the Sub-Food field.
- The British English spelling of words is generally adopted.
- Ethnic foods with African, Indian or Hindi names are translated to British English.

Table 1 lists a sample of foods by their British English and American English names:

**Table 1. Translation of Food Names from British English to American English**	
**British English**	**American English**
Abergines	Eggplant
Courgettes	Zucchini
Crisps	Potato Chips
Chips	French Fries
Beetroot	Beets
Vine leaves	Grape leaves

Minced	Burger
Tinned	Canned
Maize	Corn
Soya	Soy
Rocket	Arugula
Langoustines	Lobster
Methi	Fenugreek
Marrow, is a white-fleshed green-skinned gourd, which is eaten as a vegetable. Whereas, the American English marrow, is the substance inside bones.	

Additional Steps to Standardize Food Nomenclature and Categories

- Foods that were reported by the UK-FSA as two, closely related foods, such as 'Peaches and Nectarines' are separated into their individual foods. Examples include:
  - Peaches and Nectarines,
  - Lemons and Limes,
  - Berries and small fruits,
  - Raisins, Currants and Sultanas,
  - Sunflower and Pumpkin seeds,
  - Sardines and Mackerel,
  - Herbal infusion and tea,
  - Goats milk and Ewes milk,
  - Ewe/Goats Cheese,
  - Frozen Fruits and Smoothies,
  - Oats and Rye
  - Trout and Salmon
- Several dozen foods that are tinned (canned) are reported as a separate food item, distinct from their raw or fresh food form.
- Foods that are processed are reported as a separate food from their raw, fresh, or unprocessed counterparts. Examples include:
  - Beef (processed)
  - Chicken (processed)
  - Pork (processed)
  - Pork and Beef (processed)
  - Potatoes (processed)
  - Tomatoes (processed)
  - Turkey (processed)
- Smoked Fish is reported as a separate food item, and in addition to "Fish."

Reorganizing Foods and Sub-Foods

Foods and Sub-Foods as reported by the UK-FSA were not consistent from year to year (e.g., Savoury Breads are reported as a Sub-Food of Specialty Breads in some, but not all years). In general, the UK-FSA testing program is far more granular than the US-PDP, and reports results for multiple varieties of common foods like beans, cheeses, and tree-fruits (e.g., green apples, red apples, cooking apples). In

contrast, the US-PDP typically reports results for fresh apples, a food name that encompasses all different variety of apples in the raw or fresh form.

Several common foods tested by the UK-FSA are reorganized in the DRI system to maintain consistency and comparability, to the full extent possible, over time. The following major UK-FSA food categories have been used in the DRI system:

- Cheese
- Fish
- Beans with Pods
- Processed Meats
- Berries and Frozen Fruits

Tables (2-6) illustrate the reorganization of these five major foods. The "Brand Name Annex" (BNA) food and sub-food names are used in the UK-FSA dataset. The "Standardized" food and sub-food names are used to assure that roughly the same foods tested in the early 2000s by the UK-FSA are compared to the same foods tested in the 2010s. The column "Future Refinements" in Table 2 provides examples of the complex challenges that lie ahead in fully reconciling differences in food nomenclature in the UK-FSA and US-PDP datasets.

Table 2. Cheese

Brand Name Annex		Standardized		Future Refinements
**Food**	**Sub-Food**	**Food**	**Sub-Food**	
Cheese	Hard Cheese	Cheese	Hard Cheese	All the hard cheeses (typically Cheddar, Edam and Gouda) were not separated out into Sub-Foods in 2014 and 2017. However, the variety of cheese is listed in the description field and could be separated out to be consistent with other years.
Cheese	None	Cheese	Not Specified	In 2006, the BNA reports did not separate out the variety of cheeses into a Sub-Food. However, the variety of cheese is listed in the description field and could be separated out to be consistent with other years.
Cheese	Other	Cheese	Other	Other cheeses (typically Mozzarella and Feta) were not separated out into Sub-Foods in 2010. However, the variety of cheese is listed in the description field and could be separated out to be consistent with other years.
Cheese	Processed Cheese	Cheese	Processed Cheese	'Processed Cheese' are soft spreadable cheeses in 2016, that could be relabeled as Spreadable Cheese to be consistent with 2009 and 2013.
Cheese	Sheep	Cheese	Sheep	

Cheese	Soft Cheese	Cheese	Soft Cheese	All the soft cheeses were not separated out into Sub-Foods in 2009, 2012, 2015, 2016 and 2019. However, the variety of cheese is listed in the description field and could be separated out to be consistent with other years.
Cheese	Spreadable cheese	Cheese	Spreadable cheese	2016 only.
Cheese (hard)	Cheddar	Cheese	Cheddar	
Cheese (hard)	Double Gloucester	Cheese	Double Gloucester	
Cheese (hard)	Edam	Cheese	Edam	
Cheese (hard)	Gouda	Cheese	Gouda	
Cheese (hard)	Hard Cheese	Cheese	Hard Cheese	All the hard cheeses (typically Cheddar, Edam and Gouda) were not separated out into Sub-Foods in 2014 and 2017. However, the variety of cheese is listed in the description field and could be separated out to be consistent with other years.
Cheese (soft)	Brie	Cheese	Brie	
Cheese (soft)	Camembert	Cheese	Camembert	
Cheese (soft)	Cottage Cheese	Cheese	Cottage Cheese	
Cheese (soft)	Cream Cheese	Cheese	Cream Cheese	
Cheese (soft)	Dolcelatte	Cheese	Dolcelatte	
Cheese (soft)	Feta	Cheese	Feta	
Cheese (soft)	Mozzarella	Cheese	Mozzarella	
Cheese (soft)	Other	Cheese	Other	
Cheese (soft)	Ricotta	Cheese	Ricotta	
Cheese (soft)	Soft Cheese	Cheese	Soft Cheese	All the soft cheeses were not separated out into Sub-Foods in 2009, 2012 and 2016. However, the variety of cheese is listed in the description field and could be separated out to be consistent with other years.
Cheese Imported	None	Cheese	Imported	All imported cheeses are hard cheeses that were not separated out into Sub-Foods in 2002. However, the variety of cheese is listed in the description field and could be separated out to be consistent with other years.

Cheese Mature	None	Cheese	Mature	All Mature cheeses are aged Cheddar cheeses that were not separated out into Sub-Foods in 2004. However, these could be reassigned with a Sub-Food of Cheddar to be consistent with other years.
Cheese Mild	None	Cheese	Mild	All Mild cheeses are typically the milder Cheddar cheeses that were not separated out into Sub-Foods in 2004. However, these could be reassigned with a Sub-Food of Cheddar, to be consistent with other years.
Ewe/Goats Cheese	Ewes Cheese	Cheese	Ewe	
Ewe/Goats Cheese	Goats Cheese	Cheese	Goat	
Processed Cheese	Soft Cheese	Cheese	Soft Cheese	All the soft cheeses were not separated out into Sub-Foods in 2009, 2012 and 2016. However, the variety of cheese is listed in the description field and could be separated out to be consistent with other years.
Processed Cheese	Spreadable cheese	Cheese	Spreadable cheese	2016 only.
UK Cheese	None	Cheese	Cheddar	

All Fish samples have been reorganized by species, where Fish is the Food and the species is the Sub-Food.

Table 3. Fish

Brand Name Annex		Standardized		Notes
**Food**	**Sub-Food**	**Food**	**Sub-Food**	
Deep Water Fish	None	Fish	Antarctic Ice Fish	
Deep Water Fish	None	Fish	Cod	
Deep Water Fish	None	Fish	Coley	
Deep Water Fish	None	Fish	Haddock	
Deep Water Fish	None	Fish	Halibut	
Deep Water Fish	None	Fish	Plaice	
Deep Water Fish	None	Fish	Whiting	
Farmed Fish	Salmon	Fish	Salmon	The variety field designates that these samples are Farmed.
Farmed Fish	Trout	Fish	Trout	The variety field designates that these samples are Farmed.

Fish (fresh water)	Basa	Fish	Basa	Basa is the same as River Cobbler.
Fish (fresh water)	Carp	Fish	Carp	
Fish (fresh water)	Puta	Fish	Puta	
Fish (fresh water)	River Cobbler	Fish	River Cobbler	
Fish (fresh water)	Salmon	Fish	Salmon	
Fish (fresh water)	Tilapia	Fish	Tilapia	
Fish (fresh water)	Trout	Fish	Trout	
Fish (oily)	Herring	Fish	Herring	
Fish (oily)	Mackerel	Fish	Mackerel	
Fish (oily)	Monkfish	Fish	Monkfish	
Fish (oily)	Pangasius	Fish	Pangasius	
Fish (oily)	Salmon	Fish	Salmon	
Fish (oily)	Sprats	Fish	Sprats	
Fish (oily)	Tilapia	Fish	Tilapia	
Fish (oily)	Trout	Fish	Trout	
Fish (oily)	Tuna	Fish	Tuna	
Fish (oily)	Whitebait	Fish	Whitebait	
Fish (white)	Basa	Fish	Basa	Basa is the same as River Cobbler.
Fish (white)	Cod	Fish	Cod	
Fish (white)	Coley	Fish	Coley	
Fish (white)	Haddock	Fish	Haddock	
Fish (white)	Hake	Fish	Hake	
Fish (white)	Plaice	Fish	Plaice	
Fish (white)	Pollock	Fish	Pollock	
Fish (white)	Sea bass	Fish	Sea bass	
Fish (white)	Sea bream	Fish	Sea bream	
Fish (white)	Sole	Fish	Sole	
Fish Predator	Swordfish	Fish	Swordfish	
Fish Predator	Tuna	Fish	Tuna	
Fresh Salmon	None	Fish	Salmon	
Oily Fish	Herring	Fish	Herring	
Oily fish	Mackerel	Fish	Mackerel	
Oily Fish	None	Fish	Herring	* See footnote
Oily Fish	None	Fish	Mackerel	* See footnote
Oily Fish	None	Fish	Not Specified	
Oily fish	Salmon	Fish	Salmon	
Oily Fish	Sprats	Fish	Sprats	
Oily Fish	Swordfish	Fish	Swordfish	
Oily fish	Trout	Fish	Trout	
Oily Fish	Tuna	Fish	Tuna	
Oily Fish	Whitebait	Fish	Whitebait	
Salmon	None	Fish	Salmon	

Sea Fish	Cod	Fish	Cod	
Sea Fish	Coley	Fish	Coley	
Sea Fish	Dover sole	Fish	Dover sole	
Sea Fish	Escolar	Fish	Escolar	
Sea Fish	Haddock	Fish	Haddock	
Sea Fish	Hake	Fish	Hake	
Sea Fish	Herrings	Fish	Herring	
Sea Fish	Lemon Sole	Fish	Sole	
Sea Fish	Mackerel	Fish	Mackerel	
Sea Fish	Monk fish	Fish	Monkfish	
Sea Fish	None	Fish	Cod	* See footnote
Sea Fish	None	Fish	Coley	* See footnote
Sea Fish	None	Fish	Dab	* See footnote
Sea Fish	None	Fish	Dover Sole	* See footnote
Sea Fish	None	Fish	Grey Mullet	* See footnote
Sea Fish	None	Fish	Guild Herd Bream	* See footnote
Sea Fish	None	Fish	Haddock	* See footnote
Sea Fish	None	Fish	Halibut	* See footnote
Sea Fish	None	Fish	Marlin	* See footnote
Sea Fish	None	Fish	Monkfish	* See footnote
Sea Fish	None	Fish	Mullet	* See footnote
Sea Fish	None	Fish	Other White Fish	* See footnote
Sea Fish	None	Fish	Plaice	* See footnote
Sea Fish	None	Fish	Red Snapper	* See footnote
Sea Fish	None	Fish	Red Tilapia	* See footnote
Sea Fish	None	Fish	Sea Bass	* See footnote
Sea Fish	None	Fish	Sea Bream	* See footnote
Sea Fish	None	Fish	Sole	* See footnote
Sea Fish	None	Fish	Swordfish	* See footnote
Sea Fish	None	Fish	Tilapia	* See footnote
Sea Fish	None	Fish	Tuna	* See footnote
Sea Fish	None	Fish	Turbot	* See footnote
Sea Fish	None	Fish	Whiting	* See footnote
Sea Fish	None	Fish	Yellow Snapper	* See footnote
Sea Fish	Other	Fish	Other White Fish	
Sea Fish	Plaice	Fish	Plaice	
Sea Fish	Pollack	Fish	Pollock	
Sea Fish	Pollock	Fish	Pollock	
Sea Fish	Red Snapper	Fish	Red Snapper	
Sea Fish	River Cobbler	Fish	River Cobbler	
Sea Fish	Sea Bass	Fish	Sea Bass	
Sea Fish	Sea Bream	Fish	Sea Bream	
Sea Fish	Swordfish	Fish	Swordfish	

Sea Fish	Tilapia	Fish	Tilapia	
Sea Fish	White Fish	Fish	Other White Fish	
Trout	None	Fish	Trout	
Trout and Salmon	Salmon	Fish	Salmon	
Trout and Salmon	Trout	Fish	Trout	
White Fish	Cod	Fish	Cod	
White Fish	Coley	Fish	Coley	
White Fish	Haddock	Fish	Haddock	
White Fish	Halibut	Fish	Halibut	
White Fish	None	Fish	Cod	* See footnote
White Fish	None	Fish	Coley	* See footnote
White Fish	None	Fish	Haddock	* See footnote
White Fish	None	Fish	Plaice	* See footnote
White Fish	None	Fish	Whiting	* See footnote
White Fish	Other	Fish	Basa	* See footnote, Basa is the same as River Cobbler.
White Fish	Other	Fish	Hake	* See footnote
White Fish	Other	Fish	Other White Fish	* See footnote
White Fish	Other	Fish	Pollock	* See footnote
White Fish	Other	Fish	River Cobbler	* See footnote
White Fish	Other	Fish	Sole	* See footnote
White Fish	Plaice	Fish	Plaice	
White Fish	River Cobbler	Fish	River Cobbler	
White Fish	Sea bass	Fish	Sea Bass	
White Fish	Sea bream	Fish	Sea Bream	
White Fish	Sole	Fish	Sole	
White Fish	Whiting	Fish	Whiting	
Footnote				
* Species of fish is documented in the foods description of the BNA reports and is specified as the Sub-Food.				

Beans have been reorganized by variety. Each variety is categorized as a green bean or other bean. Green beans include varieties such as runner, French, dwarf, Bobi and string beans. Other beans include varieties such as fresh black-eyed beans, edamame, uri, hyacinth, chowli and mungra beans. Specialty beans are varieties that are not commonly grown in Europe and include types such as yard long beans, lima beans, guar, and valore beans. Since the country of origin is specified, this is a duplication of information and not necessary, and therefore, are separated out by Sub-Food as a green bean, dried bean, or other bean. Some beans, such as, black-eyed beans, are sampled as a dry bean or fresh bean and are split out as ‘Beans (dried)’ or ‘Beans with Pods - Other’, respectively. The individual variety of beans for each sample are retained in the Variety field in the database.

**Table 4. Beans**				
**Brand Name Annex**		**Standardized**		**Notes**
**Food**	**Sub-Food**	**Food**	**Sub-Food**	

Broad Beans	Fresh	Beans with Pods	Green Beans	Fresh is retained in Form field
Broad Beans	Frozen	Beans with Pods	Green Beans	Frozen is retained in Form field
Green Beans	Green Beans	Beans with Pods	Green Beans	
Green Beans	None	Beans with Pods	Green Beans	
Green Beans	Specialty Beans	Beans with Pods	Green Beans	
Green Beans	Specialty Beans	Beans with Pods	Other Bean	
Pulses	Beans	Beans without Pods	Dried Beans	
Pulses	None	Beans (tinned)	Baked Beans	
Pulses	None	Beans (tinned)	Butter Beans	
Pulses	None	Beans (tinned)	Cannellini	
Pulses	None	Beans (tinned)	Haricot	
Pulses	None	Beans (tinned)	Kidney	
Pulses	None	Beans (tinned)	Pinto	
Pulses	None	Beans without Pods	Dried Beans	
Specialty Beans (dried)	None	Beans without Pods	Dried Beans	
Specialty Beans	None	Beans with Pods	Green Beans	
Specialty Beans	None	Beans with Pods	Other Bean	
Specialty Beans (dried)	None	Beans without Pods	Dried Beans	

Processed meats are reorganized by the primary animal source. They include pork, turkey, chicken and beef.

Table 5. Processed Meats

Brand Name Annex		Standardized	
**Food**	**Sub-Food**	**Food**	**Sub-Food**
Bacon	Bacon	Pork (processed)	Bacon
Bacon	Gammon	Pork (processed)	Gammon
Bacon	None	Pork (processed)	Bacon
Breaded Turkey Products	None	Turkey (processed)	Breaded Turkey Products
Chicken (processed)	None	Chicken (processed)	Not Specified
Chicken Nuggets/ Breaded Chicken	None	Chicken (processed)	Nuggets/Breaded Chicken
Chinese Canned Pork Products	None	Pork (processed)	Chinese Canned Pork Products
Cooked and Cured Pork Meats	Chorizo	Pork (processed)	Chorizo
Cooked and Cured Pork Meats	Garlic Sausage	Pork (processed)	Sausage
Cooked and Cured Pork Meats	Ham	Pork (processed)	Ham
Cooked and Cured Pork Meats	Other	Pork (processed)	Other
Cooked and Cured Pork Meats	Roast Pork	Pork (processed)	Roast Pork
Cooked and Cured Pork Meats	Salami	Pork (processed)	Salami
Cooked Meat	Beef	Beef (processed)	Cooked Meat

Cooked Meat	Chicken	Chicken (processed)	Cooked Meat
Cooked Meat	Ham	Pork (processed)	Ham
Cooked Meat	Pork	Pork (processed)	Roast Pork
Cooked Meat	Turkey	Turkey (processed)	Cooked Meat
Cooked Meats	Beef	Beef (processed)	Cooked Meat
Cooked Meats	Chicken	Chicken (processed)	Cooked Meat
Cooked Meats	Ham	Pork (processed)	Ham
Cooked Meats	Pork	Pork (processed)	Roast Pork
Cooked Meats	Turkey	Turkey (processed)	Cooked Meat
Cooked or Cured Pork Meats	Chorizo	Pork (processed)	Chorizo
Cooked or Cured Pork Meats	Garlic Sausage	Pork (processed)	Sausage
Cooked or Cured Pork Meats	Ham	Pork (processed)	Ham
Cooked or Cured Pork Meats	Other	Pork (processed)	Other
Cooked or Cured Pork Meats	Roast Pork	Pork (processed)	Roast Pork
Cooked or Cured Pork Meats	Salami	Pork (processed)	Salami
Ham	None	Pork (processed)	Ham
Pork and Gammon	Gammon	Pork (processed)	Gammon
Pork Products (Processed)	Bacon	Pork (processed)	Bacon
Pork Products (Processed)	Gammon	Pork (processed)	Gammon
Pork Products (processed)	Meatballs	Pork (processed)	Meatballs
Pork Products (processed)	Sausage Meat	Pork (processed)	Sausage
Pork Products (Processed)	Sausages	Pork (processed)	Sausage
Poultry (processed)	Chicken	Chicken (processed)	Not Specified
Poultry (Processed)	Chicken	Chicken (processed)	Not Specified
Poultry (processed)	Turkey	Turkey (processed)	Breaded Turkey Products
Poultry (Processed)	Turkey	Turkey (processed)	Breaded Turkey Products
Processed Pork	Bacon	Pork (processed)	Bacon
Processed Pork	Gammon	Pork (processed)	Gammon
Processed Pork	Sausage Meat	Pork (processed)	Sausage
Processed Pork	Sausages	Pork (processed)	Sausage
Sausages	Beef	Beef (processed)	Sausage
Sausages	None	Beef (processed)	Sausage
Sausages	None	Chicken (processed)	Sausage
Sausages	None	Pork (processed)	Sausage
Sausages	None	Pork and Beef (processed)	Sausage
Sausages	Pork	Pork (processed)	Sausage
Sausages	Pork and Beef	Pork and Beef (processed)	Sausage

All fresh and frozen berries are reorganized as one food, with the individual berry as the Sub-Food. Fresh and frozen berries are kept separate to correspond with the BNA reports. Strawberries are listed as a

separate Food from berries with respect to the original BNA reports, and to correspond with US-PDP data.

Table 6. Berries and Frozen Fruits			
Brand Name Annex		Standardized	
Food	Sub-Food	Food	Sub-Food
Berries	Fresh: Blackberries	Berries	Blackberries (fresh)
Berries	Fresh: Blackberries	Berries	Blackberries (fresh)
Berries	Fresh: Blueberries	Berries	Blueberries (fresh)
Berries	Fresh: Blueberries	Berries	Blueberries (fresh)
Berries	Fresh: Gooseberries	Berries	Gooseberries (fresh)
Berries	Frozen: Blackberries	Berries	Blackberries (frozen)
Berries	Frozen: Blackberries	Berries	Blackberries (frozen)
Berries	Frozen: Blueberries	Berries	Blueberries (frozen)
Berries	Frozen: Blueberries	Berries	Blueberries (frozen)
Berries and Small Fruits	Blackberries	Berries	Blackberries
Berries and Small Fruits	Blackcurrants	Berries	Blackcurrants
Berries and Small Fruits	Blueberries	Berries	Blueberries
Berries and Small Fruits	Gooseberries	Berries	Gooseberries
Berries and Small Fruits	Redcurrants	Berries	Redcurrants
Blackberries	None	Berries	Blackberries
Blueberries	None	Berries	Blueberries
Currants	Black Currants	Berries	Blackcurrants
Currants	Red Currants	Berries	Redcurrants
Frozen Fruits	Blackberries	Berries	Blackberries (frozen)
Frozen Fruits	Smoothie Mixes	Frozen Mixed Fruits	Smoothie Mixes
Frozen Fruits and Smoothie Mixes	Frozen Blackberries	Berries	Blackberries (frozen)
Frozen Fruits and Smoothie Mixes	Frozen Blueberries	Berries	Blueberries (frozen)
Frozen Fruits and Smoothie Mixes	Frozen Raspberries	Berries	Raspberries (frozen)
Frozen Fruits and Smoothie Mixes	Frozen Strawberries	Strawberries	Frozen
Prepfruit	Blueberry	Berries	Blueberry (prepared)
Raspberries	Fresh	Berries	Raspberries (fresh)
Raspberries	Frozen	Berries	Raspberries (frozen)
Raspberries	None	Berries	Raspberries
Raspberries and Blackberries	Blackberries (fresh)	Berries	Blackberries (fresh)
Raspberries and Blackberries	Raspberries (fresh)	Berries	Raspberries (fresh)
Raspberries and Blackberries	Raspberries (frozen)	Berries	Raspberries (frozen)
Raspberry	None	Berries	Raspberries
Raspberry	Not Specified	Berries	Raspberries
Specialty Fruit	Gooseberry	Berries	Gooseberries

Summer Berries	Blackberries	Berries	Blackberries
Summer Berries	Blueberries	Berries	Blueberries
Summer Berries	Gooseberries	Berries	Gooseberries
Summer Berries	Raspberries	Berries	Raspberries
Summer Berries	Red Currants	Berries	Redcurrants
