# Supplementary material for: The dietary risk index system: a tool to track pesticide dietary risks
Source: Environ Health. 2020 Oct 14;19:103. doi: 10.1186/s12940-020-00657-z (PMC7557078; doi:10.1186/s12940-020-00657-z)
Supplement: Supplementary file 5 — Additional file 5. Food Categories in the US-PDP and UK-FSA Pesticide Residue Data Sets. [file 12940_2020_657_MOESM5_ESM.pdf]

Categories of Foods in the US-PDP and UK-FSA Pesticide Residue Datasets

The foods tested by the UK-FSA and US-PDP vary greatly. Each year, the US-PDP tests 10-20 different raw, fresh, whole, and processed foods that account for a significant share of average, daily caloric intake by pregnant women, infants, and children. To the full extent possible, foods are tested by the US-PDP "as eaten." Accordingly, a banana or orange are tested after peeling, and a potato is tested after boiling.

During a given program year, the US-PDP sampling protocol calls for the selection of several hundred to 700 samples of a specific food, including domestic production and imports (if any), and conventional and organic foods.

The US-PDP strives to select the samples of food in terms of country of origin and production system (i.e. conventional versus organic) roughly proportional to market share. So, if imported fresh cherries account for about 30% of annual US sales, the US-PDP would strive to assure that imported samples account for about 30% of total samples.

The UK-FSA tests far fewer samples per food than the US-PDP in an annual cycle, but tests a much greater array of fresh, processed, and multi- ingredient food products. The UK-FSA places heavy focus on foods imported into the UK that have been found in the past to contain possibly worrisome pesticide residues.

In order to compare results from the same foods tested by the UK-FSA and US-PDP in a given year, or trends over time for the same food, a methodology was developed to reconcile, to the extent possible, the foods, food categories, and food forms tested by the two programs. The DRI System merges the food commodities from the UK-FSA and US-PDP into one comprehensive food list, and then reconciles differences to the full extent possible(see Additional File 2).

For example, samples of 'Green Beans' from the PDP system link to samples of 'Beans with Pods - Green Beans' from the UK-FSA system using a UK-FSA Food Coding Schema developed by Benbrook Consulting Services that is now part of the DRI system.

The PDP publishes relatively brief and generic descriptions of each food and food forms that they test. Based on these descriptions, the corresponding food samples from the UK-FSA system are selected to link together, using the Food Coding Schema.

Defining Food Forms

Both programs test several foods in multiple forms -- fresh, dried, canned, frozen, purees, sauces, and juices. In the case of beef, pork, lamb, and chicken, the two programs periodically test muscle tissue (meat), fat, and certain organ meats.

Raw/Fresh

Raw or fresh food forms are essentially unchanged from their form at harvest, and have only had minimal washing prior to storage and consumption. No processing has been done that changes the quality or nutrient content of the food.

Derivative Food Forms

Many food products are derived from raw commodities via food processes that change the 'nature' of the food. For example, wheat may be ground into the form of whole-grain flour, a process through which no other ingredients are added or subtracted. A cucumber can be pickled, and a grape dried to become a raisin. The following table lists several common food manufacturing processes and the foods that they apply to:

Process	Applied to
Seasoning	Meat, milk
Curing	Meat, milk
Ripening	Meat, milk
Preserving by salt	Vegetables, meat, fish, seafood
Preserving by sugars	Fruit, chestnuts. Candying is included
Drying (dehydration)	Meat, fish, seafood, milk, egg, vegetables, legumes, fruit, spices, herbs and teas.
Marinating	Meat, fish, seafood
Pickling	Vegetables
Fermentation	Milk, vegetables, meat
Canning/jarring	Vegetable, fruit, meat, fish, seafood
Smoking	Meat, fish, seafood
Flour	Grains, nuts
Minced/burger	Meat, fish

Multi-ingredient Composite Food Forms

Food products with multiple ingredients are created via recipes of ingredients and a distinct set of steps, e.g. cooking or baking. Following the example of wheat, when mixed with yeast, egg, salt, etc., and baked it becomes bread, a multi-ingredient composite food product.

The following processes have been identified by the European Food Standards Agency (EFSA) as the most relevant to specific food groups:

Process	Applied to	Leading to
Winemaking	Grape and other fruits	Wine and similar
Beer production	Malted cereals	Beer
Cheesemaking	Milk	Cheese
Churning	Milk, cream	Butter
Oil production	Oil fruits and oilseeds	Oils and fats
Grain milling	Cereal grains and similar	Flours, groats, etc.
Grain milling—starch production	Cereal grains and similar starch sources	Starch
Sugar production	Sugar plants	Sugar
Pulping/mashing	Fruit, vegetables	Fruit/vegetable purée
Cooking in water	Eggs	Hardened egg products
Frying	Potatoes	Fries, chips and other potato snacks

Baking	Cereal milling products and other ingredients	Bakery products
Roasting (baking with fat)	Potatoes	Roasted potatoes
Toasting	Coffee beans	Toasted coffee
Caramelization/browning	Sugar	Caramel
Mixing	Creation of compound food	Recipes (composite food)
Filling	Filled branches of unfilled bases	Filled pasta, filled bakery products
Extrusion	Cereals, snack production	Extruded snacks and breakfast cereals
Flaking	Cereals	Cereal flakes
Flattening/rolling	Cereals	Rolled cereals
Puffing/expanding	Cereals	Puffed cereals
Gelling	Milk, water	Desserts, jellies
Distillation	Fruits or sugary/starchy sources	Alcohol and related products
Separation (in liquid phase)	Milk	Cream
Brewing/infusion	Infusion materials, cacao, coffee etc.	Hot drinks
Juicing	Fruit, vegetables	Fruit and vegetable juices and nectars
Extraction	Meat, fish, yeast, infusion materials	Extracts
Concentration/evaporation	Milk, juices from vegetable and fruit	Concentrates

The Food Coding Schema

In the DRI system, foods and food products are organized into a hierarchy of food categories, groups, individual foods or products, varieties, and food forms. Each tier in the hierarchy adds more specific information, and has a 1- or 2-digit code that is assigned to each item in that tier. The first tier -- the food category -- has a 1-digit code. All other tiers have 2-digit codes. Combining the codes for all 5 tiers produces a unique identifier for each individual food or product taking into account the variety and the form of the food.

For example, a fresh ambrosia apple would have a unique identifier of 505011101.

Where:
Food Category = Fruits (5)
Food Group = Pome (05)
Food = Apple (01)
Variety = Ambrosia (11)
Form = Fresh (01)

Whereas, apple sauce, made with a generic or unknown variety of apple would have a code of 505010005.

Where:
Food Category = Fruits (5)
Food Group = Pome (05)
Food = Apple (01)
Variety = Not Specified (00)
Form = Puree/Sauce (05)

This coding system was developed in order to harmonize the nomenclature governing foods tested by the US-PDP in a given year and over time, with the foods tested by the UK-FSA in a given year and over time.

Food Categories

Food categories are at the top tier of the DRI system food-code hierarchy. The categories used in the DRI system consist of meats, animal bi-products (dairy and eggs), nuts & oils, grains, fruits, vegetables, miscellaneous foods, such as beverages and sugar, and composite (multi-ingredient) foods. These categories are similar to those used in other food coding schemas developed by government food agencies.

Food Groups

The second tier divides each of the food categories into food groups, as shown in the below table.

Food Categories (Tier 1) and Food Groups (Tier 2) as Designated in the DRI System Food Coding Schema

1st Tier - Category	Dairy & Eggs	Meats	Nuts & Oils	Grains	Fruits	Vegetables	Miscellaneous	Composite
2nd Tier - Groups	Milk	Beef	Nuts	Barley	Berries	Cruciferous	Alcoholic Beverages	Cereals
	Creams	Fish	Seeds	Buckwheat	Citrus	Edible Stem	Coffee	Chocolate
	Butter	Wild Game	Nut Butters	Bulgar Wheat	Exotic	Exotic	Cotton	Dressings
	Cheeses	Lamb	Cooking Oils	Corn/Maize	Melons	Fruiting Vegetables	Herbs	Fruits & Vegetables
	Yogurt	Ox	Fish Oils	Millet	Pome	Leafy Greens	Soft Drinks	Grain Products
	Eggs	Pig		Oats	Stone	Mushrooms	Spices	Infant Food
		Poultry		Rice		Pulses	Sugars	Jams & Jellies
		Shellfish		Rye		Roots/Tubers/Bulbs	Teas	Meat Products
		Organ Meats		Sorghum		Squashes	Tobacco	Mixed Foods
		Animal Fats		Soy Grain			Water	Ordinary Bread
				Spelt				Speciality Bread
				Wheat				

Food/Commodity

Tier 3 of the hierarchy is the Food/Commodity level. The foods are typically listed in their raw form, or the primary source of a food product. At this level, the form of a food as typically found at a retail market is included, possibly along with other food forms.

Some foods may be sold at market as fresh, canned, juiced, dried, and pureed. In these instances and where the food form was not specified, additional food form codes are necessary. A code '00' is used where the form of the food reported by US-PDP or UK-FSA was not specified and cannot be assumed to be fresh.

Variety/Product

Tier 4 indicates the variety, or a derivative of the raw food. A classic example would be for tomatoes. There are cherry, roma and beefsteak varieties. In addition, there are derivative products, such as, ketchup, paste, and sauces that have been processed, yet have few if any added ingredients.

Full Food Code	Variety (digits 6-7)	Food	Variety/Derivative
6041301	01	Tomatoes	Cherry

6041303	03	Tomatoes	Roma
6041304	04	Tomatoes	Beefsteak
6041311	11	Tomatoes	Ketchup
6041312	12	Tomatoes	Passate
6041313	13	Tomatoes	Paste

Food Form

Tier 5 indicates the form that the food or food product is found at market. They apply to raw, derivative, and composite food products. The following is a list of forms found in either US-PDP or UK-FSA foods. The Form code is the last 2 digits of the code, and can apply to any of the upper tiers in the hierarchy.

Form Code	Form
00	Not Specified
01	Fresh/raw
02	Frozen
03	Canned/Tinned
04	Processed
05	Puree/Sauce
06	Dried
07	Cooked/Roasted
08	Smoked
09	Burger/Minced
10	Juice
11	Flour
12	Sprouted
13	Fermented
14	Cured
15	Uncured
16	Potted (herbs)
17	Liquified (nut milks)
18	Sliced
19	Water Added
20	Infant Food
21	Concentrate

In keeping with the tomato example, tomatoes (60413) has the following food forms available within the DRI.

Full Food Code	Form (last 2 digits)	Food	Form
604130000	00	Tomatoes	Not Specified
604130001	0	Tomatoes	Fresh
604130003	03	Tomatoes	Canned/Tinned
604130005	05	Tomatoes	Sauce/Puree
604130010	10	Tomatoes	Juiced

The full DRI Food Coding Schema is accessible on Hygeia Analytics at https://hygeia-analytics.com/wp-content/uploads/2020/05/DRI-Full-Table-of-Food-Coding-Schema.pdf
