# Supplementary material for: The dietary risk index system: a tool to track pesticide dietary risks
Source: Environ Health. 2020 Oct 14;19:103. doi: 10.1186/s12940-020-00657-z (PMC7557078; doi:10.1186/s12940-020-00657-z)
Supplement: Supplementary file 6 — Additional file 6. US-PDP Foods and Number of Samples Tested, by Year, 1982–2018 (Conventional Markets). [file 12940_2020_657_MOESM6_ESM.pdf]

Additional File 6 for "The Dietary Risk Index System: A Tool to Track Pesticide Dietary Risks," by Benbrook and Davis

Commodities Tested by PDP over time in Domestic Conventional Markets, Number of Samples per Commodity, Number of Foods Tested and Total Number of Samples per Year.

Commodities Tested by PDP	1992	1993	1994	1995	1996	1997	1998	1999	2000	2001	2002	2003	2004	2005	2006	2007	2008	2009	2010	2011	2012	2013	2014	2015	2016	2017	2018
Almonds																344	171										
Apple Juice					162	515	426				508					222	196				181	141					
Apple Sauce											347				711										166	489	
Apples	538	630	670	659	502			360	180	692	505	685	679	714				672	670				169	648	483		
Apples-Single Servings								1354																			
Asparagus											211	175					29	119	86							12	83
Asparagus, Canned												339															
Avocado																					90						
Baby Food - Applesauce																					345	331					
Baby Food - Carrots																					690						
Baby Food - Green Beans																			180	563							
Baby Food - Peaches																					225						
Baby Food - Pears																			94	308							
Baby Food - Peas																					367	350					
Baby Food - Sweet Potato																			132	488							
Bananas		6																									
Barley											706	434															
Beef Adipose										284	298							289									
Beef Liver										304	310																
Beef Muscle										302	307							289									
Beets, Canned																				750							
Black Beans, Canned																			331								
Blueberries, Cultivated																309	329						282				
Blueberries, Frozen																18	14						9				
Broccoli	151	620	668							698	708				170	676	509					602	613				
Butter												728									728	684					
Cabbage																			693	685						313	610
Cantaloupe							382	549	186			144	393	269					150	400	70						
Carrots	144	615	659	635	478				163	668	514				611	600						520	497				
Cauliflower													178	709	522					165	697	495					
Celery	498	624	172							694	691					698	704					641	652				
Cherries									275	285						403							174	168			
Cherries, Frozen																							103	263	62		
Cherry Tomatoes																				389	347						
Cilantro																		144	448								134
Corn Grain																660	650										
Corn Syrup							298	156																			
Cranberries															292										119	227	
Cranberries, Canned																											347
Cranberries, Frozen																									20	344	114
Cucumbers								379	392		76	375	284					331	299					180	254	80	
Eggplant														422	451												
Eggs																			350	335					271		
Fish, Catfish																	435	404	289								
Fish, Salmon																						95	101				
Garbanzo Beans, Canned																		166	168							144	412
Grape Juice							617	674									614					160	445				
Grapefruit	583	607												731	716									172	649	483	
Grapes	293	330	390	375	211				393	377			387	367				370	333					365	336		
Green Beans	426	446	484	482					581	552			480	106		588	565					306	562	560	368		
Green Beans, Canned					239	369	241					725	177										354				
Green Beans, Frozen					286	322	112							470									325				
Green Onions																	44	88									76
Greens, Collard															85	334	224										
Greens, Kale															84	327	280									568	567
Heavy Cream														362		720											258

Commodities Tested by PDP over time in Domestic Conventional Markets, Number of Samples per Commodity, Number of Foods Tested and Total Number of Samples per Year.

Commodities Tested by PDP	1992	1993	1994	1995	1996	1997	1998	1999	2000	2001	2002	2003	2004	2005	2006	2007	2008	2009	2010	2011	2012	2013	2014	2015	2016	2017	2018
Honey																127	345									195	
Hot Peppers																			114	268							
Infant Formula, Dairy																						163	487				
Infant Formula, Soy-based																						170	513				
Kidney Beans, Canned																	168	161									
Kiwi Fruit																											98
Lettuce	563	642	688					179	721	543			730	725					716	699				355	707	349	
Mangoes																			5							7	12
Milk					570	727	595						729	726						664					632	630	
Mushrooms										157	644	469								165	637	464					
Nectarines									340	358						344	441					301	466	352			
Oats Grain																			261				265				
Olives, Canned																									145	611	438
Onion											664	647								167	485					602	
Orange Juice						488	368						166	551	348				22	142	213						
Oranges	566	617	677	678	510				710	699			690	699				674	663					618	611		
Papaya																				76	88						
Peaches	223	214	274	253	199				273	263	275	267			66	357	411					254	495	179			
Peaches, Canned						745						716	720														708
Peaches-Single Servings									272																		
Peanut Butter									691						701									294			
Pear Juice, Conc./Puree												56															
Pears						587	613	279				180	634	439				612	640					592	586		
Pears, Canned								354	354																		
Pears-Single Servings							324	275																			
Pineapple, Canned																										23	
Pineapples									149	273	137																
Pinto Beans, Canned																		323									
Plums														336	313					137	399	227					
Plums, Dried (Prunes)														143	208											106	318
Pork Adipose														345													
Pork Muscle														345													
Potatoes	566	632	686	700					364	709	352						717	718						660	653		
Potatoes, Frozen															628	582											
Poultry Adipose									476	155																	
Poultry Liver									480	155																	
Poultry Muscle									145	154																	
Poultry, Breast															655												
Poultry, Thigh															655												
Raisins															361	343											639
Raspberries																						401					
Raspberries, Frozen																						6					
Rice									176	679	486						150	365					213				124
Snap Peas																				217	213					219	231
Soybean grain						159	590						616	973						300							
Spinach				601	486	488					332	681			472		687	673						630	624		
Spinach, Canned						168	687						366						181	198							
Spinach, Frozen								693											179	175							134
Strawberries							571	612	494				683	686			649	649					144	598	451		
Strawberries, Frozen							43	69	36																		68
Summer Squash															89	453	352				97	383	305				
Sweet Bell Peppers								518	540		151	515	391						421	464	64						
Sweet Corn, Can/Frozen			452	651	169					178	702	517					30	66	67				38	53			
Sweet Corn, Fresh																	119	559	357				117	397			
Sweet Peas, Can/Frozen			426	660	346					183	703	497			638												165
Sweet Potatoes					510	688	352					711	721				177	717	536						508	665	167
Tangerines																				449	404						

Commodities Tested by PDP over time in Domestic Conventional Markets, Number of Samples per Commodity, Number of Foods Tested and Total Number of Samples per Year.

Commodities Tested by PDP	1992	1993	1994	1995	1996	1997	1998	1999	2000	2001	2002	2003	2004	2005	2006	2007	2008	2009	2010	2011	2012	2013	2014	2015	2016	2017	2018
Tomato Paste										364								694									
Tomatoes					133	496	429	220				506	497			472	454						79	342	207		
Tomatoes, Canned								352	361																164	476	
Water, Bottled														320	316											609	
Water, Finished										296	669	794	381	374	368	370	310	306	284	119	232	50					
Water, Groundwater																272	250	278	250	604	168	14					
Water, Untreated													381	376	369	364	309	306	283	120	253	50					
Watermelon														114	286				212				277	84			
Wheat Flour												597	713														728
Wheat grain				600	340	623								674	687						300						
Winter Squash						255	344	102					299	405	100					119	368	27					
Winter Squash, Frozen						199	146	94																			
**Number of Foods Tested:**	**11**	**12**	**12**	**11**	**15**	**15**	**18**	**18**	**24**	**25**	**23**	**22**	**23**	**26**	**27**	**23**	**30**	**25**	**31**	**27**	**24**	**24**	**25**	**20**	**21**	**21**	**22**
**Total Samples per Year:**	**4,551**	**5,983**	**6,246**	**6,294**	**5,141**	**6,829**	**7,138**	**7,219**	**8,752**	**10,022**	**10,296**	**10,758**	**11,295**	**12,381**	**10,902**	**9,583**	**10,333**	**9,973**	**9,414**	**9,166**	**7,661**	**6,835**	**7,685**	**7,510**	**8,016**	**7,152**	**6,431**
