# Supplementary material for: The dietary risk index system: a tool to track pesticide dietary risks
Source: Environ Health. 2020 Oct 14;19:103. doi: 10.1186/s12940-020-00657-z (PMC7557078; doi:10.1186/s12940-020-00657-z)
Supplement: Supplementary file 7 — Additional file 7. Levels of Parathion Methyl in Domestic Conventional Peaches Tested by the US-PDP in 1996. [file 12940_2020_657_MOESM7_ESM.pdf]

Additional File 7 for "The Dietary Risk Index System: A Tool to Track Pesticide Dietary Risks," by Benbrook and Davis

Levels of Parathion Methyl in Domestic Conventional Peaches Tested by the US PDP in 1996

Sample ID	Parathion Methyl Levels (ppm)	DRI
3596	0.5	15.6
3705	0.34	10.6
3590	0.21	6.56
3859	0.19	5.94
3822	0.19	5.94
3606	0.17	5.31
3589	0.16	5.00
3607	0.15	4.69
3678	0.11	3.44
3578	0.11	3.44
3573	0.1	3.13
3581	0.1	3.13
3756	0.094	2.94
3577	0.094	2.94
3618	0.089	2.78
3687	0.086	2.69
3733	0.081	2.53
3842	0.079	2.47
3830	0.071	2.22
3655	0.068	2.13
3686	0.067	2.09
3736	0.067	2.09
3753	0.06	1.88
3751	0.056	1.75
3799	0.056	1.75
3757	0.054	1.69
3603	0.053	1.66
3832	0.052	1.63
3652	0.051	1.59
3857	0.05	1.56
3583	0.047	1.47
3746	0.046	1.44
3592	0.046	1.44
3579	0.046	1.44
3685	0.044	1.38
3605	0.043	1.34
3582	0.038	1.19
3843	0.036	1.13
3744	0.033	1.03
3693	0.032	1.00
3591	0.032	1.00
3755	0.029	0.906
3602	0.028	0.875
3684	0.024	0.750

Levels of Parathion Methyl in Domestic Conventional Peaches Tested by the US PDP in 1996

Sample ID	Parathion Methyl Levels (ppm)	DRI
3649	0.022	0.688
3645	0.022	0.688
3650	0.022	0.688
3659	0.022	0.688
3792	0.022	0.688
3707	0.022	0.688
3788	0.022	0.688
3708	0.022	0.688
3791	0.022	0.688
3836	0.022	0.688
3833	0.022	0.688
3831	0.022	0.688
3829	0.022	0.688
3790	0.022	0.688
3783	0.022	0.688
3599	0.016	0.500
3752	0.016	0.500
3586	0.014	0.438
3600	0.013	0.406
3604	0.011	0.344
3854	0.01	0.313
3690	0.01	0.313
3855	0.01	0.313
3695	0.01	0.313
3694	0.01	0.313
3765	0.01	0.313
3766	0.009	0.281
3760	0.009	0.281
3759	0.005	0.156
3747	0.005	0.156
3745	0.005	0.156
3743	0.005	0.156
3742	0.005	0.156
3737	0.005	0.156
3734	0.005	0.156
3570	0.005	0.156
3764	0.005	0.156
3610	0.004	0.125
