# Supplementary material for: The dietary risk index system: a tool to track pesticide dietary risks
Source: Environ Health. 2020 Oct 14;19:103. doi: 10.1186/s12940-020-00657-z (PMC7557078; doi:10.1186/s12940-020-00657-z)
Supplement: Supplementary file 8 — Additional file 8. Aggregate DRI Values for All Pesticides in Domestic Samples, US-PDP, 1996 [No Banned OCs; Rule of 10 Imposed]. [file 12940_2020_657_MOESM8_ESM.pdf]

US-PDP - All Domestic Samples: Aggregate Food DRI Values by Year, Ranked by FS-DRI (Highest to Lowest) across All Pesticides. Year 1996 [No Banned OC's; Rule of 10 Imposed]

Food	PDP Year	Average Number of Samples per Pesticide	Number of Positives	Average Number of Residues per Sample	Positive Sample DRI-M	Percent of Total DRI-M	Total FS-DRI	Percent of Total FS-DRI
Peaches	1996	195	591	3.03	4.0916	8.0%	1.0469	27.91%
Apples	1996	463	1,779	3.84	21.0576	41.1%	0.7115	18.97%
Grapes	1996	194	288	1.48	7.6141	14.9%	0.5421	14.45%
Green Beans, Frozen	1996	242	419	1.73	2.1996	4.3%	0.4067	10.84%
Tomatoes	1996	123	168	1.37	4.0599	7.9%	0.3779	10.08%
Green Beans, Canned	1996	198	199	1.01	1.0188	2.0%	0.1799	4.80%
Wheat grain	1996	340	577	1.70	2.4213	4.7%	0.1542	4.11%
Spinach	1996	415	711	1.71	2.9554	5.8%	0.1227	3.27%
Sweet Potatoes	1996	476	456	0.96	1.0401	2.0%	0.0643	1.71%
Oranges	1996	466	785	1.68	0.9595	1.9%	0.0609	1.62%
Apple Juice	1996	144	191	1.33	0.5635	1.1%	0.0549	1.46%
Carrots	1996	422	565	1.34	2.8657	5.6%	0.0215	0.57%
Sweet Peas, Can/Frozen	1996	346	101	0.29	0.3292	0.6%	0.0071	0.19%
Milk	1996	436	6	0.01	0.0767	0.1%	0.0002	0.01%
Sum of Average Number of Samples:		4,460						
Total Positives and Aggregate DRI:			6,836		51.3		3.75	
Average DRI Detections per Sample:			1.53					
Number of Foods Tested:		14						
Average DRI per Food Tested:					3.66		0.268	
