# Supplementary material for: The dietary risk index system: a tool to track pesticide dietary risks
Source: Environ Health. 2020 Oct 14;19:103. doi: 10.1186/s12940-020-00657-z (PMC7557078; doi:10.1186/s12940-020-00657-z)
Supplement: Supplementary file 9 — Additional file 9. Pesticide Residues and DRI values in Potatoes, US-PDP, 2015 (Conventional, Domestic Samples). [file 12940_2020_657_MOESM9_ESM.pdf]

Additional File 9 for "The Dietary Risk Index System: A Tool to Track Pesticide Dietary Risks," by Benbrook and Davis

Pesticide Residue and Risk Indicators in Potatoes Ranked by Percent of Aggregate FS-DRI: Conventionally Grown Samples, Domestically Grown Potatoes Tested by PDP in 2015

Pesticide	Total Number of Samples Tested	Number of Positives	Percent Positive	Mean Residue (ppm)	cRfC (ppm)	DRI-M	FS-DRI	Percent of Aggregate FS-DRI
Chlorpropham	660	650	98.5%	1.86	10.67	0.175	0.172	84.5%
Parathion oxygen analog	660	3	0.455%	0.0200	0.00640	3.125	0.0142	6.97%
Difenoconazole	660	58	8.79%	0.200	2.133	0.0935	0.00822	4.03%
Fipronil	660	22	3.33%	0.00318	0.0427	0.0745	0.00248	1.22%
Fludioxonil	660	36	5.45%	0.232	6.40	0.0363	0.00198	0.970%
Thiabendazole	660	29	4.39%	0.237	7.04	0.0336	0.00148	0.724%
Phorate sulfoxide	660	4	0.606%	0.0148	0.107	0.139	0.000839	0.412%
Phorate sulfone	660	3	0.455%	0.0160	0.11	0.150	0.000682	0.335%
Imidacloprid	660	320	48.5%	0.00979	12.16	0.000805	0.000390	0.191%
Pentachloroaniline (PCA)	660	47	7.12%	0.0101	2.13	0.00472	0.000336	0.165%
Azoxystrobin	660	217	32.9%	0.0372	38.4	0.00097	0.000318	0.156%
Thiamethoxam	660	42	6.36%	0.0118	2.56	0.00461	0.000293	0.144%
Pentachlorobenzene (PCB)	660	11	1.67%	0.0105	2.13	0.00490	0.0000817	0.0401%
Bifenthrin	660	32	4.85%	0.00450	2.77	0.00162	0.0000787	0.0386%
Quintozene (PCNB)	660	16	2.42%	0.00669	2.13	0.00314	0.0000761	0.0373%
Clothianidin	660	147	22.3%	0.00617	20.9	0.00030	0.0000658	0.0323%
Dicofol p,p'	660	1	0.152%	0.00200	0.0853	0.0234	0.0000355	0.0174%
Endosulfan sulfate	603	1	0.166%	0.0180	1.28	0.0141	0.0000233	0.0114%
Metalaxyl	660	58	8.79%	0.00380	15.8	0.00024	0.0000211	0.0104%
Acephate	660	1	0.152%	0.00250	0.256	0.00977	0.0000148	0.00726%
Fluxapyroxad	660	7	1.06%	0.00503	4.48	0.00112	0.0000119	0.00584%
Metribuzin	660	6	0.91%	0.00250	2.77	0.000901	0.00000819	0.00402%
Boscalid	660	12	1.82%	0.00980	46.5	0.000211	0.00000383	0.00188%
Pyraclostrobin	660	2	0.303%	0.00335	7.25	0.000462	0.00000140	0.000687%
Tetrahydrophthalimide (THPI)	660	3	0.455%	0.00630	26.7	0.000236	0.00000107	0.000527%
Norflurazon desmethyl	660	1	0.152%	0.00200	3.20	0.000625	0.000000947	0.000465%
Fluoxastrobin	660	1	0.152%	0.00200	3.20	0.000625	0.000000947	0.000465%
Pendimethalin	660	13	1.97%	0.00227	64.0	0.000035	0.000000698	0.000343%
Etofenprox	660	1	0.152%	0.00250	10.7	0.000234	0.000000355	0.000174%
Flonicamid	660	1	0.152%	0.00200	8.53	0.000234	0.000000355	0.000174%
Chlorantraniliprole	642	14	2.18%	0.00419	337	0.0000124	0.000000271	0.000133%
Penthiopyrad	660	3	0.455%	0.00250	57.6	0.0000434	0.000000197	0.0000968%
Metolachlor	660	1	0.152%	0.00200	21.3	0.0000938	0.000000142	0.0000697%
