# Supplementary material for: The dietary risk index system: a tool to track pesticide dietary risks
Source: Environ Health. 2020 Oct 14;19:103. doi: 10.1186/s12940-020-00657-z (PMC7557078; doi:10.1186/s12940-020-00657-z)
Supplement: Supplementary file 10 — Additional file 10. Individual-Sample DRI Values in Apples, US-PDP, 2009 (Conventional, Domestic Samples). [file 12940_2020_657_MOESM10_ESM.pdf]

US-PDP: Individual Positive Samples, Ranked by Aggregate Sample DRI (Highest - Lowest), for Domestic Conventional Apples Tested in 2009

Ranking	PDP Sample ID	Origin	Market Claim	State	Number of Residues	Aggregate Sample DRI
1	626	Domestic	Conventional	Washington	7	8.74812
2	270	Domestic	Conventional	Washington	5	3.71747
3	23	Domestic	Conventional	Washington	5	3.56590
4	649	Domestic	Conventional	Washington	8	3.02831
5	97	Domestic	Conventional	Washington	6	2.78421
6	152	Domestic	Conventional	Washington	5	2.75022
7	101	Domestic	Conventional	California	3	2.51624
8	324	Domestic	Conventional	Washington	10	1.62799
9	262	Domestic	Conventional	Washington	7	1.41120
10	117	Domestic	Conventional	Washington	3	1.33414
11	297	Domestic	Conventional	Washington	6	1.29717
12	260	Domestic	Conventional	North Carolina	8	1.29144
13	91	Domestic	Conventional	Washington	5	1.17487
14	93	Domestic	Conventional	Washington	3	1.14797
15	122	Domestic	Conventional	Washington	2	1.09532
16	615	Domestic	Conventional	Washington	7	1.09393
17	732	Domestic	Conventional	Texas	8	1.04940
18	298	Domestic	Conventional	Washington	5	0.97013
19	691	Domestic	Conventional	Washington	7	0.96037
20	358	Domestic	Conventional	Washington	10	0.94581
21	705	Domestic	Conventional	Washington	7	0.94248
22	84	Domestic	Conventional	Washington	6	0.89152
23	352	Domestic	Conventional	Washington	10	0.88778
24	488	Domestic	Conventional	Unknown	7	0.88237
25	237	Domestic	Conventional	Washington	6	0.86215
26	163	Domestic	Conventional	Washington	9	0.83793
27	121	Domestic	Conventional	California	3	0.79481
28	713	Domestic	Conventional	Washington	5	0.78728
29	570	Domestic	Conventional	Washington	8	0.74855
30	605	Domestic	Conventional	Ohio	6	0.72051
31	107	Domestic	Conventional	Washington	6	0.70739
32	131	Domestic	Conventional	Washington	4	0.70699

US-PDP: Individual Positive Samples, Ranked by Aggregate Sample DRI (Highest - Lowest), for Domestic Conventional Apples Tested in 2009

Ranking	PDP Sample ID	Origin	Market Claim	State	Number of Residues	Aggregate Sample DRI
33	143	Domestic	Conventional	Washington	3	0.70079
34	156	Domestic	Conventional	Washington	5	0.69188
35	58	Domestic	Conventional	Washington	9	0.68182
36	242	Domestic	Conventional	Washington	5	0.66974
37	609	Domestic	Conventional	Texas	9	0.66486
38	3	Domestic	Conventional	Washington	5	0.65416
39	690	Domestic	Conventional	Washington	8	0.64795
40	31	Domestic	Conventional	Washington	6	0.62713
41	533	Domestic	Conventional	Washington	7	0.62681
42	244	Domestic	Conventional	Minnesota	5	0.62371
43	736	Domestic	Conventional	Washington	11	0.59561
44	174	Domestic	Conventional	Texas	5	0.59162
45	73	Domestic	Conventional	Washington	3	0.57641
46	158	Domestic	Conventional	Washington	2	0.57324
47	150	Domestic	Conventional	Washington	1	0.56439
48	238	Domestic	Conventional	Washington	6	0.54323
49	693	Domestic	Conventional	Washington	5	0.53834
50	78	Domestic	Conventional	Washington	6	0.53522
51	267	Domestic	Conventional	Washington	7	0.52764
52	76	Domestic	Conventional	Washington	8	0.52346
53	698	Domestic	Conventional	Washington	3	0.52037
54	41	Domestic	Conventional	Washington	5	0.51802
55	128	Domestic	Conventional	California	4	0.50973
56	312	Domestic	Conventional	Washington	5	0.49145
57	715	Domestic	Conventional	Washington	8	0.49113
58	456	Domestic	Conventional	Washington	6	0.48987
59	360	Domestic	Conventional	Washington	10	0.47343
60	648	Domestic	Conventional	Washington	2	0.46660
61	250	Domestic	Conventional	Idaho	7	0.46518
62	716	Domestic	Conventional	Washington	6	0.46327
63	525	Domestic	Conventional	Washington	8	0.45862
64	48	Domestic	Conventional	Washington	10	0.45205

US-PDP: Individual Positive Samples, Ranked by Aggregate Sample DRI (Highest - Lowest), for Domestic Conventional Apples Tested in 2009

Ranking	PDP Sample ID	Origin	Market Claim	State	Number of Residues	Aggregate Sample DRI
65	639	Domestic	Conventional	Washington	6	0.45149
66	614	Domestic	Conventional	Washington	5	0.44635
67	70	Domestic	Conventional	Washington	3	0.43985
68	611	Domestic	Conventional	Washington	6	0.43782
69	99	Domestic	Conventional	Washington	3	0.43716
70	531	Domestic	Conventional	Washington	6	0.43549
71	12	Domestic	Conventional	Washington	8	0.43168
72	220	Domestic	Conventional	Washington	5	0.42752
73	439	Domestic	Conventional	Washington	3	0.42563
74	106	Domestic	Conventional	Washington	2	0.42424
75	165	Domestic	Conventional	Washington	8	0.42281
76	596	Domestic	Conventional	Washington	11	0.42195
77	523	Domestic	Conventional	Washington	8	0.41600
78	67	Domestic	Conventional	Washington	2	0.41575
79	638	Domestic	Conventional	Washington	7	0.40921
80	618	Domestic	Conventional	Washington	3	0.40672
81	383	Domestic	Conventional	Washington	3	0.40598
82	157	Domestic	Conventional	California	5	0.40357
83	5	Domestic	Conventional	Washington	4	0.39570
84	672	Domestic	Conventional	Michigan	10	0.39357
85	24	Domestic	Conventional	Idaho	1	0.38906
86	339	Domestic	Conventional	Washington	8	0.38475
87	92	Domestic	Conventional	Washington	4	0.38260
88	2	Domestic	Conventional	Washington	3	0.37794
89	674	Domestic	Conventional	Washington	3	0.37360
90	171	Domestic	Conventional	Washington	7	0.36925
91	415	Domestic	Conventional	New York	5	0.36923
92	441	Domestic	Conventional	Washington	7	0.36593
93	450	Domestic	Conventional	Washington	5	0.36205
94	87	Domestic	Conventional	Washington	6	0.35917
95	32	Domestic	Conventional	Washington	4	0.35725
96	607	Domestic	Conventional	Washington	6	0.35628

US-PDP: Individual Positive Samples, Ranked by Aggregate Sample DRI (Highest - Lowest), for Domestic Conventional Apples Tested in 2009

Ranking	PDP Sample ID	Origin	Market Claim	State	Number of Residues	Aggregate Sample DRI
97	154	Domestic	Conventional	Washington	3	0.35139
98	571	Domestic	Conventional	Washington	5	0.34779
99	681	Domestic	Conventional	Washington	4	0.34426
100	510	Domestic	Conventional	Washington	4	0.34065
101	255	Domestic	Conventional	Washington	6	0.33518
102	120	Domestic	Conventional	California	3	0.33516
103	265	Domestic	Conventional	Washington	5	0.33391
104	504	Domestic	Conventional	Washington	10	0.33045
105	181	Domestic	Conventional	Washington	7	0.32862
106	247	Domestic	Conventional	Washington	6	0.32712
107	351	Domestic	Conventional	Washington	7	0.32474
108	733	Domestic	Conventional	Washington	5	0.32140
109	590	Domestic	Conventional	Washington	3	0.32089
110	632	Domestic	Conventional	Texas	4	0.31735
111	175	Domestic	Conventional	Maryland	10	0.31675
112	182	Domestic	Conventional	Washington	13	0.31454
113	373	Domestic	Conventional	Unknown	4	0.31385
114	190	Domestic	Conventional	Washington	3	0.31222
115	679	Domestic	Conventional	Washington	5	0.31044
116	187	Domestic	Conventional	Indiana	6	0.30756
117	151	Domestic	Conventional	Washington	3	0.30707
118	72	Domestic	Conventional	Washington	3	0.30623
119	442	Domestic	Conventional	Maine	5	0.30520
120	479	Domestic	Conventional	New York	8	0.30402
121	629	Domestic	Conventional	Unknown	6	0.30215
122	554	Domestic	Conventional	Michigan	7	0.30192
123	496	Domestic	Conventional	New York	8	0.30042
124	364	Domestic	Conventional	Washington	6	0.29795
125	677	Domestic	Conventional	Washington	6	0.29626
126	189	Domestic	Conventional	Washington	6	0.29010
127	432	Domestic	Conventional	Washington	3	0.28927
128	199	Domestic	Conventional	Washington	6	0.28838

US-PDP: Individual Positive Samples, Ranked by Aggregate Sample DRI (Highest - Lowest), for Domestic Conventional Apples Tested in 2009

Ranking	PDP Sample ID	Origin	Market Claim	State	Number of Residues	Aggregate Sample DRI
129	438	Domestic	Conventional	Washington	5	0.28788
130	279	Domestic	Conventional	Washington	8	0.28771
131	15	Domestic	Conventional	Washington	4	0.28733
132	532	Domestic	Conventional	Washington	3	0.28401
133	96	Domestic	Conventional	Washington	5	0.28344
134	513	Domestic	Conventional	Washington	5	0.28315
135	521	Domestic	Conventional	Washington	3	0.28308
136	669	Domestic	Conventional	Texas	3	0.28182
137	541	Domestic	Conventional	Washington	6	0.27979
138	347	Domestic	Conventional	Washington	9	0.27795
139	597	Domestic	Conventional	Washington	7	0.27745
140	524	Domestic	Conventional	Washington	7	0.27497
141	517	Domestic	Conventional	Washington	5	0.27352
142	49	Domestic	Conventional	Washington	3	0.27116
143	313	Domestic	Conventional	Washington	3	0.26951
144	610	Domestic	Conventional	Washington	6	0.26697
145	334	Domestic	Conventional	Washington	6	0.26593
146	435	Domestic	Conventional	New York	7	0.26585
147	218	Domestic	Conventional	Washington	5	0.26351
148	266	Domestic	Conventional	Washington	7	0.26113
149	399	Domestic	Conventional	Washington	9	0.25972
150	271	Domestic	Conventional	Washington	7	0.25923
151	505	Domestic	Conventional	Washington	7	0.25497
152	413	Domestic	Conventional	Washington	10	0.25417
153	215	Domestic	Conventional	Washington	4	0.25186
154	535	Domestic	Conventional	Washington	7	0.25180
155	326	Domestic	Conventional	Unknown	7	0.25124
156	4	Domestic	Conventional	Washington	3	0.24604
157	591	Domestic	Conventional	Washington	3	0.24561
158	362	Domestic	Conventional	Washington	2	0.24519
159	416	Domestic	Conventional	New York	9	0.24484
160	42	Domestic	Conventional	Washington	2	0.24401

US-PDP: Individual Positive Samples, Ranked by Aggregate Sample DRI (Highest - Lowest), for Domestic Conventional Apples Tested in 2009

Ranking	PDP Sample ID	Origin	Market Claim	State	Number of Residues	Aggregate Sample DRI
161	623	Domestic	Conventional	Washington	5	0.24191
162	544	Domestic	Conventional	Washington	4	0.24131
163	706	Domestic	Conventional	Washington	6	0.23972
164	103	Domestic	Conventional	Washington	5	0.23849
165	19	Domestic	Conventional	Washington	3	0.23822
166	540	Domestic	Conventional	Washington	10	0.23713
167	666	Domestic	Conventional	Washington	5	0.23590
168	25	Domestic	Conventional	Washington	1	0.23516
169	536	Domestic	Conventional	Washington	5	0.23511
170	195	Domestic	Conventional	New York	8	0.23349
171	582	Domestic	Conventional	Washington	11	0.23290
172	350	Domestic	Conventional	Washington	7	0.23059
173	372	Domestic	Conventional	Unknown	5	0.22746
174	308	Domestic	Conventional	West Virginia	5	0.22656
175	458	Domestic	Conventional	California	1	0.22576
176	446	Domestic	Conventional	New York	8	0.22394
177	288	Domestic	Conventional	Washington	5	0.22360
178	277	Domestic	Conventional	Maryland	6	0.22342
179	593	Domestic	Conventional	Texas	10	0.22218
180	684	Domestic	Conventional	Washington	3	0.22144
181	85	Domestic	Conventional	Washington	5	0.21905
182	498	Domestic	Conventional	New York	6	0.21772
183	612	Domestic	Conventional	Washington	8	0.21576
184	210	Domestic	Conventional	Massachusetts	6	0.21529
185	722	Domestic	Conventional	Washington	6	0.21445
186	66	Domestic	Conventional	Washington	2	0.21277
187	394	Domestic	Conventional	Washington	4	0.21274
188	335	Domestic	Conventional	Washington	8	0.21214
189	196	Domestic	Conventional	Washington	5	0.21117
190	226	Domestic	Conventional	Washington	3	0.21059
191	170	Domestic	Conventional	Texas	7	0.21009
192	650	Domestic	Conventional	Texas	10	0.20983

US-PDP: Individual Positive Samples, Ranked by Aggregate Sample DRI (Highest - Lowest), for Domestic Conventional Apples Tested in 2009

Ranking	PDP Sample ID	Origin	Market Claim	State	Number of Residues	Aggregate Sample DRI
193	306	Domestic	Conventional	Virginia	5	0.20700
194	619	Domestic	Conventional	Washington	7	0.20609
195	606	Domestic	Conventional	Washington	4	0.20404
196	191	Domestic	Conventional	Washington	6	0.20328
197	718	Domestic	Conventional	Washington	8	0.20117
198	671	Domestic	Conventional	Washington	9	0.20071
199	548	Domestic	Conventional	Washington	6	0.20063
200	290	Domestic	Conventional	New York	8	0.19844
201	123	Domestic	Conventional	Washington	4	0.19687
202	367	Domestic	Conventional	Washington	6	0.19543
203	714	Domestic	Conventional	Washington	6	0.19459
204	426	Domestic	Conventional	New York	10	0.19457
205	217	Domestic	Conventional	Washington	9	0.19373
206	422	Domestic	Conventional	New York	9	0.19258
207	724	Domestic	Conventional	Washington	8	0.19185
208	421	Domestic	Conventional	Washington	11	0.19114
209	703	Domestic	Conventional	Washington	8	0.18995
210	670	Domestic	Conventional	Washington	6	0.18956
211	528	Domestic	Conventional	Washington	3	0.18858
212	159	Domestic	Conventional	Washington	5	0.18790
213	729	Domestic	Conventional	New York	7	0.18751
214	33	Domestic	Conventional	Washington	5	0.18710
215	553	Domestic	Conventional	Ohio	7	0.18686
216	587	Domestic	Conventional	Washington	5	0.18625
217	675	Domestic	Conventional	Washington	10	0.18611
218	235	Domestic	Conventional	Washington	3	0.18606
219	637	Domestic	Conventional	Washington	8	0.18578
220	79	Domestic	Conventional	Washington	6	0.18490
221	493	Domestic	Conventional	Washington	6	0.18436
222	710	Domestic	Conventional	Unknown	3	0.18312
223	261	Domestic	Conventional	Washington	3	0.18303
224	508	Domestic	Conventional	Michigan	7	0.18298

US-PDP: Individual Positive Samples, Ranked by Aggregate Sample DRI (Highest - Lowest), for Domestic Conventional Apples Tested in 2009

Ranking	PDP Sample ID	Origin	Market Claim	State	Number of Residues	Aggregate Sample DRI
225	717	Domestic	Conventional	Washington	6	0.18286
226	585	Domestic	Conventional	Texas	8	0.18227
227	731	Domestic	Conventional	Washington	8	0.18077
228	492	Domestic	Conventional	New York	9	0.17997
229	499	Domestic	Conventional	Washington	8	0.17961
230	236	Domestic	Conventional	Washington	5	0.17863
231	17	Domestic	Conventional	Washington	2	0.17815
232	530	Domestic	Conventional	Washington	8	0.17792
233	688	Domestic	Conventional	Washington	5	0.17748
234	417	Domestic	Conventional	Washington	6	0.17642
235	36	Domestic	Conventional	Washington	6	0.17599
236	616	Domestic	Conventional	Washington	8	0.17295
237	272	Domestic	Conventional	New York	8	0.17284
238	126	Domestic	Conventional	Washington	4	0.17275
239	624	Domestic	Conventional	Washington	3	0.17266
240	699	Domestic	Conventional	Washington	8	0.17245
241	480	Domestic	Conventional	Washington	5	0.17190
242	264	Domestic	Conventional	Washington	5	0.17092
243	635	Domestic	Conventional	Washington	6	0.17062
244	604	Domestic	Conventional	Washington	6	0.17002
245	598	Domestic	Conventional	Washington	6	0.16875
246	291	Domestic	Conventional	Washington	7	0.16823
247	412	Domestic	Conventional	Washington	4	0.16805
248	741	Domestic	Conventional	Washington	9	0.16766
249	52	Domestic	Conventional	Washington	4	0.16712
250	447	Domestic	Conventional	Washington	7	0.16639
251	88	Domestic	Conventional	Washington	3	0.16492
252	153	Domestic	Conventional	Washington	3	0.16437
253	162	Domestic	Conventional	Washington	6	0.16069
254	538	Domestic	Conventional	Washington	4	0.15972
255	673	Domestic	Conventional	Washington	5	0.15823
256	402	Domestic	Conventional	New York	7	0.15763

US-PDP: Individual Positive Samples, Ranked by Aggregate Sample DRI (Highest - Lowest), for Domestic Conventional Apples Tested in 2009

Ranking	PDP Sample ID	Origin	Market Claim	State	Number of Residues	Aggregate Sample DRI
257	401	Domestic	Conventional	New York	8	0.15710
258	428	Domestic	Conventional	New York	10	0.15508
259	203	Domestic	Conventional	Washington	8	0.15467
260	405	Domestic	Conventional	Washington	10	0.15434
261	208	Domestic	Conventional	Washington	8	0.15433
262	14	Domestic	Conventional	Washington	4	0.15412
263	230	Domestic	Conventional	Indiana	7	0.15404
264	125	Domestic	Conventional	Washington	3	0.15232
265	565	Domestic	Conventional	Michigan	3	0.15232
266	81	Domestic	Conventional	Washington	7	0.15230
267	90	Domestic	Conventional	Washington	7	0.15219
268	545	Domestic	Conventional	Washington	4	0.15025
269	481	Domestic	Conventional	New York	9	0.14991
270	592	Domestic	Conventional	New York	7	0.14986
271	563	Domestic	Conventional	Washington	10	0.14741
272	654	Domestic	Conventional	Washington	13	0.14719
273	485	Domestic	Conventional	Pennsylvania	9	0.14716
274	194	Domestic	Conventional	Washington	6	0.14692
275	701	Domestic	Conventional	Washington	4	0.14629
276	37	Domestic	Conventional	Washington	6	0.14589
277	436	Domestic	Conventional	New York	9	0.14395
278	62	Domestic	Conventional	Washington	2	0.14353
279	559	Domestic	Conventional	Ohio	4	0.14263
280	444	Domestic	Conventional	New York	8	0.14212
281	625	Domestic	Conventional	Washington	6	0.14126
282	739	Domestic	Conventional	Texas	9	0.14065
283	527	Domestic	Conventional	Washington	2	0.14021
284	54	Domestic	Conventional	Washington	4	0.13988
285	64	Domestic	Conventional	Washington	7	0.13953
286	296	Domestic	Conventional	Texas	8	0.13932
287	687	Domestic	Conventional	Texas	3	0.13918
288	180	Domestic	Conventional	Washington	6	0.13852

US-PDP: Individual Positive Samples, Ranked by Aggregate Sample DRI (Highest - Lowest), for Domestic Conventional Apples Tested in 2009

Ranking	PDP Sample ID	Origin	Market Claim	State	Number of Residues	Aggregate Sample DRI
289	276	Domestic	Conventional	Washington	9	0.13747
290	692	Domestic	Conventional	Washington	5	0.13743
291	221	Domestic	Conventional	Minnesota	5	0.13728
292	219	Domestic	Conventional	Washington	2	0.13698
293	294	Domestic	Conventional	Maryland	4	0.13687
294	299	Domestic	Conventional	Washington	6	0.13361
295	425	Domestic	Conventional	New York	8	0.13337
296	29	Domestic	Conventional	Washington	5	0.13329
297	327	Domestic	Conventional	Washington	4	0.13287
298	241	Domestic	Conventional	Washington	5	0.13249
299	269	Domestic	Conventional	Washington	6	0.13240
300	572	Domestic	Conventional	Washington	3	0.13211
301	451	Domestic	Conventional	New York	9	0.13121
302	6	Domestic	Conventional	Washington	4	0.13118
303	656	Domestic	Conventional	Washington	7	0.12902
304	398	Domestic	Conventional	New York	5	0.12835
305	423	Domestic	Conventional	New York	6	0.12741
306	201	Domestic	Conventional	Washington	4	0.12741
307	286	Domestic	Conventional	Unknown	3	0.12578
308	579	Domestic	Conventional	Washington	6	0.12568
309	395	Domestic	Conventional	New York	8	0.12556
310	102	Domestic	Conventional	Washington	2	0.12541
311	86	Domestic	Conventional	Washington	4	0.12478
312	231	Domestic	Conventional	Washington	5	0.12445
313	551	Domestic	Conventional	Washington	7	0.12421
314	728	Domestic	Conventional	Washington	5	0.12387
315	89	Domestic	Conventional	Washington	4	0.12358
316	146	Domestic	Conventional	California	5	0.12340
317	406	Domestic	Conventional	New York	7	0.12194
318	225	Domestic	Conventional	Washington	3	0.12151
319	470	Domestic	Conventional	Unknown	7	0.12045
320	676	Domestic	Conventional	Washington	3	0.12029

US-PDP: Individual Positive Samples, Ranked by Aggregate Sample DRI (Highest - Lowest), for Domestic Conventional Apples Tested in 2009

Ranking	PDP Sample ID	Origin	Market Claim	State	Number of Residues	Aggregate Sample DRI
321	495	Domestic	Conventional	Washington	7	0.12027
322	343	Domestic	Conventional	Washington	4	0.11854
323	356	Domestic	Conventional	Washington	3	0.11795
324	160	Domestic	Conventional	Washington	3	0.11758
325	249	Domestic	Conventional	Washington	5	0.11727
326	468	Domestic	Conventional	Washington	9	0.11721
327	368	Domestic	Conventional	Washington	2	0.11411
328	539	Domestic	Conventional	Washington	5	0.11401
329	686	Domestic	Conventional	Washington	12	0.11342
330	636	Domestic	Conventional	Washington	4	0.11289
331	392	Domestic	Conventional	Washington	6	0.11275
332	555	Domestic	Conventional	Ohio	4	0.11203
333	655	Domestic	Conventional	Washington	6	0.11186
334	183	Domestic	Conventional	Washington	8	0.11171
335	568	Domestic	Conventional	Washington	7	0.11154
336	695	Domestic	Conventional	Washington	3	0.11137
337	500	Domestic	Conventional	Washington	8	0.11075
338	700	Domestic	Conventional	Washington	6	0.10990
339	617	Domestic	Conventional	Washington	3	0.10970
340	178	Domestic	Conventional	California	11	0.10951
341	27	Domestic	Conventional	California	3	0.10917
342	719	Domestic	Conventional	Washington	3	0.10904
343	357	Domestic	Conventional	Washington	8	0.10766
344	599	Domestic	Conventional	California	6	0.10764
345	738	Domestic	Conventional	Texas	8	0.10751
346	209	Domestic	Conventional	New York	5	0.10725
347	47	Domestic	Conventional	Washington	8	0.10670
348	370	Domestic	Conventional	Washington	3	0.10667
349	268	Domestic	Conventional	Michigan	5	0.10652
350	627	Domestic	Conventional	Washington	6	0.10624
351	147	Domestic	Conventional	Washington	3	0.10624
352	461	Domestic	Conventional	New York	5	0.10598

US-PDP: Individual Positive Samples, Ranked by Aggregate Sample DRI (Highest - Lowest), for Domestic Conventional Apples Tested in 2009

Ranking	PDP Sample ID	Origin	Market Claim	State	Number of Residues	Aggregate Sample DRI
353	136	Domestic	Conventional	Washington	5	0.10580
354	622	Domestic	Conventional	Washington	4	0.10495
355	302	Domestic	Conventional	Washington	5	0.10483
356	363	Domestic	Conventional	Washington	3	0.10407
357	391	Domestic	Conventional	Washington	6	0.10404
358	580	Domestic	Conventional	Washington	11	0.10402
359	319	Domestic	Conventional	Michigan	6	0.10380
360	245	Domestic	Conventional	New York	9	0.10355
361	449	Domestic	Conventional	Washington	6	0.10352
362	314	Domestic	Conventional	Washington	1	0.10347
363	223	Domestic	Conventional	Washington	4	0.10301
364	463	Domestic	Conventional	New York	10	0.10277
365	502	Domestic	Conventional	Washington	7	0.10228
366	371	Domestic	Conventional	Washington	4	0.10208
367	519	Domestic	Conventional	Washington	3	0.10205
368	652	Domestic	Conventional	Washington	5	0.09986
369	529	Domestic	Conventional	Washington	8	0.09964
370	307	Domestic	Conventional	Washington	6	0.09961
371	198	Domestic	Conventional	Texas	6	0.09946
372	471	Domestic	Conventional	New York	6	0.09912
373	287	Domestic	Conventional	Washington	7	0.09899
374	630	Domestic	Conventional	Washington	7	0.09879
375	520	Domestic	Conventional	Washington	11	0.09873
376	734	Domestic	Conventional	Washington	4	0.09856
377	602	Domestic	Conventional	Washington	5	0.09765
378	135	Domestic	Conventional	Washington	6	0.09755
379	301	Domestic	Conventional	Maryland	2	0.09744
380	345	Domestic	Conventional	Washington	6	0.09665
381	725	Domestic	Conventional	Unknown	3	0.09638
382	222	Domestic	Conventional	Washington	9	0.09635
383	483	Domestic	Conventional	New York	7	0.09600
384	410	Domestic	Conventional	New York	8	0.09598

US-PDP: Individual Positive Samples, Ranked by Aggregate Sample DRI (Highest - Lowest), for Domestic Conventional Apples Tested in 2009

Ranking	PDP Sample ID	Origin	Market Claim	State	Number of Residues	Aggregate Sample DRI
385	341	Domestic	Conventional	Unknown	1	0.09595
386	601	Domestic	Conventional	Washington	9	0.09560
387	509	Domestic	Conventional	Washington	10	0.09480
388	704	Domestic	Conventional	Washington	3	0.09434
389	204	Domestic	Conventional	Florida	6	0.09417
390	409	Domestic	Conventional	Washington	3	0.09355
391	730	Domestic	Conventional	Washington	4	0.09302
392	645	Domestic	Conventional	Washington	6	0.09265
393	46	Domestic	Conventional	Washington	7	0.09240
394	503	Domestic	Conventional	Washington	4	0.09177
395	465	Domestic	Conventional	Washington	4	0.09159
396	354	Domestic	Conventional	Maryland	4	0.09155
397	380	Domestic	Conventional	Washington	7	0.09121
398	594	Domestic	Conventional	Washington	4	0.09063
399	283	Domestic	Conventional	Washington	6	0.08864
400	202	Domestic	Conventional	Washington	6	0.08854
401	543	Domestic	Conventional	Washington	6	0.08846
402	254	Domestic	Conventional	Washington	7	0.08825
403	664	Domestic	Conventional	Washington	4	0.08807
404	11	Domestic	Conventional	Washington	4	0.08783
405	18	Domestic	Conventional	Washington	4	0.08778
406	303	Domestic	Conventional	Texas	6	0.08742
407	316	Domestic	Conventional	Washington	3	0.08739
408	232	Domestic	Conventional	Washington	7	0.08706
409	573	Domestic	Conventional	Washington	4	0.08681
410	197	Domestic	Conventional	Washington	5	0.08592
411	68	Domestic	Conventional	Washington	6	0.08575
412	386	Domestic	Conventional	Washington	6	0.08495
413	263	Domestic	Conventional	Washington	7	0.08472
414	484	Domestic	Conventional	Washington	3	0.08395
415	69	Domestic	Conventional	Washington	5	0.08391
416	148	Domestic	Conventional	Washington	4	0.08381

US-PDP: Individual Positive Samples, Ranked by Aggregate Sample DRI (Highest - Lowest), for Domestic Conventional Apples Tested in 2009

Ranking	PDP Sample ID	Origin	Market Claim	State	Number of Residues	Aggregate Sample DRI
417	634	Domestic	Conventional	Washington	8	0.08333
418	365	Domestic	Conventional	Washington	4	0.08307
419	478	Domestic	Conventional	California	4	0.08294
420	663	Domestic	Conventional	Michigan	2	0.08279
421	621	Domestic	Conventional	Washington	7	0.08276
422	1	Domestic	Conventional	Washington	4	0.08244
423	177	Domestic	Conventional	Texas	5	0.08223
424	243	Domestic	Conventional	Washington	5	0.08215
425	349	Domestic	Conventional	Washington	6	0.08212
426	318	Domestic	Conventional	New York	5	0.08187
427	707	Domestic	Conventional	Washington	3	0.08147
428	50	Domestic	Conventional	Washington	9	0.08101
429	382	Domestic	Conventional	Washington	5	0.08097
430	100	Domestic	Conventional	Washington	5	0.08058
431	418	Domestic	Conventional	Washington	6	0.07972
432	522	Domestic	Conventional	Washington	4	0.07971
433	506	Domestic	Conventional	Ohio	8	0.07963
434	516	Domestic	Conventional	Washington	6	0.07920
435	60	Domestic	Conventional	Washington	5	0.07896
436	53	Domestic	Conventional	Washington	7	0.07878
437	139	Domestic	Conventional	Washington	5	0.07854
438	40	Domestic	Conventional	Idaho	6	0.07754
439	474	Domestic	Conventional	Washington	4	0.07741
440	431	Domestic	Conventional	Washington	4	0.07504
441	224	Domestic	Conventional	Washington	10	0.07502
442	21	Domestic	Conventional	Washington	1	0.07450
443	558	Domestic	Conventional	Washington	5	0.07427
444	280	Domestic	Conventional	Pennsylvania	3	0.07426
445	304	Domestic	Conventional	Washington	4	0.07421
446	51	Domestic	Conventional	Washington	3	0.07294
447	379	Domestic	Conventional	Michigan	5	0.07248
448	443	Domestic	Conventional	New York	8	0.07227

US-PDP: Individual Positive Samples, Ranked by Aggregate Sample DRI (Highest - Lowest), for Domestic Conventional Apples Tested in 2009

Ranking	PDP Sample ID	Origin	Market Claim	State	Number of Residues	Aggregate Sample DRI
449	407	Domestic	Conventional	New York	7	0.07191
450	240	Domestic	Conventional	Washington	5	0.07092
451	140	Domestic	Conventional	Washington	3	0.07036
452	227	Domestic	Conventional	Washington	6	0.06996
453	469	Domestic	Conventional	Washington	5	0.06969
454	694	Domestic	Conventional	Washington	2	0.06884
455	712	Domestic	Conventional	Maryland	4	0.06859
456	212	Domestic	Conventional	Massachusetts	6	0.06844
457	487	Domestic	Conventional	New York	7	0.06840
458	333	Domestic	Conventional	Michigan	6	0.06821
459	628	Domestic	Conventional	Washington	4	0.06754
460	547	Domestic	Conventional	Indiana	9	0.06710
461	20	Domestic	Conventional	Washington	5	0.06693
462	348	Domestic	Conventional	Washington	4	0.06582
463	708	Domestic	Conventional	Washington	3	0.06529
464	169	Domestic	Conventional	Washington	4	0.06522
465	534	Domestic	Conventional	Washington	9	0.06497
466	397	Domestic	Conventional	New York	6	0.06490
467	172	Domestic	Conventional	Washington	4	0.06479
468	482	Domestic	Conventional	New York	7	0.06451
469	39	Domestic	Conventional	Washington	3	0.06428
470	94	Domestic	Conventional	Washington	4	0.06399
471	620	Domestic	Conventional	Washington	5	0.06319
472	328	Domestic	Conventional	Maryland	4	0.06270
473	445	Domestic	Conventional	Washington	5	0.06243
474	110	Domestic	Conventional	Washington	5	0.06234
475	305	Domestic	Conventional	Maryland	1	0.06208
476	300	Domestic	Conventional	Washington	4	0.06202
477	353	Domestic	Conventional	Washington	4	0.06181
478	403	Domestic	Conventional	Washington	4	0.06103
479	726	Domestic	Conventional	Michigan	7	0.06061
480	514	Domestic	Conventional	Michigan	5	0.05995

US-PDP: Individual Positive Samples, Ranked by Aggregate Sample DRI (Highest - Lowest), for Domestic Conventional Apples Tested in 2009

Ranking	PDP Sample ID	Origin	Market Claim	State	Number of Residues	Aggregate Sample DRI
481	561	Domestic	Conventional	Washington	5	0.05991
482	743	Domestic	Conventional	Michigan	5	0.05905
483	556	Domestic	Conventional	Washington	6	0.05876
484	57	Domestic	Conventional	Washington	4	0.05829
485	390	Domestic	Conventional	Michigan	2	0.05795
486	63	Domestic	Conventional	Washington	5	0.05730
487	9	Domestic	Conventional	Washington	5	0.05725
488	434	Domestic	Conventional	New York	5	0.05667
489	603	Domestic	Conventional	Washington	9	0.05636
490	26	Domestic	Conventional	California	3	0.05616
491	740	Domestic	Conventional	California	4	0.05599
492	278	Domestic	Conventional	Washington	8	0.05599
493	429	Domestic	Conventional	New York	7	0.05520
494	657	Domestic	Conventional	Unknown	4	0.05510
495	549	Domestic	Conventional	Ohio	4	0.05488
496	586	Domestic	Conventional	Washington	3	0.05435
497	45	Domestic	Conventional	Washington	3	0.05392
498	137	Domestic	Conventional	Idaho	2	0.05364
499	284	Domestic	Conventional	Texas	3	0.05304
500	118	Domestic	Conventional	Washington	5	0.05230
501	206	Domestic	Conventional	Washington	5	0.05119
502	176	Domestic	Conventional	Washington	8	0.05045
503	322	Domestic	Conventional	Washington	5	0.05027
504	61	Domestic	Conventional	Washington	4	0.04998
505	575	Domestic	Conventional	Texas	4	0.04991
506	737	Domestic	Conventional	Michigan	8	0.04895
507	275	Domestic	Conventional	Pennsylvania	6	0.04888
508	462	Domestic	Conventional	New York	9	0.04883
509	494	Domestic	Conventional	Washington	3	0.04850
510	251	Domestic	Conventional	Virginia	8	0.04820
511	400	Domestic	Conventional	New York	11	0.04743
512	526	Domestic	Conventional	Washington	6	0.04680

US-PDP: Individual Positive Samples, Ranked by Aggregate Sample DRI (Highest - Lowest), for Domestic Conventional Apples Tested in 2009

Ranking	PDP Sample ID	Origin	Market Claim	State	Number of Residues	Aggregate Sample DRI
513	720	Domestic	Conventional	Maryland	5	0.04628
514	682	Domestic	Conventional	Maryland	4	0.04537
515	475	Domestic	Conventional	Washington	3	0.04526
516	518	Domestic	Conventional	Washington	6	0.04484
517	329	Domestic	Conventional	Washington	5	0.04471
518	342	Domestic	Conventional	Washington	7	0.04443
519	552	Domestic	Conventional	Ohio	5	0.04346
520	472	Domestic	Conventional	New York	5	0.04313
521	507	Domestic	Conventional	Washington	4	0.04299
522	55	Domestic	Conventional	Washington	5	0.04297
523	83	Domestic	Conventional	Washington	4	0.04221
524	742	Domestic	Conventional	Washington	5	0.04205
525	560	Domestic	Conventional	Michigan	8	0.03940
526	433	Domestic	Conventional	New York	3	0.03902
527	377	Domestic	Conventional	Michigan	7	0.03870
528	600	Domestic	Conventional	Texas	8	0.03833
529	411	Domestic	Conventional	Washington	3	0.03781
530	583	Domestic	Conventional	Washington	10	0.03708
531	331	Domestic	Conventional	Michigan	4	0.03637
532	430	Domestic	Conventional	New York	7	0.03538
533	393	Domestic	Conventional	Washington	3	0.03337
534	566	Domestic	Conventional	Washington	5	0.03308
535	721	Domestic	Conventional	Wisconsin	5	0.03266
536	292	Domestic	Conventional	Texas	2	0.03228
537	282	Domestic	Conventional	Washington	5	0.03191
538	256	Domestic	Conventional	Washington	5	0.03191
539	186	Domestic	Conventional	Washington	6	0.03172
540	75	Domestic	Conventional	Washington	3	0.03164
541	414	Domestic	Conventional	Washington	5	0.03096
542	489	Domestic	Conventional	New York	10	0.03058
543	546	Domestic	Conventional	Michigan	7	0.03022
544	396	Domestic	Conventional	New York	9	0.03017

US-PDP: Individual Positive Samples, Ranked by Aggregate Sample DRI (Highest - Lowest), for Domestic Conventional Apples Tested in 2009

Ranking	PDP Sample ID	Origin	Market Claim	State	Number of Residues	Aggregate Sample DRI
545	448	Domestic	Conventional	Washington	8	0.02937
546	375	Domestic	Conventional	Maryland	2	0.02766
547	490	Domestic	Conventional	New York	3	0.02740
548	578	Domestic	Conventional	Washington	2	0.02675
549	309	Domestic	Conventional	Pennsylvania	2	0.02608
550	542	Domestic	Conventional	Washington	6	0.02542
551	467	Domestic	Conventional	Washington	6	0.02539
552	437	Domestic	Conventional	New York	4	0.02537
553	59	Domestic	Conventional	Washington	4	0.02533
554	389	Domestic	Conventional	California	4	0.02469
555	404	Domestic	Conventional	New York	4	0.02358
556	497	Domestic	Conventional	New York	5	0.02262
557	56	Domestic	Conventional	Washington	3	0.02252
558	680	Domestic	Conventional	Washington	4	0.02205
559	133	Domestic	Conventional	Washington	3	0.02185
560	211	Domestic	Conventional	Washington	5	0.02178
561	366	Domestic	Conventional	Washington	6	0.02155
562	466	Domestic	Conventional	New York	6	0.02153
563	595	Domestic	Conventional	Washington	10	0.02143
564	460	Domestic	Conventional	Washington	4	0.02074
565	7	Domestic	Conventional	Idaho	1	0.02069
566	130	Domestic	Conventional	Washington	1	0.02069
567	376	Domestic	Conventional	Unknown	1	0.02018
568	589	Domestic	Conventional	Washington	6	0.01983
569	273	Domestic	Conventional	New York	7	0.01919
570	537	Domestic	Conventional	Washington	6	0.01862
571	173	Domestic	Conventional	Washington	7	0.01834
572	144	Domestic	Conventional	California	2	0.01821
573	709	Domestic	Conventional	Washington	5	0.01810
574	613	Domestic	Conventional	Washington	5	0.01747
575	378	Domestic	Conventional	Michigan	5	0.01732
576	155	Domestic	Conventional	Washington	5	0.01719

US-PDP: Individual Positive Samples, Ranked by Aggregate Sample DRI (Highest - Lowest), for Domestic Conventional Apples Tested in 2009

Ranking	PDP Sample ID	Origin	Market Claim	State	Number of Residues	Aggregate Sample DRI
577	678	Domestic	Conventional	Washington	3	0.01691
578	388	Domestic	Conventional	Washington	5	0.01616
579	424	Domestic	Conventional	Washington	8	0.01612
580	660	Domestic	Conventional	Michigan	4	0.01588
581	685	Domestic	Conventional	Washington	8	0.01553
582	22	Domestic	Conventional	Washington	2	0.01548
583	216	Domestic	Conventional	Washington	9	0.01509
584	141	Domestic	Conventional	California	2	0.01502
585	30	Domestic	Conventional	Washington	5	0.01431
586	457	Domestic	Conventional	Washington	6	0.01352
587	387	Domestic	Conventional	Michigan	3	0.01301
588	550	Domestic	Conventional	Michigan	5	0.01273
589	476	Domestic	Conventional	Washington	6	0.01269
590	515	Domestic	Conventional	Ohio	7	0.01266
591	34	Domestic	Conventional	Washington	6	0.01184
592	142	Domestic	Conventional	Washington	3	0.01101
593	486	Domestic	Conventional	Washington	4	0.01088
594	234	Domestic	Conventional	Washington	8	0.01088
595	323	Domestic	Conventional	Unknown	5	0.01062
596	668	Domestic	Conventional	Washington	6	0.00973
597	184	Domestic	Conventional	Washington	5	0.00913
598	320	Domestic	Conventional	Michigan	2	0.00825
599	281	Domestic	Conventional	Pennsylvania	5	0.00784
600	330	Domestic	Conventional	Washington	6	0.00760
601	95	Domestic	Conventional	Washington	4	0.00710
602	164	Domestic	Conventional	Washington	2	0.00695
603	317	Domestic	Conventional	Washington	2	0.00689
604	119	Domestic	Conventional	Washington	2	0.00689
605	340	Domestic	Conventional	Washington	4	0.00684
606	205	Domestic	Conventional	Washington	5	0.00657
607	310	Domestic	Conventional	Washington	2	0.00619
608	658	Domestic	Conventional	Washington	1	0.00582

US-PDP: Individual Positive Samples, Ranked by Aggregate Sample DRI (Highest - Lowest), for Domestic Conventional Apples Tested in 2009

Ranking	PDP Sample ID	Origin	Market Claim	State	Number of Residues	Aggregate Sample DRI
609	711	Domestic	Conventional	Washington	6	0.00578
610	257	Domestic	Conventional	Washington	4	0.00560
611	259	Domestic	Conventional	Washington	4	0.00544
612	200	Domestic	Conventional	Washington	5	0.00520
613	289	Domestic	Conventional	Washington	4	0.00502
614	161	Domestic	Conventional	California	1	0.00497
615	744	Domestic	Conventional	Michigan	3	0.00495
616	665	Domestic	Conventional	Washington	3	0.00487
617	129	Domestic	Conventional	Washington	2	0.00482
618	588	Domestic	Conventional	Washington	4	0.00470
619	381	Domestic	Conventional	Washington	6	0.00453
620	325	Domestic	Conventional	Washington	3	0.00451
621	385	Domestic	Conventional	Michigan	5	0.00423
622	105	Domestic	Conventional	Washington	4	0.00403
623	491	Domestic	Conventional	New York	3	0.00397
624	166	Domestic	Conventional	Washington	3	0.00381
625	258	Domestic	Conventional	Washington	4	0.00345
626	419	Domestic	Conventional	Washington	6	0.00342
627	455	Domestic	Conventional	New York	4	0.00337
628	569	Domestic	Conventional	Michigan	2	0.00334
629	653	Domestic	Conventional	Michigan	7	0.00330
630	149	Domestic	Conventional	Washington	3	0.00240
631	408	Domestic	Conventional	New York	4	0.00236
632	10	Domestic	Conventional	Washington	2	0.00218
633	311	Domestic	Conventional	Washington	2	0.00218
634	644	Domestic	Conventional	Washington	4	0.00218
635	667	Domestic	Conventional	Washington	4	0.00213
636	337	Domestic	Conventional	Michigan	4	0.00208
637	696	Domestic	Conventional	Washington	4	0.00204
638	576	Domestic	Conventional	Washington	4	0.00202
639	564	Domestic	Conventional	Michigan	2	0.00186
640	185	Domestic	Conventional	Washington	4	0.00176

US-PDP: Individual Positive Samples, Ranked by Aggregate Sample DRI (Highest - Lowest), for Domestic Conventional Apples Tested in 2009

Ranking	PDP Sample ID	Origin	Market Claim	State	Number of Residues	Aggregate Sample DRI
641	346	Domestic	Conventional	Washington	3	0.00169
642	134	Domestic	Conventional	Washington	3	0.00164
643	640	Domestic	Conventional	Washington	4	0.00157
644	723	Domestic	Conventional	Michigan	3	0.00156
645	501	Domestic	Conventional	Michigan	2	0.00139
646	145	Domestic	Conventional	California	1	0.00124
647	332	Domestic	Conventional	Washington	4	0.00102
648	683	Domestic	Conventional	Washington	2	0.00097
649	315	Domestic	Conventional	Washington	1	0.00094
650	188	Domestic	Conventional	Washington	3	0.00094
651	659	Domestic	Conventional	Unknown	2	0.00088
652	661	Domestic	Conventional	Washington	1	0.00085
653	179	Domestic	Conventional	California	2	0.00076
654	374	Domestic	Conventional	Washington	3	0.00075
655	577	Domestic	Conventional	Washington	2	0.00067
656	112	Domestic	Conventional	California	1	0.00041
657	646	Domestic	Conventional	Washington	1	0.00038
658	477	Domestic	Conventional	Washington	1	0.00038
659	473	Domestic	Conventional	New York	1	0.00038
660	253	Domestic	Conventional	Texas	1	0.00038
661	138	Domestic	Conventional	Washington	1	0.00031
662	512	Domestic	Conventional	Michigan	1	0.00030
663	464	Domestic	Conventional	Washington	2	0.00029
664	384	Domestic	Conventional	Washington	1	0.00021
665	65	Domestic	Conventional	Washington	1	0.00018
666	28	Domestic	Conventional	Washington	1	0.00014

Number of Positive Samples: 666

Total Number of Residues and Average Aggregate DRI: 3,522 0.2220
