# Supplementary material for: The dietary risk index system: a tool to track pesticide dietary risks
Source: Environ Health. 2020 Oct 14;19:103. doi: 10.1186/s12940-020-00657-z (PMC7557078; doi:10.1186/s12940-020-00657-z)
Supplement: Supplementary file 11 — Additional file 11. Pesticide Residues and DRI values in Conventionally and Organically Grown Apples, US-PDP, 2009. [file 12940_2020_657_MOESM11_ESM.pdf]

Additional File 11 for "The Dietary Risk Index System: A Tool to Track Pesticide Dietary Risks," by Benbrook and Davis

Table 1: Pesticide Residue and Risk Indicators in Apples Ranked by Percent of Aggregate FS-DRI: Conventionally Grown Samples, Domestically Grown Apples Tested by PDP in 2009

Pesticide	Total Number of Samples Tested	Number of Positives	Percent Positive	Mean Residue (ppm)	cRfC (ppm)	DRI-M	FS-DRI	Percent of Aggregate FS-DRI
Thiabendazole	672	523	77.8%	0.394	5.32	0.0742	0.0577	25.9%
Diazinon	672	61	9.08%	0.0148	0.0322	0.460	0.0418	18.7%
Azinphos methyl	672	130	19.3%	0.0412	0.242	0.170	0.0330	14.8%
Diphenylamine (DPA)	672	516	76.8%	0.364	16.1	0.0226	0.0173	7.77%
Dicofol p,p'	672	1	0.149%	0.560	0.0644	8.69	0.0129	5.80%
Pyrimethanil	672	394	58.6%	0.501	27.4	0.0183	0.0107	4.80%
Phosmet	672	100	14.9%	0.0637	0.966	0.0659	0.00980	4.39%
Formetanate hydrochloride	672	109	16.2%	0.00270	0.0515	0.0524	0.00851	3.81%
Chlorpyrifos	672	3	0.446%	0.0592	0.0483	1.22	0.00547	2.45%
Fludioxonil	672	67	9.97%	0.215	4.83	0.0444	0.00443	1.99%
Endosulfan II	669	94	14.1%	0.0234	0.966	0.0242	0.00339	1.52%
Endosulfan I	672	51	7.59%	0.0293	0.966	0.0304	0.00230	1.03%
Tetrahydrophthalimide (THPI)	672	111	16.5%	0.262	20.1	0.0130	0.00215	0.964%
Endosulfan sulfate	657	53	8.07%	0.0236	0.966	0.0244	0.00197	0.883%
Carbendazim (MBC)	484	103	21.3%	0.0343	4.03	0.0085	0.00181	0.812%
Esfenvalerate+Fenvalerate Total	484	5	1.03%	0.0486	0.290	0.168	0.00173	0.776%
Cyhalothrin, Total	484	2	0.413%	0.0450	0.161	0.279	0.00115	0.517%
Cyhalothrin, Lambda	188	3	1.60%	0.0100	0.161	0.0621	0.000991	0.444%
Thiacloprid	672	48	7.14%	0.00693	0.644	0.0108	0.000768	0.344%
Pyraclostrobin	672	118	17.6%	0.0201	5.48	0.00368	0.000646	0.289%
Acetamiprid	672	231	34.4%	0.0212	11.4	0.00185	0.000637	0.2854%
Captan	188	17	9.04%	0.146	20.9	0.00698	0.000631	0.283%
Carbaryl	672	23	3.42%	0.0267	1.61	0.0165	0.000566	0.254%
Fenpropathrin	672	25	3.72%	0.0881	8.05	0.0109	0.000407	0.182%
Cyhalothrin, Lambda epimer R1578	188	1	0.532%	0.0100	0.161	0.0621	0.000330	0.148%
Fenpyroximate	484	69	14.26%	0.0154	8.05	0.00191	0.000272	0.122%
Diflubenzuron	672	23	3.42%	0.0252	3.22	0.00781	0.000267	0.120%
Dimethoate	672	2	0.298%	0.0305	0.35	0.0861	0.000256	0.115%
Boscalid	672	134	19.9%	0.0408	35.11	0.00116	0.000232	0.104%
Pyridaben	188	1	0.532%	0.0250	0.805	0.0310	0.000165	0.0740%
Buprofezin	672	3	0.45%	0.0143	0.532	0.0268	0.000120	0.0537%
Imidacloprid	672	126	18.8%	0.00501	9.2	0.000546	0.000102	0.0459%
Hexythiazox	188	6	3.19%	0.0113	4.03	0.00281	0.0000898	0.0403%
Omethoate	672	2	0.298%	0.00800	0.354	0.0226	0.0000672	0.0301%
1-Naphthol	188	1	0.532%	0.0200	1.61	0.0124	0.0000660	0.0296%
Methoxyfenozide	672	81	12.1%	0.00748	16.1	0.000464	0.0000560	0.0251%
Methomyl	672	2	0.298%	0.0200	1.29	0.0155	0.0000462	0.0207%
Myclobutanil	672	23	3.42%	0.00453	4.03	0.00113	0.0000385	0.0173%
5-Hydroxythiabendazole	188	4	2.13%	0.00825	5.32	0.00155	0.0000330	0.015%
Trifloxystrobin	672	27	4.02%	0.00491	6.12	0.000803	0.0000323	0.0145%
Spinetoram	472	18	3.81%	0.00308	4.01	0.000767	0.0000293	0.0131%
Etoxazole	484	18	3.72%	0.00468	7.41	0.000631	0.0000235	0.0105%
Propargite	672	4	0.595%	0.0208	6.44	0.00322	0.0000192	0.00859%
Fenbuconazole	672	6	0.893%	0.0100	4.83	0.00207	0.0000185	0.00828%
Chlorantraniliprole	484	152	31.4%	0.00963	254	0.0000378	0.0000119	0.00532%
Chlorpropham	672	3	0.446%	0.0170	8.05	0.00211	0.00000942	0.00422%
Spinosad A	188	2	1.06%	0.00300	4.01	0.000748	0.00000796	0.00357%
Flonicamid	672	4	0.595%	0.00855	6.44	0.00133	0.00000790	0.00354%
Pendimethalin	672	11	1.64%	0.00703	48.3	0.000145	0.00000238	0.00107%
Piperonyl butoxide	660	1	0.152%	0.0310	25.0	0.00124	0.00000188	0.000843%
O-Phenylphenol	672	9	1.34%	0.00798	62.8	0.000127	0.00000170	0.000762%
Permethrin cis	672	1	0.149%	0.00400	40.3	0.000099	0.000000148	0.0000662%

Table 2: Pesticide Residue and Risk Indicators in Apples Ranked by Percent of Aggregate FS-DRI: Organically Grown Samples, Domestically Grown Apples Tested by PDP in 2009

Pesticide	Total Number of Samples Tested	Number of Positives	Percent Positive	Mean Residue (ppm)	cRfC (ppm)	DRI-M	FS-DRI	Percent of Aggregate FS-DRI
Diphenylamine (DPA)	28	10	35.7%	0.0113	16.1	0.000702	0.000251	28.7%
Thiabendazole	28	9	32.1%	0.004233	5.32	0.000796	0.000256	29.3%
Spinosad A	8	2	25.0%	0.00470	4.01	0.001172	0.000293	33.5%
Pyrimethanil	28	12	42.9%	0.003183	27.4	0.000116	0.0000498	5.70%
Spinosad Total	20	1	5.00%	0.00200	4.01	0.000499	0.0000249	2.85%
